# Quantitative Comparison of Catalytic Mechanisms and Overall Reactions in Convergently Evolved Enzymes: Implications for Classification of Enzyme Function

**DOI:** 10.1371/journal.pcbi.1000700

**Published:** 2010-03-12

**Authors:** Daniel E. Almonacid, Emmanuel R. Yera, John B. O. Mitchell, Patricia C. Babbitt

**Affiliations:** 1 Department of Bioengineering and Therapeutic Sciences, University of California San Francisco, San Francisco, California, United States of America; 2 Department of Pharmaceutical Chemistry, University of California San Francisco, San Francisco, California, United States of America; 3 California Institute for Quantitative Biosciences, University of California San Francisco, San Francisco, California, United States of America; 4 Biological and Medical Informatics Graduate Program, University of California San Francisco, San Francisco, California, United States of America; 5 Centre for Biomolecular Sciences, University of St Andrews, St Andrews, United Kingdom; University College London, United Kingdom

## Abstract

Functionally analogous enzymes are those that catalyze similar reactions on similar substrates but do not share common ancestry, providing a window on the different structural strategies nature has used to evolve required catalysts. Identification and use of this information to improve reaction classification and computational annotation of enzymes newly discovered in the genome projects would benefit from systematic determination of reaction similarities. Here, we quantified similarity in bond changes for overall reactions and catalytic mechanisms for 95 pairs of functionally analogous enzymes (non-homologous enzymes with identical first three numbers of their EC codes) from the MACiE database. Similarity of overall reactions was computed by comparing the sets of bond changes in the transformations from substrates to products. For similarity of mechanisms, sets of bond changes occurring in each mechanistic step were compared; these similarities were then used to guide global and local alignments of mechanistic steps. Using this metric, only 44% of pairs of functionally analogous enzymes in the dataset had significantly similar overall reactions. For these enzymes, convergence to the same mechanism occurred in 33% of cases, with most pairs having at least one identical mechanistic step. Using our metric, overall reaction similarity serves as an upper bound for mechanistic similarity in functional analogs. For example, the four carbon-oxygen lyases acting on phosphates (EC 4.2.3) show neither significant overall reaction similarity nor significant mechanistic similarity. By contrast, the three carboxylic-ester hydrolases (EC 3.1.1) catalyze overall reactions with identical bond changes and have converged to almost identical mechanisms. The large proportion of enzyme pairs that do not show significant overall reaction similarity (56%) suggests that at least for the functionally analogous enzymes studied here, more stringent criteria could be used to refine definitions of EC sub-subclasses for improved discrimination in their classification of enzyme reactions. The results also indicate that mechanistic convergence of reaction steps is widespread, suggesting that quantitative measurement of mechanistic similarity can inform approaches for functional annotation.

## Author Summary

When species evolve, their genes duplicate and diverge to allow for adaptation of their functional repertoires to the changing environment. In this scenario, unrelated genes can convergently evolve to produce proteins with the same molecular function, termed “functionally analogous.” A quantitative determination of the reaction similarities among functionally analogous enzymes could provide insight about the different structural solutions nature has used to evolve similar catalysts. Bond changes between substrates and products, and between successive reaction intermediates, were used to compare the reactions catalyzed and the mechanisms of catalysis for 95 pairs of functionally analogous enzymes. Less than half of the reactions catalyzed by unrelated enzymes, but defined as similar by the Enzyme Commission (EC) classification, are similar in terms of bond changes, suggesting that this classification often fails to capture quantitative differences between many enzyme reactions. Furthermore, we addressed for the first time whether the chemical mechanisms by which similar overall reactions are achieved in functional analogs are also similar. We conclude that convergence of reaction is often accompanied by convergence of chemical mechanism. These results will be useful for classifying enzymes, guiding functional annotation of newly determined enzyme sequences and structures and for informing the engineering of enzymes with new functions.

## Introduction

Using the Enzyme Commission (EC) classification to describe function [1] and the structure and sequence similarity between proteins as a measure of homology, numerous works have reported cases of divergence and convergence of function in enzymes [2]–[17]. During divergence of function, gene duplication and sequence divergence generate functionally different but structurally related proteins [3]–[7], [18]–[21]. During convergence of function, proteins that are product of non-homologous genes, and therefore not related in sequence or structure, independently evolve to converge in performing the same (or similar) overall reactions on the same (or similar) substrates. Convergent evolution was first described 40 years ago by Wright and colleagues in their article reporting the crystal structure of subtilisin [22]. As a note in proof, it was observed that the three hydrogen-bonded catalytic residues in the active site of subtilisin were also present in the functionally similar serine protease chymotrypsin [23], leading them to hypothesize the involvement of the triad in the enzymatic mechanism of both proteases. This first example of convergence of active site and catalytic mechanism presaged subsequent findings that convergence of function in enzymes is widespread. Based in part on the observation that more enzyme superfamilies have been identified than enzymatic functions known, some studies have concluded that in enzymes, convergence of function is more common than divergence [2],[9],[10],[14]. However, functionally analogous enzymes have been neglected in most studies, and as noted by Morris, it is still common to find adjectives such as “uncanny” and “surprising” to refer to the phenomenon of convergence [24]. As a consequence, several questions about the catalysis of similar overall reactions by different structural scaffolds have been poorly studied or not studied at all. Could it be that unrelated enzymes bind similar substrates carrying the same functional groups, but the reaction mechanisms vary significantly in detail? Or conversely, does the reaction chemistry converge, while the substrate specificity differs? Quantitatively, how similar or different are the catalytic mechanisms of functional analogs? Is at least a key mechanistic step shared among functional analogs? Do the enzymes have similar active sites? If not, how do different active sites perform similar overall reactions? Answering these questions may provide insight into the evolutionary constraints that overall reactions impose on the enzymes that catalyze them, specifically in their requirements of catalytic species (amino acid residues, organic cofactors and metal ions), reaction mechanisms, binding sites, and ultimately, their tertiary and quaternary structures. This knowledge could be used in many ways, for instance, to inform functional annotation of newly determined sequences and structures, and to select appropriate enzyme scaffolds for engineering new functions. It also allows us to address whether similarity of enzyme function according to the EC is reflected in shared reaction strategies, or even in shared structural characteristics such as active sites, and how the resulting information could be used to refine definitions in the current EC classification or propose alternative quantitative classifications for enzymes.

Recently, interest has reawakened in studying functionally analogous enzymes [11],[25] due in part to the availability of databases containing information about amino acid residues (Catalytic Site Atlas, CSA) [26] and metal cofactors (Metal-MACiE) [27],[28] involved in catalysis and the step-by-step reaction mechanisms of enzymes (MACiE [29],[30], SFLD [31],[32] and EzCatDB [33]). Using the catalytic residues annotated in the CSA [26], evolutionary information from the SCOP database [34], and a program that compares the positions of residues in protein structures, Query3d [35], Gherardini and colleagues investigated whether functionally analogous enzymes have similar active sites [11]. They found that enzymes catalyzing reactions in 110 out of the 169 different enzyme commission sub-subclasses (third level of the EC classification) analyzed in their study belonged to at least two different SCOP structural superfamilies, i.e. they were examples of convergence of function. Furthermore, they found that 24% (26 out of 110) of the sub-subclasses with examples of convergence of function were catalyzed by at least two non-homologous enzymes with structurally equivalent active site residues playing equivalent roles in catalysis (convergence of active site). They concluded that convergent evolution of active sites is not a rare phenomenon among functionally analogous enzymes.

Here, other unanswered questions regarding functionally analogous enzymes have been addressed. Specifically, we quantified similarity in bond changes in overall reactions and reaction steps for 95 pairs of functionally analogous enzymes (non-homologous enzymes with identical first three numbers of their EC codes) from the MACiE database. MACiE currently includes 223 reaction mechanisms for enzymes with both a structure deposited in the PDB [36] and a plausible reaction mechanism published in the literature [29],[30], information we required for this study. To compare these reactions, we used a method we recently developed to measure similarity between reactions based on their explicit mechanisms [37]. For that work, mechanistic steps in enzyme reactions were coded as sets of bond changes or fingerprints. Similarity between all possible combinations of steps among every pair of reactions was calculated using a Tanimoto coefficient [38] or a normalized Euclidean distance [39], respectively. Reaction sequences were globally aligned and another Tanimoto coefficient calculated to describe the similarity of each pair of reactions based on the aligned steps.

For the present work, we extended our method for comparing bond changes in pairs of enzymes to consider reversibility of enzyme reactions, to allow for circular permutation of steps in the reaction sequences, and to include local alignments (using the Smith-Waterman algorithm [40]). We first assessed whether the sub-subclass level of the EC classification, commonly used to define similarity of enzyme catalytic activity in this and other work, is indicative of overall reaction similarity for pairs of functionally analogous enzymes. We then compared the mechanistic steps of each pair of reactions in the dataset and looked for global and local alignments of the steps to determine the extent to which similarity of overall reaction entails similarity of the stepwise reaction mechanisms that describe each overall reaction. For those pairs of enzymes with similar overall reactions, convergence to the same mechanism was found in one third of the examples, with a subset of these pairs also having at least one identical mechanistic step. However, the results also indicated that over two-fifths of the EC sub-subclasses represented in the study contain pairs of enzymes whose overall reaction similarity is not significantly higher than that of pairs of non-homologous enzymes sharing two, one or none of the numbers of their EC codes.

## Results

### Overview of the Dataset and Methods

The dataset of functionally analogous enzymes was created from version 2.3.9 of the MACiE database [30]. Ninety-five pairs of enzymes (a total of 80 of 223 proteins included in MACiE) from the same sub-subclass level of the EC classification [1], but non-homologous according to the CATH database [41], were selected (Table S1). Although this set represents only a small proportion of the known examples of convergence of function existing in nature, it broadly samples those enzymes that have been both structurally and functionally characterized. The dataset contains enzymes from all EC classes and from 29 of the 190 different EC sub-subclasses for which there are enzymes of known structure. Similarly, the enzymes in the dataset represent all four CATH structural classes. To assess the significance of the results, a background dataset was assembled from all enzymes in MACiE that were not included in the dataset of functional analogs. Only one instance of each different EC sub-subclass and CATH code was maintained, so that pairs of enzymes were not related either by function or by structure. Thus, similarity of overall reactions and reaction mechanisms only occurs in the background dataset because of the limited repertoire of bond types involved in catalysis, rather than from evolutionary constraints. The background dataset consisted of 85 proteins (Table S2), forming a total of 3570 possible pairs of enzymes. Figure S1 plots the distribution of all currently defined EC sub-subclasses [42] and CATH superfamilies [41], the fraction of those present in enzymes of known structure [43], and those present in the dataset and background dataset.

**Figure 1:**
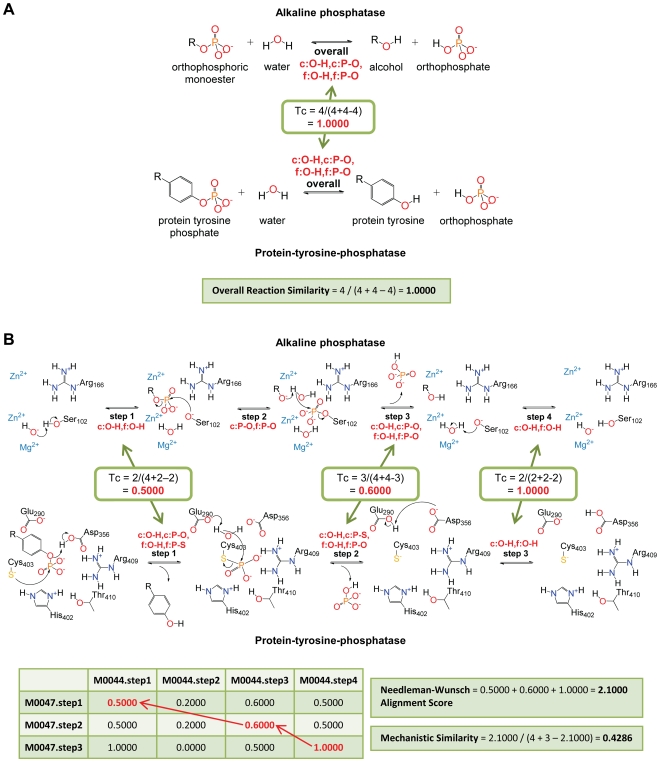
Quantification of overall reaction and mechanistic similarity. The reactions catalyzed by alkaline phosphatase (MACiE M0044, EC 3.1.3.1, PDB 1alk) [118]–[120], and protein-tyrosine-phosphatase (MACiE M0047, EC 3.1.3.48, PDB 1ytw) [121]–[123] are used as examples. Each reaction in MACiE is described as an overall transformation (A) and as a sequence of mechanistic steps (B). For measuring reaction similarity, each overall reaction and mechanistic step is represented as the set of bond changes occurring in the transformation from substrates to products, with c: bond cleaved, d: bond decreased in order, f: bond formed, and i: bond increased in order. Similarity between sets of bond changes is computed using Tanimoto coefficients (Tc). (A) Overall similarity is computed as the Tanimoto coefficient between the set of bond changes occurring in the transformation of substrates to products of the reactions. (B) Mechanistic similarity is computed from a global alignment of the mechanistic steps. First, Tanimoto coefficients between all possible pairs of steps are stored in a similarity matrix, and then the maximum-match pathway is obtained using the Needleman-Wunsch algorithm. To obtain the mechanistic similarity a new Tanimoto coefficient is computed using the number of steps in each reaction and the Needleman-Wunsch alignment score as inputs (see Methods).

Overall reaction similarity and mechanistic similarity for each pair of reactions in the dataset and background dataset were calculated based on bond change information (Figure 1, see Methods). Similarity scores for overall reactions were computed as Tanimoto coefficients between the sets of bond changes describing the transformation from substrates to products in each pair of reactions (Figure 1A). For mechanistic similarity, the bond changes in each individual mechanistic step of one reaction were compared to bond changes in all mechanistic steps of the other reaction in a pair using a Tanimoto coefficient (Figure 1B). Then, similarity scores for each possible combination of steps were stored in a similarity matrix and the Needleman-Wunsch algorithm [44] was used to obtain a score based on the best alignment of mechanistic steps. Finally, a new Tanimoto coefficient was computed based on the Needleman-Wunsch alignment score and the number of mechanistic steps in the reactions compared. Reversibility of reactions and circular permutations of mechanistic steps were considered explicitly in our calculations. The Smith-Waterman algorithm [40] was also implemented to look for local alignments of mechanistic steps (see Methods).

### Overall Reaction Similarity

**Figure 2:**
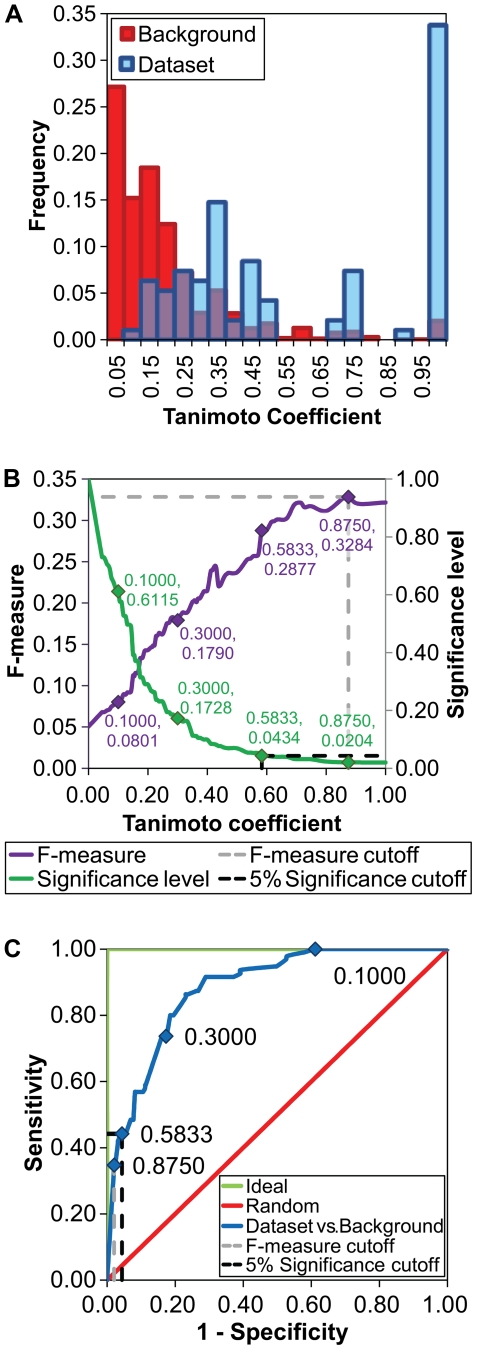
Overall reaction similarity. (A) Distribution of overall similarity scores for pairs of reactions in the background dataset (Background) and for the functionally analogous pairs in the dataset (Dataset). (B) F-measures and significance levels for all possible similarity scores. Selected overall similarity scores are shown within the plot, including the cutoff for similarity where the F-measure is maximized (0.8750), and the cutoff at the 5% significance level (0.5833). (C) ROC curves for the overall similarity scores of pairs of reactions in the dataset assessed against those in the background (Dataset vs. Background, AUC = 0.88), for an ideal classification method with no false positives and no false negatives (Ideal, AUC = 1.00), and for a non-discriminating classification method (Random, AUC = 0.50). Selected overall reaction similarity scores are shown within the curves.

It is commonplace in the literature to consider the EC sub-subclass (third number in the EC classification) as a description of the chemistry catalyzed by an enzyme [2],[9],[11],[12],[14],[45],[46]. In particular, this number specifies the acceptors, donors and groups that undergo transformation in enzyme reactions such that each sub-subclass is specific to a class and defines the type of acceptor (EC 1), other information about the group transferred (EC 2), nature of the substrate (EC 3, EC 5 and EC 6), or group eliminated (EC 4), respectively. Therefore, two enzymes in the same EC sub-subclass are expected to be functionally similar, i.e. catalyze a similar chemical reaction irrespective of substrate specificity (see Methods). Using similarity in bond changes to measure overall reaction similarity, we tested this assumption by assessing whether or not two enzymes in the same EC sub-subclass catalyze similar overall reactions. The results show that less than half of the pairs of reactions in our dataset of functional analogs share a significant number of bond changes in their overall reactions (see below). Table S3 provides values for the similarity of overall reactions for all 95 pairs of functionally analogous enzymes in the dataset. Figure 2A shows the distribution of overall reaction similarity scores in the dataset and in the background dataset. Figure 2B plots F-measures (harmonic mean of precision and recall, see Methods) and significance levels for all possible similarity scores, and Figure 2C plots a receiver operating characteristic (ROC) curve showing the true positive rate (sensitivity) *vs.* the false positive rate (1-specificity) for the different possible similarity scores. The true positive rate is the proportion of pairs in the dataset that score above a given cutoff similarity score, and the false positive rate is the proportion of pairs in the background dataset that score above the same cutoff similarity score (see Methods).

Based on their EC classifications, one might also expect that the overall reaction similarities between the 95 pairs of functionally analogous enzymes in the dataset should be higher than those similarities that occur between random pairs in the background dataset. This was true to a certain extent – at every similarity score the true positive rate was always higher than the false positive rate (Figure 2C), with the ROC curve resembling more the curve generated by an ideal classification method than that of a non-discriminating method. However, as the similarity score decreases, the enrichment factor (true positives divided by positive examples expected by random chance) decreases too. This means that the discrimination between true positives and false positives worsens as similarity scores get lower. To find an objective optimized cutoff for the overall similarity score above which there is certainty that the pairs of enzymes are significantly more similar than those in the background dataset, we looked at the score that maximizes the F-measure (Figure 2B, see Methods). The Tanimoto coefficient at this point was 0.8750 (Figure 2C), and the enrichment factor was 16.99. Using this cutoff, 34.7% (33 out of 95) of the functionally analogous pairs of enzymes from the dataset were retrieved as similar by our metric, compared to only 2.0% of those in the background dataset. This cutoff value was very stringent and all pairs of enzymes with an overall similarity score equal or higher than 0.8750 were called “highly similar.” An additional threshold score was also considered, that where similarity scores were significant at the 5% level. By allowing this increase in the number of false positives, the true positive rate increased to 44.2% (42 pairs). The Tanimoto coefficient at this point was 0.5833, and the enrichment factor was 10.18. The 9 pairs with scores lower than the F-measure optimized cutoff, but equal or higher than the cutoff at the 5% significance level were called “distantly similar.” In summary, we observed that only the 33 highly similar and the 9 distantly similar pairs of functionally analogous enzymes shared a sufficient number of bond changes to allow them to be identified as statistically significantly different from random pairs of reactions.

### Similar and Non-Similar Overall Reactions

Of the 42 pairs of overall reactions for which the similarity was statistically significant at the 5% significance level, 32 were identical in terms of bond changes, and seven consisted of a pair of reactions where the bond changes of one overall reaction were a perfect subset of the bond changes of the other. There were three cases where the bond changes in one overall reaction were a subset of the bond changes in the other, but the reactions were not found to be significantly similar (pairs M0045 and M0205, M0079 and M0214, and M0194 and M0206). This was due to one of the reactions containing ≤50% as many bond changes as the other reaction, leading to a decrease in the Tanimoto coefficient below the 5% significance level. Of the 42 pairs with significantly similar overall reactions, there were only three pairs where the bond changes of one reaction were neither identical nor a perfect subset of the bond changes of the other reaction (M0092 and M0093, M0112 and M0120, and M0075 and M0200).

In addition to the above 42 pairs, 53 pairs of enzymes had overall similarity scores lower than those of the top 5% of scores in the background dataset, and were thus regarded as non-similar. These pairs of reactions spanned 12 of the 29 EC sub-subclasses considered (groups 2 and 3 in Table 1, where group 1 consists only of EC sub-subclasses with similar overall reactions, group 2 consists of EC sub-subclasses with both similar and non-similar reactions, and group 3 consists only of EC sub-subclasses with non-similar reactions). That is, in the dataset, over two-fifths of the sub-subclasses defined by the Enzyme Commission included at least one pair of reactions with an overall similarity score not significantly higher than those of random pairs of reactions. In general, there was a good linear correlation between the number of pairs of enzymes in the EC sub-subclasses and the average proportion of pairs that were non-similar (R^2^ = 0.74). This indicated that according to our metric, EC sub-subclasses that map to several different structural scaffolds encompass a complex mixture of overall reactions, and may be better redefined as separate sub-subclasses. The most dramatic example is that of the carbon-oxygen lyases acting on phosphates (EC 4.2.3), for which none of the six pairs of reactions exhibited significant overall reaction similarity. By contrast, of the 19 sub-subclasses with only one pair of enzymes in our dataset, 13 had a pair with significant overall reaction similarity.

**Table 1:** Summary of results clustered according to overall reaction similarity.

EC sub-subclass	EC sub-subclass definition	Domain combi-nations PDB-SprotEC	Domain combinations dataset	Pairs in dataset	Pairs with similar overall reaction	Pairs with similar mechanism	Pairs with identical steps
**Group 1: Sub-subclasses containing only highly similar and/or distantly similar overall reactions**							
1.3.99	Oxidoreductases; Acting on the CH-CH group of donors; With other acceptors	2	2	1	1	0	0
1.5.1	Oxidoreductases; Acting on the CH-NH group of donors; With NAD+ or NADP+ as acceptor	9	2	1	1	0	0
2.2.1	Transferases; Transferring aldehyde or ketonic groups; Transketolases and transaldolases	2	2	1	1	0	0
2.3.3	Transferases; Acyltransferases; Acyl groups converted into alkyl groups on transfer	2	2	1	1	1	1
2.6.1	Transferases; Transferring nitrogenous groups; Transaminases	4	2	1	1	0	1
3.1.1	Hydrolases; Acting on ester bonds; Carboxylic-ester hydrolases	13	3	3	3	3	3
3.1.3	Hydrolases; Acting on ester bonds; Phosphoric-monoester hydrolases	24	3	3	3	1	1
3.1.4	Hydrolases; Acting on ester bonds; Phosphoric-diester hydrolases	11	2	1	1	1	0
3.1.21	Hydrolases; Acting on ester bonds; Endodeoxyribonucleases producing 5′-phosphomonoesters	10	2	1	1	0	0
3.2.1	Hydrolases; Glycosylases; Glycosidases, i.e. hydrolysing O- and S-glycosyl compounds	21	2	1	1	1	0
3.5.1	Hydrolases; Acting on carbon-nitrogen bonds, other than peptide bonds; In linear amides	12	4	6	6	2	1
3.5.2	Hydrolases; Acting on carbon-nitrogen bonds, other than peptide bonds; In cyclic amides	5	2	1	1	1	1
3.8.1	Hydrolases; Acting on halide bonds; In carbon-halide compounds	2	2	1	1	0	0
4.1.2	Lyases; Carbon-carbon lyases; Aldehyde-lyases	5	3	3	3	1	0
4.6.1	Lyases; Phosphorus-oxygen lyases; Phosphorus-oxygen lyases	4	2	1	1	0	0
5.1.1	Isomerases; Racemases and epimerases; Acting on amino acids and derivates	4	2	1	1	0	0
6.3.1	Ligases; Forming carbon-nitrogen bonds; Acid-ammonia (or amine) ligases (amide synthases)	3	2	1	1	1	1
**Group 2: Sub-subclasses containing highly similar and/or distantly similar plus non-similar overall reactions**							
1.1.1	Oxidoreductases; Acting on the CH-OH group of donors; With NAD+ or NADP+ as acceptor	11	3	3	1	2	3
2.3.1	Transferases; Acyltransferases; Transferring groups other than aminoacyl groups	25	4	6	1	0	1
2.4.2	Transferases; Glycosyltransferases; Pentosyltransferases	15	6	15	3	2	4
4.1.1	Lyases; Carbon-carbon lyases; Carboxy-lyases	20	5	10	6	1	1
4.2.1	Lyases; Carbon-oxygen lyases; Hydro-lyases	24	7	21	3	1	3
**Group 3: Sub-subclasses containing only non-similar overall reactions**							
2.1.1	Transferases; Transferring one-carbon groups; Methyltransferases	14	2	1	0	0	0
2.4.1	Transferases; Glycosyltransferases; Hexosyltransferases	4	2	1	0	0	0
3.2.2	Hydrolases; Glycosylases; Hydrolysing N-glycosyl compounds	6	2	1	0	1	0
3.5.4	Hydrolases; Acting on carbon-nitrogen bonds, other than peptide bonds; In cyclic amidines	8	2	1	0	0	0
4.2.3	Lyases; Carbon-oxygen lyases; Acting on phosphates	5	4	6	0	0	0
5.4.2	Isomerases; Intramolecular transferases; Phosphotransferases (phosphomutases)	5	2	1	0	0	0
5.4.99	Isomerases; Intramolecular transferases; Transferring other groups	6	2	1	0	0	0

EC classes with more than one sub-subclass in the dataset populated two or three of the groups in Table 1. That is, all EC classes have sub-subclasses containing similar and non-similar overall reactions, apart from the ligases for which there is only one sub-subclass contained in the dataset. Looking at a finer granularity, we observed that all pairs were significantly similar in all four sub-subclasses of the hydrolases acting on ester bonds subclass (EC 3.1). Examples are the overall reactions catalyzed by the carboxylic-ester hydrolases (EC 3.1.1) (Figure S2). By contrast, both sub-subclasses of the intramolecular transferases subclass (EC 5.4) had pairs that were significantly different from each other. We also looked at the bond changes shared across all members of each sub-subclass to investigate what bond types were involved (Figure S3). Six of the seven bond types most commonly involved in enzyme catalysis, i.e. O-H, C-O, N-H, C-C, C-N, and P-O (1^st^, 2^nd^, 3^rd^, 4^th^, 5^th^, and 7^th^ most common according to [47]) were shared in EC sub-subclasses with or without significantly similar overall reactions (all groups in Table 1). Less common bond changes, involving C-H, S-H and C-S bond types (6^th^, 8^th^ and 10^th^ most common in [47]) were shared in EC sub-subclasses in which at least some pairs of overall reactions were significantly similar (groups 1 and 2 in Table 1). Finally, C-Cl bond changes (37^th^ most common in [47]) were only catalyzed by an EC sub-subclass in which overall reactions were significantly similar (EC 3.8.1 in group 1 in Table 1). Thus, in the dataset, sharing of rare bond changes alone was a strong indicator of high overall reaction similarity. The results also suggest that the additional bond changes associated with less common bond types (e.g. the C-O bond formation and the O-H bond cleavage that are associated with C-Cl bond cleavage in the overall reactions in EC 3.8.1) are more conserved than those associated with more common bond types.

We also observed that the 12 sub-subclasses with non-similar overall reactions (groups 2 and 3 in Table 1) contained reactions which were on average larger and more dissimilar in terms of the number of bond changes than those in the sub-subclasses containing only similar overall reactions (Table S4). Therefore, some reactions in these 12 sub-subclasses contain extra bond changes that render them significantly different from other overall reactions in the same sub-subclass. To avoid penalizing the sets of bonds compared for differences in size, each Tanimoto coefficient was normalized by the maximum possible similarity given the number of bond changes contained in the reactions compared (see Methods). The cutoff at the 5% significance level for the normalized overall similarity scores was 1.0000, and only those 42 pairs where one reaction was a perfect match or a subset of the other reaction (see above) were considered to be significantly similar (Table S3). Of those 42 pairs, 39 were already considered highly or distantly similar according to the original measure of overall reaction similarity, showing that the extra bond changes alone are not responsible for the differences we identified in non-similar overall reactions.

### Mechanistic Similarity

**Figure 3:**
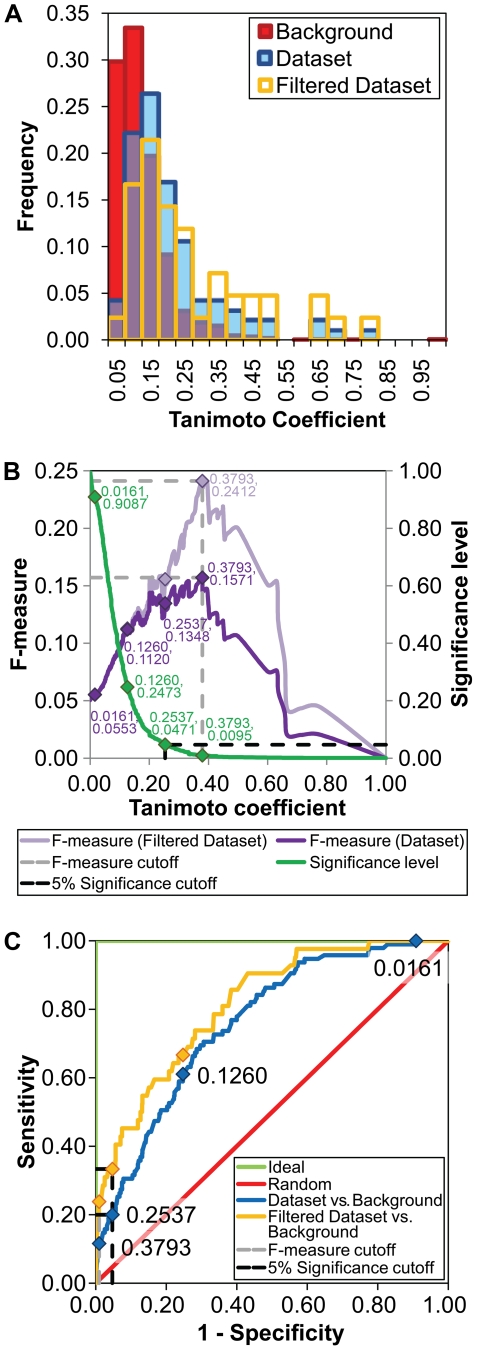
Mechanistic similarity. (A) Distribution of mechanistic similarity scores for pairs of reactions in the background dataset (Background), for all functionally analogous pairs in the dataset (Dataset), and for functionally analogous pairs with high overall reaction similarity (Filtered Dataset). (B) F-measures for the dataset and filtered dataset, and significance levels at all possible mechanistic similarity scores. Selected scores are shown within the plot, including the cutoff for similarity where the F-measure is maximized (0.3793), and the cutoff at the 5% significance level (0.2537). (C) ROC curves for the mechanistic similarity scores of pairs of reactions in the dataset assessed against those in the background (Dataset vs. Background, AUC = 0.76), for pairs in the filtered dataset assessed against those in the background (Filtered Dataset vs. Background, AUC = 0.81), for an ideal classification method with no false positives and false negatives (Ideal, AUC = 1.00), and for a non-discriminating classification method (Random, AUC = 0.50). Selected mechanistic similarity scores are shown within the curves.

To further explore the basis of reaction similarity of functionally analogous enzyme pairs, we investigated the extent to which similarity of overall reaction entails similarity of reaction mechanism. Table S3 provides values for the mechanistic similarity for all 95 pairs of functionally analogous enzymes in the dataset, as well as the alignments of reaction steps for each pair. Figure 3A shows the distribution of mechanistic similarity scores in the background dataset, in the functionally analogous dataset, and in the pairs of functional analogs with significant overall reaction similarity (“filtered dataset”). Figure 3B plots F-measures and significance levels for all possible similarity scores. Figure 3C shows a ROC curve for the mechanistic similarity of pairs of reactions in the dataset assessed against those in the background dataset (Dataset vs. Background), and for the filtered dataset assessed against those in the background dataset (Filtered Dataset vs. Background). The score where the F-measure was maximized, which coincides for both the dataset and the filtered dataset (Figure 3B), retrieved 11 (11.6%) of the pairs in the dataset and 10 (23.8%) of the pairs in the filtered dataset as similar, but only 0.95% of those in the background dataset. The Tanimoto coefficient at this point was 0.3793 and the enrichment factor was 12.16 for the dataset and 25.00 for the filtered dataset. Similar to the treatment of overall reactions, the pairs with mechanistic similarity scores equal or higher than this cutoff were called highly similar. The less stringent cutoff score for mechanistic similarity, i.e. including all pairs with similarity significant at the 5% level, was 0.2537. Using this second cutoff, the true positive rate increased to 20.0% (19 pairs) for the dataset and 33.3% (14 pairs) for the filtered dataset, and the enrichment factor was 4.25 and 7.08 for the dataset and filtered dataset, respectively. The pairs of enzymes with mechanistic similarity significant at the 5% level but below the F-measure optimized cutoff were called distantly similar. In general, functionally analogous pairs of enzymes, as defined by identical first three numbers of their EC codes, were more mechanistically similar than expected from the distribution of mechanistic similarity in the background, with almost half (14 of 29) of the EC sub-subclasses in the dataset containing at least one example of convergence of mechanism (Table 1). In total, 20% (19 of 95) of pairs shared significant mechanistic similarity, and this proportion increased to 33% (14 of 42) for pairs in the filtered dataset. Thus, at least as represented in the limited dataset available for this study, nature often converges to the same mechanistic solution when solving a related chemical problem, even when the starting templates belong to completely different structural scaffolds.

Two new features implemented in our algorithm for this study allowed us to consider reversibility of enzyme reactions and circular permutations of the steps in the reaction sequences (see Methods). Circularly permuting the steps of at least one of the reactions in a pair increased the similarity scores in 47 of the 95 pairs in the dataset. After inspection of all the permutations, we identified nine reaction mechanisms (M0010, M0043, M0047, M0049, M0055, M0093, M0097, M0098, M0216) where proton transfers at the beginning or end of the reaction sequence could be permuted to the end or beginning, respectively, without altering the outcome of the catalytic reaction. We therefore allowed the permutation of steps in these nine reaction mechanisms in our calculations, which in total accounted for improvements in the mechanistic similarity in 21 of the 47 possible pairs identified. Reversing the mechanism of one of the reactions in a pair increased the mechanistic similarity for 23 pairs, and in a further 28 pairs the same mechanistic similarity was obtained for both the alignment where the mechanisms were in the direction presented in MACiE and for the alignment where the direction of one of the mechanisms was reversed. Overall, the mechanistic similarity for 93 of the 95 pairs in the dataset was maximized when the mechanisms were aligned in the same direction that maximized overall reaction similarity. The two pairs that did not follow this rule (M0022 and M0077, M0054 and M0073) showed neither significant mechanistic similarity nor significant overall reaction similarity. After considering reversibility of enzyme reactions and circular permutation of steps, our classification of four pairs of enzymes (M0007 and M0093, M0008 and M0091, M0029 and M0098, and M0092 and M0093) went from mechanistically non-similar to similar.

### Similar and Non-Similar Catalytic Mechanisms

Of the 19 pairs with significant mechanistic similarity, none were identical in all steps, but for ten pairs the bond changes in all steps of one reaction were identical to or a subset of the bond changes in the steps of the other reaction. The remaining 76 pairs in the dataset had mechanistic similarity scores below the 5% significance level, and spanned 22 of the 29 EC sub-subclasses considered (Table 1). At the EC class level, more than half of the pairs of hydrolases (EC 3) had significant mechanistic similarities, whereas none of the pairs of the isomerases (EC 5) did. Looking at sub-subclasses with more than one pair in the dataset, we found that the carboxylic-ester hydrolases (EC 3.1.1) are the only sub-subclass where all possible pairs of mechanisms were similar. In contrast, there were several sub-subclasses with more than one non-similar pair. The two most striking examples were acyltransferases transferring groups other than aminoacyl groups (EC 2.3.1) and carbon-oxygen lyases acting on phosphates (EC 4.2.3). For each of these two sub-subclasses, none of the six possible pairs of reactions shared significant mechanistic similarity. The dataset had only two pairs of enzymes with identical four number EC codes, corresponding to beta-lactamase {Class A} (MACiE M0002, EC 3.5.2.6, PDB 1btl) [48]–[51], and beta-lactamase {Class B} (MACiE M0016, EC 3.5.2.6, PDB 1bc2) [52]; and 3-dehydroquinate dehydratase {type I} (MACiE M0054, EC 4.2.1.10, PDB 1qfe) [53], and 3-dehydroquinate dehydratase {type II} (MACiE M0055, EC 4.2.1.10, PDB 1gu1) [54],[55]. Both pairs of enzymes had identical overall reactions, but only the former pair (beta-lactamases) had significant mechanistic similarity. It has been reported in the literature that type I and type II dehydroquinases catalyze the same chemical reaction but by completely different mechanisms [53],[56]. Our algorithm correctly aligns the C-O bond cleavage common to the sub-subclass, which is catalyzed in the sixth step of M0054 and in the second step of M0055 (Table S3). However, type I dehydroquinase catalyzes the reaction in nine steps, whereas type II does so in only three. This difference in the number of steps severely diminished the mechanistic similarity calculated for the pair, and thus they are classified as non-similar by our metric.

As exemplified above, and in analogy to the dissimilarity in the numbers of bond changes found for non-similar overall reactions, sub-subclasses containing non-similar mechanisms included reactions with dissimilar numbers of steps (Table S5). Therefore, some of the mechanisms of enzymes in the 22 sub-subclasses with mechanistically non-similar pairs were embellished with extra steps, and this could explain in part the low mechanistic similarities calculated by our method. To avoid penalizing pairs of reactions with disparate numbers of steps, each mechanistic similarity score was normalized by the maximum possible similarity that could have been calculated given the number of steps in the mechanisms compared (see Methods). The cutoff at the 5% significance level for the normalized mechanistic similarity was 0.4583; 15 pairs had a score equal to or higher than this cutoff. Nine of these 15 pairs were considered similar according to the original (not normalized) measure of mechanistic similarity. Thus, six new pairs scored highly for mechanistic similarity after normalization (including the 3-dehydroquinate dehydratases referred to above), while ten of the 19 pairs that were mechanistically similar according to the original measure of similarity were non-similar according to the normalized mechanistic similarity. Thus, additional steps in some reaction mechanisms play a crucial role in their being designated as mechanistically dissimilar according to our measure.

### Mechanistic *vs.* Overall Reaction Similarity

In general, pairs of enzymes from EC sub-subclasses containing similar overall reactions were more likely to share mechanistic similarity than those from EC sub-subclasses including non-similar overall reactions (Table S4). This observation is also in agreement with the ROC curves for the dataset and filtered dataset in Figure 3C, the latter of which outperforms the former. Of the 19 pairs of reactions that presented significant mechanistic similarity, 12 had highly similar overall reactions, two had distantly similar overall reactions, and five had non-similar overall reactions.

**Figure 4:**
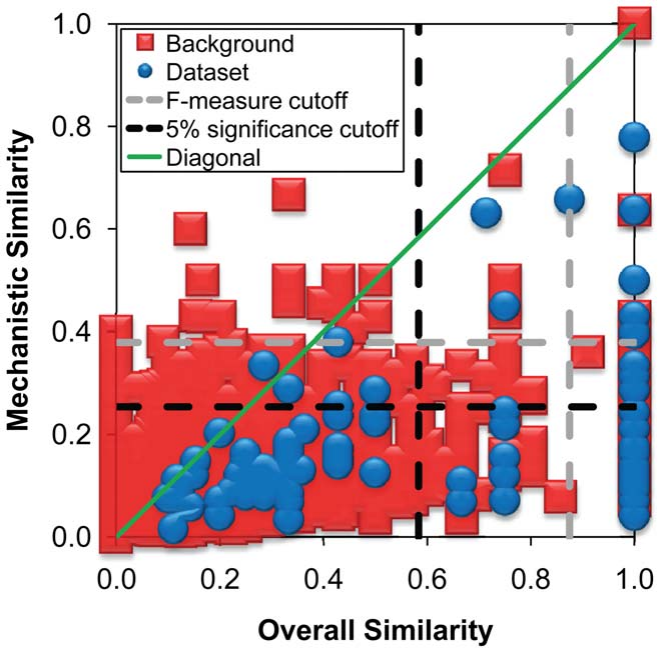
Mechanistic *vs.* overall reaction similarity. All 95 pairs in the dataset of functional analogs and 3570 pairs in the background dataset are included. Sizes of shapes are not proportional to the number of pairs they contain.

Figure 4 plots mechanistic similarity against overall reaction similarity for all 95 pairs of functionally analogous enzymes and for all 3570 pairs in the background dataset. Pairs in the background dataset populated all areas of the plot, whereas pairs in the functionally analogous dataset almost exclusively populated the area below the diagonal. Only one pair from the dataset appeared above the diagonal in Figure 4: phosphoenolpyruvate carboxykinase (ATP) (MACiE M0051, EC 4.1.1.49, PDB 1aq2) [57] and methylmalonyl-CoA decarboxylase (MACiE M0070, EC 4.1.1.41, PDB 1ef8) [58]. These enzymes share an identical step and highly similar mechanisms, yet the extra phosphorylation catalyzed by the former enzyme makes the overall reactions non-similar (Table S3). Thus, for functionally analogous pairs of enzymes, overall reaction similarity serves as an upper bound on mechanistic similarity. This is expected since functionally analogous enzymes are proteins without a common ancestor that have converged to catalyze a similar overall reaction. In contrast, in homologous superfamilies of enzymes, conserved active site residues, organic cofactors or metal ions catalyze at least one identical catalytic step, even in very different overall reactions [4]–[7], [19]–[21]. Thus, homologous superfamilies exhibit significant mechanistic similarity, but not necessarily significant overall reaction similarity [59]. This notwithstanding, the higher the overall reaction similarity attained by a pair of functionally analogous enzymes, the higher the chances are that the pair also shows mechanistic similarity. Considering both functionally analogous and homologous enzymes, it seems that overall reaction similarity can vary greatly after sequence divergence, with mechanistic similarity being much more conserved in homologous superfamilies. A companion study to this one is currently under way to compare in detail overall reaction and mechanistic similarities in homologous superfamilies.

### Identical Mechanistic Steps

As mentioned above, there is evidence of conservation of at least one common fundamental mechanistic step among all members in several homologous superfamilies of enzymes [4]–[6]. This prompted us to investigate whether functional analogs have converged to share at least one catalytic step as well. First, the Smith-Waterman algorithm [40] was used to search for all possible identical steps between pairs of reactions in forward and reverse alignments of mechanistic steps. Sixty-six identical paired steps were identified in 22 pairs of reactions. Then, we looked at how many of these identical steps were present in the global alignments that maximize mechanistic similarity between pairs of reactions. Thirty-one of the 66 identical paired steps were identified in 21 of the total 22 pairs of reactions (Table 1 and Table S3). In all 21 pairs, the identical step(s) included bond changes common to the overall reactions catalyzed. Instead, none of the bond changes in the identical step in the reactions where the step was not included in the best alignment (M0030 and M0077) were common to the overall transformation catalyzed by the enzymes. In general, the 31 pairs of identical steps contained from two to eight bond changes, and were present in enzymes from 12 of the 29 EC sub-subclasses in the dataset, spanning all EC classes except the isomerases (EC 5). The oxidoreductases contained the highest proportion of pairs with an identical catalytic step (three out of five pairs). In terms of EC subclasses, the hydrolases acting on ester bonds (EC 3.1) had the highest proportion of pairs with identical steps (four out of eight pairs). Six sub-subclasses contained only pairs with at least one identical mechanistic step. Four of these sub-subclasses contained only one pair of reactions, and two contained three pairs each: the oxidoreductases acting on the CH-OH group of donors with NAD+ or NADP+ as acceptor (EC 1.1.1), and the carboxylic-ester hydrolases (EC 3.1.1).

**Figure 5:**
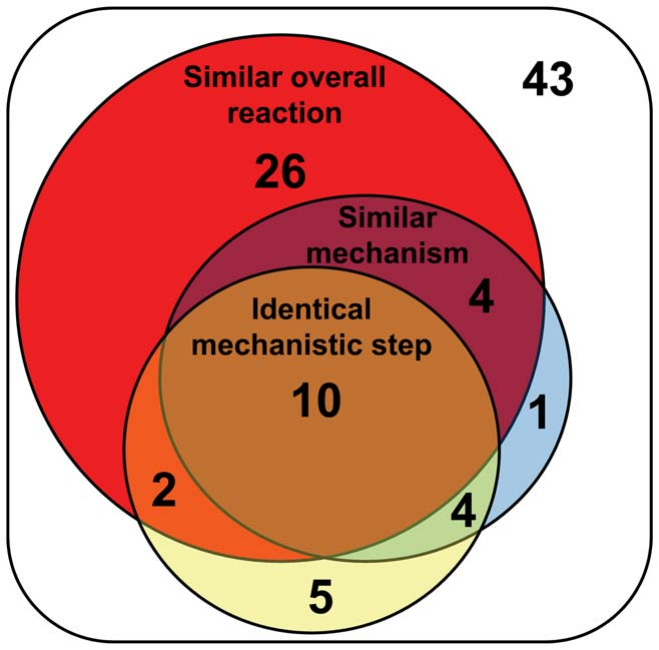
Venn diagram showing combinations of similarity of overall reaction and mechanism, and identical mechanistic steps for pairs of enzymes in the dataset. Sizes of shapes are not proportional to the number of pairs they contain.

Identical mechanistic steps were more likely to occur in pairs of enzymes from EC sub-subclasses containing similar overall reactions (Table S4). Because there is a direct correlation between overall reaction and mechanistic similarity, it is unsurprising that identical mechanistic steps were also more likely to occur in pairs of enzymes from EC sub-subclasses containing similar mechanisms (Table S5). Figure 5 shows a Venn diagram summarizing all possible combinations of overall reaction similarity, mechanistic similarity and identical catalytic steps for the pairs of enzymes in the dataset of functional analogs. Twelve of the 21 identical steps were found in pairs with similar overall reactions, and of those, most (10 of 12 identical steps) were also included in pairs of enzymes with significantly similar mechanisms. An example of convergence to the same overall reaction (Figure S2), together with convergence of mechanisms and identical mechanistic steps (Figure S4) is offered by the carboxylic-ester hydrolases (EC 3.1.1), represented in the dataset by phospholipase A2 (MACiE M0083, EC 3.1.1.4, PDB 1l8s) [60],[61], 1-alkyl-2-acetylglycerophosphocholine esterase (MACiE M0094, EC 3.1.1.47, PDB 1bwp) [62],[63], and triacylglycerol lipase (MACiE M0218, EC 3.1.1.3, PDB 1hpl) [64]–[67].

### Similarity of Active Sites: Residues, Cofactors and Metals

Gherardini and colleagues recently reported [11] on the structural convergence of sets of catalytic residues in functionally analogous enzymes in the Catalytic Site Atlas (CSA) [26]. Because the dataset in MACiE originated from the catalytic residue dataset of Bartlett and colleagues [68] and the CSA, the results presented in this prior work can be extended to the dataset studied here (see Methods). For fifteen of the enzymes in our dataset of functional analogs, the same enzyme or a homolog was present in the set of convergently evolved active sites identified by Gherardini *et al.* [11]. These enzymes span nine of the 29 sub-subclasses in the dataset, with three sub-subclasses presenting one or more pairs (Table S6).

Of the four pairs presenting structurally equivalent active site residues in the dataset, the two pairs of hydrolases had similar mechanisms. Phospholipase A2 and 1-alkyl-2-acetylglycerophosphocholine esterase have already been introduced in the previous section in the context of identical mechanistic steps in the carboxylic-ester hydrolases (Figure S4). The other pair is that of N-carbamoylsarcosine amidase (MACiE M0025, EC 3.5.1.59, PDB 1nba) [69],[70] and glutamin-(asparaginase) (MACiE M0029, EC 3.5.1.38, PDB 1djo) [71]. A convergently evolved catalytic triad in the former enzyme is responsible for nucleophilic attack by a cysteine residue on the substrate and for activation of a water molecule that subsequently displaces the covalently attached cysteine. In the latter enzyme the triad is instead only responsible for activation of the water molecule, while an additional triad is responsible for the nucleophilic attack (by threonine) on the substrate. This pair of reactions was classified by our method as highly similar in terms of both overall reaction and mechanism, and two steps in these reactions also involve identical bond changes. In contrast, neither of the two pairs of acyltransferases showed mechanistic similarity. The equivalent histidine residue in aralkylamine N-acetyltransferase (MACiE M0022, EC 2.3.1.87, PDB 1b6b) [72] and acyl-[acyl-carrier-protein]-UDP-N-acetylglucosamine O-acyltransferase (MACiE M0069, EC 2.3.1.129, PDB 1lxa) [73],[74] acts as a general base in both enzymes, but additional residues in the former enzyme play additional functional roles not present in the latter. The equivalent pair of cysteine residues in formate C-acetyltransferase (MACiE M0030, EC 2.3.1.54, PDB 2pfl) [75]–[77] and acetyl-CoA-acyltransferase (MACiE M0077, EC 2.3.1.16, PDB 1afw) [78],[79] undergo reactions that proceed only through homolytic chemistry in the former enzyme, but exclusively through heterolytic chemistry in the latter. In accordance with this last case, the homologs of formate C-acetyltransferase and acetyl-CoA-acyltransferase present in the work by Gherardini and colleagues were used by the authors to exemplify the scenario where two unrelated enzymes have convergently evolved active sites but approach the reactions with different chemical detail [11].

In addition to comparing structural similarities (as defined by [11]) in the set of catalytic residues used by the functional analogs, we also compared the usage of metal ions and organic cofactors, which extend the repertoire of enzyme catalysis by allowing exploration of chemical space that is not possible using the canonical amino acid residues [28],[47]. In total, in 31 of the 95 pairs of functional analogs both enzymes in a pair do not use organic cofactors or metal ions, and in a further two pairs, both enzymes use an identical stoichiometric number and type of metal ions. For the remaining 62 pairs, the cofactors and metal ions used by one functional analog are different from those used by the other. These pairs can be divided into four groups:

- In 41 pairs one of the functional analogs uses metal ions, whereas the other functional analog does not.
- In eight pairs both enzymes utilize different types and/or stoichiometric numbers of metal ions.
- In four pairs one enzyme uses an organic cofactor, whereas the other does not.
- In nine pairs one enzyme in the pair uses metal ions and organic cofactors, while the other enzyme uses metals only (three cases), cofactors only (one case), or none (five cases).

Previous studies have implied that convergence of active sites entails mechanistic convergence [80], yet it has been shown before [11] and further quantified here that this is not always the case. Particular active site residues, or groups of residues (dyads and triads), do indeed relate to particular mechanistic steps. However, this does not ensure that the remaining steps in a mechanism are similar, or that the type of reaction chemistries used are identical. In addition, because we have defined mechanistic similarity based on bond changes for which only the atoms in catalytic species (amino acid residues, organic cofactors and metal ions) directly involved in the bond changes are considered, pairs of mechanistically similar enzymes are allowed to present either the same or different catalytic species. The results indicate that mechanistic analogs have converged to perform similar catalytic steps, sometimes with highly similar active sites as in the cases studied by Gherardini et al. [11], but more often, as shown in this study, with different active sites. Thus, in order to perform similar mechanisms, functionally analogous catalytic species do not have to be of the same type, or be located in a similar 3D environment.

## Discussion

Large-scale studies of convergence of function in enzymes have been relatively rare despite their potential value for many applications. In this article, we assessed, for a set of 95 pairs of enzymes defined by the EC classification system as functional analogs, the similarity in bond changes of both their overall reactions and the steps of their mechanisms. Although EC numbers have been used historically in classification of function in enzymes, our results indicate that, at least for the limited set of non-homologous enzymes analyzed here, over half (56%) of the pairs of enzymes fail to show statistically significant similarities in their overall reactions. However, of those that do show overall reaction similarity, one third also showed statistically significant mechanistic similarity. In the following sections, the implications of these results are discussed along with suggestions for improved discrimination in the functional classification and annotation of functionally analogous enzymes and for the selection of appropriate starting scaffolds for enzyme engineering. The limitations of this study and some concluding remarks are also presented.

### Classification of Enzyme Reactions

The EC system [1] has long been recognized as the gold-standard for the classification of enzyme reactions with the third level (EC sub-subclass) considered a description of the reaction chemistry. In this work, we found, however, that only 44% of the reaction pairs in the dataset shared sufficient bond changes in their overall reactions to make them significantly similar. The lack of similarity at the sub-subclass level of the EC is global, spanning 12 of the 29 sub-subclasses in the dataset. This suggests that in general more stringent criteria may be required for classification of the reactions in EC sub-subclasses so that each designates only those functional analogs with quantitatively similar overall reactions. We note, however, that the method used to measure overall reaction similarity is based on the identification of identical bond changes in the overall reactions and mechanisms of pairs of enzymes, a rather conservative approach that may miss more remote similarities (see Limitations of the Study).

Over the past decade, the Gene Ontology (GO) has been developed to describe three domains of molecular and cellular biology, including molecular function [81],[82], and to allow large-scale quantitative comparisons of similarities among terms within each domain, making it relevant to this discussion as well. For the catalytic activities of enzymes, the GO is largely based on the EC classification [Text S1], potentially supporting measurement of functional similarity among enzymes by measuring similarity between GO terms. Indeed, several methods have been reported that measure the similarity between GO terms, including one based on the minimum distance between distal GO IDs [83], another using an F-score to measure distance between paths on probabilistic GO trees [84], and a third based on an “information content” approach [85]. However, all of these methods accept as an underlying assumption that the EC and GO classifications correctly distinguish similar overall reactions from dissimilar ones. Our results contradict that assumption for a large proportion of pairs of overall reactions in our dataset (56%), suggesting that these measurements may not accurately reflect functional similarities and dissimilarities among the actual reactions catalyzed by enzymes.

Besides the work reported here, others have described approaches that explicitly take into consideration the overall reactions of enzymes [45], [46], [86]–[90]. Kotera, Yamanishi and colleagues [45],[46] mapped chemical structure changes in substrate-product pairs to EC numbers. Structural changes were described automatically with Reaction Classification (RC) numbers by aligning substrate-product pairs and defining the reaction center, matching ligand structures and those atoms that differ in the ligand structures. Using Jack-knife cross-validation, they predicted EC sub-subclasses with high recall and precision for reactions covering most of the sub-subclasses in their dataset [46]. However, they also showed that EC sub-subclasses related to an average of about 15 different RC combinations and that some RC combinations corresponded to more than one EC sub-subclass, leading them to conclude that there was room for improvement in the consistency of the EC system [45]. Gasteiger, Sacher and colleagues [86],[87] calculated six physicochemical descriptors for reacting bonds on substrates to capture all major electronic effects influencing the reaction. They chose hydrolases for their studies, as the sub-grouping of this class of enzymes in the EC classification is based on the type of bond that is hydrolyzed, and thus chemically meaningful. Using Kohonen self-organizing maps, they found that classification of reactions based on physicochemical effects largely corresponds to the EC classification for this class of enzymes [86],[87]; their similarity algorithms also reveal finer details, leading them also to suggest that the EC classification could be improved [87]. Finally, Latino and colleagues [88]–[90] used topological and physicochemical descriptors to encode substrates and products of reactions. Structural changes were then represented by the difference between the descriptors of products and substrates. Also using Kohonen self-organizing maps, they observed that most EC sub-subclasses clustered together, but that there are examples of similarity not revealed by the EC as well as cases of problematic classifications [88]. By using Random Forest classifiers, they also predicted the sub-subclass of EC reactions with accuracies up to 85% [90].

An ultimate goal of our work is to develop a classification system based on mechanistic similarity instead of overall reaction similarity. However, the time lag between overall reaction determination and mechanistic studies, and the few mechanistic studies available compared to the number of enzymatic reactions that are known, means that classifications based solely on overall reactions are still needed. The overall reaction similarities calculated here could thus be useful for refining definitions of EC sub-subclasses so that only significantly similar reactions are grouped together. An automated method like that of Kotera, Yamanishi and coworkers could be used to describe bond changes of overall reactions. Then, as proposed by our method, Tanimoto coefficient similarities above a certain cutoff could be used to generate functionally analogous groups. The generation of smaller, more tightly defined groups of related reactions may help reduce the apparently high rate of convergence of function seen across species and in the sequence and structure databases [2], [8]–[11],[14],[15],[17], which may in part result from an insufficiently stringent classification of overall reactions at the sub-subclass level.

### Prediction and Annotation of Enzyme Function

Most current methods of function prediction use sequence and structure relationships as the basis for functional inference [91]. The analyses presented here and those of others suggest the widespread existence of functional analogy [2], [8]–[11],[14],[15],[17], indicating that methods other than homology must be used to annotate convergently evolved enzymes. We are thus obliged to use orthogonal approaches to function prediction, many of which use biological information, including operon context [92],[93], gene fusion [94], and phylogenetic profiles [95],[96]. Especially relevant to the issues addressed in this work, Hermann and colleagues have recently reported the docking of high-energy intermediates to enzyme structures [97], describing how this procedure led to prediction of the function of an enzyme of previously unknown activity [98]. Because this procedure exploits transition state recognition in enzymes by docking transition-state-like conformations (high-energy intermediates) instead of the ground-state structures of substrates, it provides evidence about the possible mechanism of catalysis. However, high-energy intermediates must be generated *a priori*, and initial knowledge about the possible function of an enzyme is a requirement for this step.

Because our results show that functionally analogous enzymes can have key mechanistic steps in common, it can therefore be expected that docking high-energy intermediates (deduced from known reaction mechanisms) into newly determined or predicted structures could also be useful for function prediction of analogous enzymes, not just those identified as homologous to enzymes with known mechanisms. Furthermore, the mechanistic similarity measures presented in our previous article [37] and developed further here could contribute to the identification of appropriate high-energy intermediates for *in silico* docking, and for searching for similarities between these intermediates and those catalogued by MACiE and SFLD. Although there are currently many more sequences and structures than known mechanisms for enzymes, these and other publicly available enzyme databases that include catalytic mechanisms are growing. As these increase in coverage, we envision that orthogonal approaches to function prediction such as docking high-energy intermediates, aided by mechanistic information and algorithms to measure reaction similarity, will improve on the success of current homology-based methods. Even if sequences or structures cannot be annotated with full mechanisms, it may still be possible to annotate them with information on individual catalytic steps, helping to guide future experimental verification.

### Enzyme Engineering

The abundance of cases of functional convergence demonstrates the ability of nature to reach the same solution to a chemical problem from different starting structures and active site architectures. Our results also add support to the conclusion from previous studies that chemistry has been more conserved than substrate specificity during evolution [4]–[7], [19]–[21]. In other words, the chemistry catalyzed by enzymes is less prone to changes than is substrate recognition. Thus, in order to engineer a new enzyme with new catalytic properties, the best starting template would likely be an extant enzyme that catalyzes a reaction with high mechanistic similarity to the new desired activity [99]. Our methods for measuring mechanistic similarity can be used to identify such templates. Global alignment techniques can support searches for a template enzyme showing high mechanistic similarity to those of a desired new reaction, perhaps even for multiple steps. Alternatively, local alignment searches against a database of enzyme mechanisms can be used to identify known enzyme structures and active sites capable of catalyzing the different individual steps of a target reaction. Then, a consensus active site could be created by combining elements from different enzymes, in analogy to the procedure adopted by Jiang and colleagues for designing retro-aldol enzymes [100].

In support of these possibilities, the MACiE database is already searchable by mechanistic bond changes. The SFLD implements structure and substructure searches of reaction mechanisms via SMILES/SMARTS strings and a reaction drawing application. Further work is under way in our laboratories to offer a web server implementing the mechanistic and overall reaction similarity algorithms presented here. The server will allow users to search for similar overall reactions and reaction mechanisms, as well as perform global and local alignments of mechanistic steps with reaction mechanisms currently present in the MACiE and SFLD databases.

### Limitations of the Study

There are several caveats that should be considered when assessing the results and observations presented here. First, the size of the dataset used in this study is small compared to known examples of convergence of function present in nature. The big limitation in the size of the dataset arises from the lack of mechanistic information available for enzymes, which we obtain from the MACiE database. MACiE only includes enzymes with structures available in the PDB [36] and for which there is sufficient evidence for a mechanism in the primary literature. Thus, the enzymes present in MACiE are a subset of those that have been amenable to both structural and mechanistic studies, limiting the extent to which the findings of this study can currently be generalized to other convergently evolved enzymes. Furthermore, overall reaction and mechanistic similarity in this work are based upon the descriptions of reactions reported in MACiE, thus, the results presented here are only as good as the reaction data contained therein. Of particular interest is that when there are alternative reaction mechanisms proposed for an enzyme reaction, only one of the possible reaction sequences is represented in MACiE. Specifically, 27 of the 223 reactions from the version of MACiE we used in this study are annotated as having alternative mechanistic steps for one or several of the steps of the canonical reaction. Of these 27 reactions, three were included in the dataset of functional analogs (M0002, M0007 and M0222). Additionally, there are cases where similar mechanisms in different reactions differ in the number of steps the authors assign to them, and there are mechanisms not cited as a stepwise reaction in the literature, for which step-by-step mechanisms had to be inferred (as is the case for 1-alkyl-2-acetylglycerophosphocholine esterase presented in Figure S4) [47]. Regardless of these caveats, MACiE is a valuable resource for enzyme catalytic mechanisms, and the only publicly available database suited for the type of analysis presented here. Despite the complications encountered in characterizing and storing detailed enzyme mechanisms and the uniformity of nomenclature needed for analyses such as ours, MACiE provided us with a dataset whose size seems remarkable, and of the same order of magnitude as datasets previously collected for analysis of divergence of function in proteins [7],[101]. The fact that all entries also have known structures allows easier discrimination between homologous and non-homologous pairs of enzymes. Nonetheless, a dataset of ninety-five pairs of enzymes is still small and caution is required in interpreting the results broadly.

Second, overall reaction and mechanistic similarity were obtained for this study using an entirely automated algorithm based on bond change information. While this approach allows for consistency in the way similarity is defined, it is not as well suited as a manual analysis, such as reported by Gherardini et al., [11] for discrimination of complex issues associated with the difficult problem of comparing functional characteristics. For example, the current algorithm disregards the evident similarities between related but not identical bond types, e.g. carboxylic-ester hydrolysis *vs.* phosphoric-ester hydrolysis. Nor does the algorithm differentiate between atom types for each element, i.e. a single bond between an aromatic carbon and chlorine is considered identical to a single bond between an aliphatic carbon and chlorine. Moreover, the algorithm does not take into consideration information regarding the catalytic species that support each mechanism, apart from the identity of atoms in species that are directly involved in the bond changes. Thus, in its current form, the method is very conservative and only defines pairs of overall reactions or mechanisms as similar where there is an obvious overlap between identical bond types. As a result, it misses those examples of similarity that do not fulfill this exquisite requirement, implemented in this preliminary study in part to avoid retrieving false positive similarity hits. Future developments of the algorithm could include more nuanced metrics, e.g., to identify similar reaction mechanisms occurring in related but not identical bond types or to differentiate atom types.

A third potential limitation for this study is its reliance on the CATH database [41] to define a gold-standard set of non-homologous enzymes. As with any other sequence/structure classification system, distant evolutionary relationships could have been missed and some of the examples analyzed here could represent cases of divergently evolved enzymes rather than convergently evolved ones. However, databases such as CATH (and SCOP [34]) represent the state of the art with respect to identification of evolutionary relationships in proteins.

These caveats must be taken into account in evaluating the extent to which our results can be generalized to the much larger set of convergently evolved enzymes that could not be included in this initial study. However, we note the breadth of the dataset with respect to its coverage of EC functional classes and of structural classes most frequently represented in enzymes. Thus, we suggest that this systematic and quantitative comparison of reaction similarities in functionally analogous enzymes raises legitimate questions for further development of reaction classifications. In particular, the large proportion of enzymes in the same EC sub-subclass found to be dissimilar by our metric in both overall reaction and mechanistic steps indeed suggests the potential value of refining the EC system so that overall reactions in the same sub-subclass share at least a sufficient number of bond changes to make them statistically more similar than random pairs of reactions. As larger and more comprehensive datasets become increasingly available, we expect that systematic studies using our metric or others ([11], for example) will elaborate these themes further.

### Conclusions

We investigated the relationship between functional similarity in enzymes according to the EC classification and similarity of overall reactions based on the bond changes that occur in the transformations from substrates to products. The dataset we compiled only represents a small portion of currently known cases of convergence of function and thus extrapolation of the results to a broader context should be interpreted with caution. Using our metric, less than half of the pairs of enzymes in our dataset showed significant overall reaction similarity, leading us to conclude that the EC classification, and by extension catalytic activities in the Gene Ontology, may not accurately reflect functional similarities among a large subset of the reactions catalyzed by enzymes. We also investigated the extent to which overall reaction similarity implies mechanistic similarity in functionally analogous enzymes and concluded that in contrast to results reported for studies of homologous enzyme superfamilies, overall reaction similarity serves as an upper bound for mechanistic similarity in functional analogs. Additionally, we found that one third of the pairs with similar overall reactions converged to similar catalytic mechanisms. The constraints imposed by similar overall reactions, as well as the limited chemical repertoire used in enzyme catalysis suggests that functionally analogous enzymes invoke similar strategies for transition state stabilization along their reaction pathways, often leading to mechanistic similarity, even in the absence of active site similarities.

It is difficult to assign in a systematic manner a relative value to the definitions of reaction similarity used by the EC, compared to those described in this work, for the many applications requiring explicit definitions of molecular function in enzymes. The EC system was created to capture major classes of chemical transformations at a time when reaction and mechanistic data were sparse and no quantitative measures of reaction similarity were available. Thus, the EC likely could benefit from a systematic and quantitative evaluation of its utility now that these data are becoming both more extensive and more accessible from MACiE and other databases. This study was designed to provide just such an evaluation, using as a metric similarity in bond changes, validated to the extent possible using a relevant background model. Ultimately, we expect that the foundation laid in this work will allow the generation of an improved classification for enzymes based on quantitative similarities of overall reaction and/or mechanistic information, thereby improving its usefulness for functional annotation and other applications and allowing it to serve as an additional resource for comparison of enzyme reactions.

## Methods

### Dataset

EC numbers, overall reactions and catalytic mechanisms for enzymes were obtained from version 2.3.9 of the MACiE database in ISIS/Base Reaction Data Format (RDF) [29],[30]. Functionally similar enzymes were identified as those that shared the first three levels of their Enzyme Commission numbers (identical EC sub-subclass) [1], so that at least the overall chemistry catalyzed by the enzymes is similar, regardless of their substrate specificity. Each group of functionally similar enzymes was then made non-homologous by randomly selecting only one protein among those with at least one identical domain in the same superfamily as defined in CATH version 3.2.0 [41]. At this stage, enzymes that had not yet been divided by CATH into domains, and chains that had been divided, but not yet assigned to superfamilies were removed.

Coverage of the dataset in terms of EC space was analyzed via downloading the list of EC numbers provided by ExplorEnz [42] as an XML data file on 29 September 2008. Coverage in terms of structural space was evaluated using version 3.2.0 of the CATH database [41]. PDBSprotEC [43] version of 24 September 2008 was used as the reference set of structures in the PDB with EC numbers assigned. The number of structural domains present in enzymes of known structure was obtained by assigning all PDB chains with EC number in PDBSprotEC to CATH superfamilies. To determine the minimum number of non-homologous domain combinations that map to each EC sub-subclass in PDBSprotEC, a two-step procedure was followed. First, the CATH database was parsed to assign the domains of all the enzymes catalyzing each EC sub-subclass in PDBSprotEC to superfamilies. Enzymes with chains not chopped or domains not assigned were not considered. Then, from each EC sub-subclass, we randomly selected enzymes and removed all other enzymes with any domains in the same CATH superfamily as the selected one, thus generating a non-homologous dataset. The random selection was repeated 500 times, provided the number of selections needed to find the best solution (x) can be approximated to −ln(p)/f, where p is the probability corresponding to the confidence level of having found the best solution and f is the fraction of all possible selections that produce the best solution. By setting p = f = 0.01, this formula gives x≈461. The number of domain combinations reported in Table 1 corresponds to the minimum number of combinations obtained from the 500 selections.

### Measuring Overall Reaction and Mechanistic Similarity

In MACiE, each catalytic mechanism is presented as a sequence of mechanistic steps, with substrates and products of each step representing probable energetic local minima on the reaction pathway. We recently developed two methods to quantify similarity between mechanisms of enzyme reactions [37]: one based on the bond changes (bonds formed, cleaved and changed in order) that occur in each step, and one based on a fingerprint that captures various aspects of each catalytic step. Here, the method based on bond change information was used. In brief, each mechanistic step was represented as the set of bond changes occurring in the transformation from substrate(s) to product(s) in that step. Then, similarity between sets of bond changes for each possible combination of steps between two reactions was computed using Tanimoto coefficients [38] and stored in a similarity matrix. Based on this similarity matrix, the Needleman-Wunsch algorithm [44] was used to obtain the best global alignment between steps. Gap openings and gap extensions are not penalized in the alignments, since we don't know how the similarity between reaction mechanisms has arisen and are thus unable to assess whether insertion or deletion of steps in a reaction sequence should or should not be penalized. Finally, to obtain the “mechanistic similarity” between sequences of steps, a new Tanimoto coefficient was computed using the number of steps in each reaction and the Needleman-Wunsch similarity as inputs (Figure 1).

For the present work four new features were added to the algorithm:

1. First, we were interested in measuring similarity of overall reactions. For this purpose Tanimoto coefficients were computed using the set of bond changes occurring in the transformation of the substrate(s) to product(s) of the reactions catalyzed (“overall similarity”) (Figure 1).
2. Secondly, the Smith-Waterman algorithm [40] was implemented for finding local alignments between mechanistic steps of reaction sequences.
3. Thirdly, reversibility of enzyme reactions was considered. The direction in which the reactions occur *in vivo* is not specified by the EC system. Instead, all reactions in a given class are stored in a common direction, even if the direction has not been demonstrated for all enzymes [1] or if the reaction has only been observed in the reverse direction [102]. By contrast, in MACiE, reactions are entered in the direction in which they are reported in the source literature, which often corresponds to the *in vivo* direction, not necessarily the common direction defined by the EC. Unlike amino acid sequences, enzyme catalytic reactions are by definition reversible and therefore inverse similarity [103]–[107] has to be taken into account. Reversibility of enzyme reactions was considered explicitly for both overall reaction similarity and mechanistic similarity. This was done by inverting the bond changes in each set of bonds (e.g. a formation of a C-O bond was inverted to a cleavage of a C-O bond) and by reversing the order of the steps in the reaction sequences. Each pair of overall reactions and reaction mechanisms were compared in the direction in which they appear in the MACiE database (forward direction), and then we reversed the bond changes of one overall reaction and reaction mechanism of the pair (reverse direction) and compared it against the bond changes of the other reaction in the forward direction. The values reported for overall reaction similarity and mechanistic similarity correspond to the highest value obtained between the two possible directions. In principle, prior to measuring mechanistic similarity, it would have sufficed to orient the reactions in the common direction defined by the EC classification, or the direction that maximizes overall reaction similarity (which only for pair M0031–M0046 turned out to be different to the common direction defined by the EC). However, the principle of microscopic reversibility of enzymatic reactions implies that the transition states that an enzyme stabilizes in converting substrates to products are identical to those it stabilizes when catalyzing the reverse reaction [108]. Thus, it might be possible for two enzyme reactions to have high overall reaction similarity when oriented in the common direction assigned by the EC classification or in the direction that maximizes overall reaction similarity, but still have high mechanistic similarity when one of the mechanisms is in the opposite direction.
4. Fourthly, circular permutations of the steps in the reaction sequences were considered. All possible circular permutations of both reaction sequences being compared were generated, and then all possible combinations between circular permutations of steps were used to search for mechanistic similarities. Only those permutations that generated mechanistic similarity scores higher than those obtained for the original reaction sequences present in MACiE were manually inspected. Those circular permutations of steps from/to the beginning to/from the end of reaction sequences that involved simple proton transfers and that did not otherwise alter the outcome of catalysis were only accepted.

In MACiE, 8% of enzyme reactions include steps that either spontaneously form the enzyme's substrate from the starting materials of the overall reaction proposed by the EC classification, or spontaneously form the products of the EC reaction from an intermediate generated by the enzyme [47]. The inclusion of the bond changes in these steps obscures the real similarity between overall reactions and mechanisms of enzymes and therefore they were not considered for this study [37],[47]. That is, for reactions containing spontaneous steps, these steps were removed when measuring mechanistic similarity. Additionally, overall reactions were re-annotated, so that they did not include the bond changes that occurred in the spontaneous steps. Furthermore, in the overall reactions in MACiE, in addition to bonds formed, cleaved and changed in order, there also exists a fourth type of bond change called bonds involved, defined as bonds that change stereochemistry during the course of an overall reaction. Because these changes in stereochemistry are always the result of combinations of the other three types of bond changes, all bonds that change stereochemistry were removed from the overall reactions, and replaced with bond formations, bond cleavages and bond order changes wherever appropriate.

Because we found that reactions with non-similar overall reactions were dissimilar in their number of bond changes, and that reactions with non-similar mechanisms were dissimilar in their number of mechanistic steps, each Tanimoto coefficient was normalized by the maximum similarity that could be obtained given the reactions compared. For overall reaction similarity, each similarity score was divided by the maximum Tanimoto coefficient that could be obtained comparing the reactions, assuming that at least all bonds in the reaction with the least bond changes are identical to bonds in the other reaction. For example, if two overall reactions containing three and five bond changes are compared, the maximum similarity that could be obtained between them is 0.6000 (3/[3+5−3]), and the Tanimoto coefficient obtained for the similarity between their overall reactions would be divided by this fraction to obtain the normalized similarity. Likewise, for mechanistic similarity, each Tanimoto coefficient was divided by the maximum score that could be obtained, assuming that all steps in the reaction with the least steps are identical to steps in the other reaction. Table S3 provides values for overall reaction and mechanistic similarity before and after normalization.

The set of bond changes for every mechanistic step and overall reaction used in this work were included in Table S7 in the online supporting information. Overall reactions from the online version of MACiE can be accessed directly at URLs of the form: http://www.ebi.ac.uk/thornton-srv/databases/cgi-bin/MACiE/getPage.pl?id=M0001. Mechanistic steps from the online version of MACiE can be accessed directly at URLs of the form: http://www.ebi.ac.uk/thornton-srv/databases/cgi-bin/MACiE/getPage.pl?id=M0001.stg01.

### Assessing Significance

To evaluate the statistical significance of similarity scores, the results obtained for the dataset of functionally analogous enzymes were compared with those for a background dataset. To compose the background dataset, all enzymes from MACiE version 2.3.9, excluding the 80 enzymes from the dataset of functional analogs, were considered. From these 143 enzymes, all those with chains not chopped by CATH, those with chains chopped but with domains not assigned to a superfamily, and those without bond changes in their overall reactions were removed. MACiE entry M0201 was also removed, since structural information is only known for one of its two subunits. The remaining 120 enzymes were filtered to exclude both functional analogs and structural homologs. This was achieved by randomly selecting proteins from the pool of 120 enzymes and removing all other enzymes in the same EC sub-subclass as the selected one (functional analogs) or with at least one domain in the same CATH superfamily (structural homologs). Enzymes continued to be selected at random from the pool until no enzymes were left.

Pairs in the dataset were considered as positive hits, i.e. those above a given cutoff similarity score were considered true positives (tp), and those below false negatives (fn). Concordantly, pairs in the background dataset were considered as negative hits, i.e. those above a cutoff similarity score were considered false positives (fp), and those below true negatives (tn). At every possible value for the similarity of overall reactions and catalytic mechanisms tp, tn, fp and fn, were computed and stored in confusion matrices. In order to obtain an objective cutoff score to best separate hits in the dataset from those that occur in a random distribution (background dataset), we selected the score that maximizes the F-measure, the harmonic mean of precision and recall, defined as (2×Precision×Recall)/(Precision+Recall), where Precision is defined as tp/(tp+fp), and Recall as tp/(tp+fn).

The F-measure has been previously shown to be a good compromise between sensitivity and specificity [109]. Furthermore, the scores obtained maximizing the F-measure for both overall reaction and mechanistic similarity in the dataset were identical to those obtained when maximizing the Matthews Correlation Coefficient (data not shown), which has also been proposed as a method for optimizing a cutoff score for the partition between positive hits and hits in a background distribution [110]. Because the cutoff that maximized the F-measure was very stringent, significance at the 5% level was also considered as an additional threshold score. The similarity of enzyme pairs from the dataset is said to be significant at the 5% level when less than 5% of pairs of enzymes in the background dataset have an equal or higher Tanimoto coefficient. Areas under the Receiver Operating Characteristic curves (AUC) cited in the figures were calculated using the trapezium rule.

### Similarity of Active Sites

Gherardini and colleagues have recently identified structural matches between active site residues of functionally analogous enzymes [11]. In brief, the mean position of the side-chain centroids of all catalytic residues described in the Catalytic Site Atlas (CSA) were used to define an active site including all catalytic residues and all those residues whose side chain centroids were within 7.5Å of the catalytic ones. Query3D [35] was then used to identify the largest subset of identical residues in a pair of active sites that can be superimposed under an RMSD threshold of 1.7Å. Just functionally analogous pairs of enzymes were analyzed and only matches that comprised at least one pair of superposed residues listed as catalytic in the CSA were considered. Of the total 169 EC sub-subclasses present in the version of the CSA they used, 110 included instances of non-homologous enzymes as defined in SCOP [34]. Of these 110 EC sub-subclasses, 67 were shown to present pairs of enzymes with at least one matching catalytic residue. Finally, by inspecting the literature, 26 of these 67 sub-subclasses were identified as having pairs of enzymes with structurally equivalent active site residues playing equivalent roles in catalysis.

Because the enzymes in MACiE used in this study are almost a perfect subset of the enzymes in the CSA used by Gherardini and colleagues, the results reported in this previous work can be applicable to the dataset of enzymes studied here. In total, 78 of the 80 enzymes in our dataset were also present in version 2.2.2 of the CSA used in Gherardini et al.'s work. The enzymes present in our dataset but not in version 2.2.2 of the CSA were NAD+ synthase (MACiE M0200, EC 6.3.1.5, PDB 1kqp) [111]–[113] and uroporphyrinogen-III synthase (MACiE M0204, EC 4.2.1.75, PDB 1jr2) [114]–[117], both of which are reported in MACiE to catalyze their reactions without involvement of catalytic residues. In MACiE and the CSA, catalytic species (amino acid residues, organic cofactors and metal ions) can be generally divided into two types [47]: reactants, which undergo change in either charge state or covalent bonding; and spectators, which exert an electrostatic or steric effect upon another chemical species that is important for the reaction to occur, but do not change charge state or covalent bonding during catalysis. Those residues which only bind the substrate, but do not influence enzyme activity, are not considered catalytic.

We used Gherardini at al.'s definitions of active site similarity in the work reported here. Specifically, correspondences between PDB codes of MACiE entries used in our work and the PDB codes from the CSA listed in Table 3 entitled “Instances of convergent evolution” in Gherardini et al.'s work [11] were identified using the CSA homolog listings facility in the online version of MACiE [30]. Information about metal ions and organic cofactors used by the enzymes in the dataset was obtained directly from the online version of MACiE, and its sister Metal-MACiE database [27],[28].

## Supporting Information

Figure S1 Coverage of the dataset of functional analogs and of the background dataset.
(0.26 MB PDF)

Figure S2 Example overall reactions.
(0.50 MB PDF)

Figure S3 Bond types shared in the overall reactions of functional analogs.
(0.29 MB PDF)

Figure S4 Example catalytic mechanisms.
(0.71 MB PDF)

Table S1 Dataset of functionally analogous enzymes.
(0.15 MB DOC)

Table S2 Background dataset.
(0.12 MB DOC)

Table S3 Overall reaction and mechanistic similarity, and step alignments for the 95 pairs of enzyme reactions in the dataset.
(0.18 MB DOC)

Table S4 Summary of overall reaction similarity.
(0.03 MB DOC)

Table S5 Summary of mechanistic similarity.
(0.03 MB DOC)

Table S6 Cases of convergence of active sites in the dataset of functionally analogous enzymes, as identified by Gherardini and colleagues [11].
(0.04 MB DOC)

Table S7 Set of bond changes for every overall reaction and mechanistic step used in this work.
(0.09 MB DOC)

Text S1 Mapping between catalytic activities within the molecular function ontology of the Gene Ontology and the codes in the Enzyme Commission classification.
(0.06 MB PDF)
