# Supplementary material for: Quantitative Comparison of Catalytic Mechanisms and Overall Reactions in Convergently Evolved Enzymes: Implications for Classification of Enzyme Function
Source: PLoS Comput Biol. 2010 Mar 12;6(3):e1000700. doi: 10.1371/journal.pcbi.1000700 (PMC2837397; doi:10.1371/journal.pcbi.1000700)
Supplement: Figure S1 — Coverage of the dataset of functional analogs and of the background dataset. (0.26 MB PDF) [file pcbi.1000700.s001.pdf]

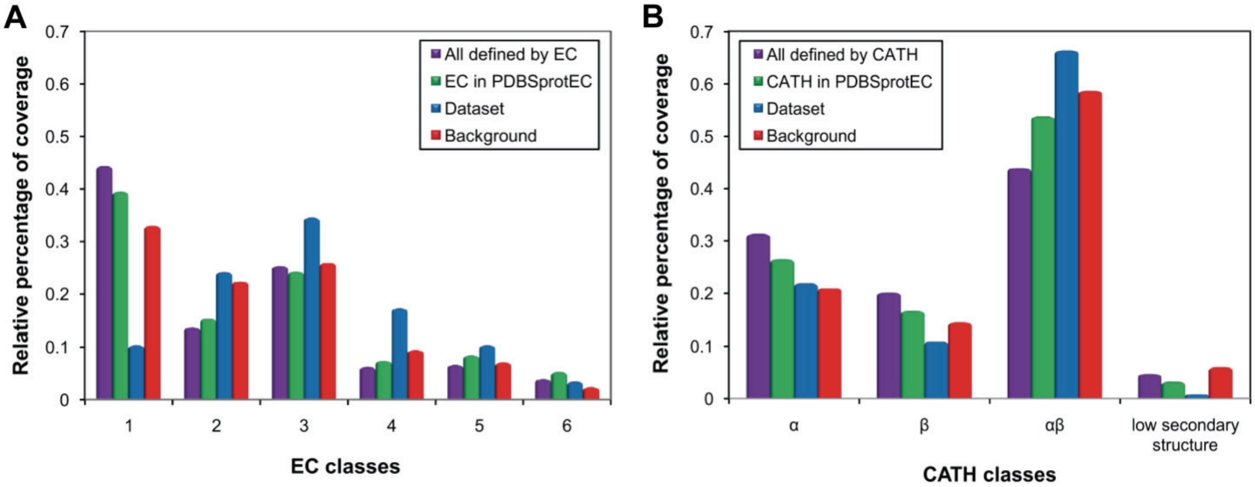

Figure S1. Coverage of the dataset of functional analogs and of the background dataset. (A) Distribution of sub-subclasses per EC class. (B) Distribution of superfamilies per CATH class.
