# Supplementary material for: Quantitative Comparison of Catalytic Mechanisms and Overall Reactions in Convergently Evolved Enzymes: Implications for Classification of Enzyme Function
Source: PLoS Comput Biol. 2010 Mar 12;6(3):e1000700. doi: 10.1371/journal.pcbi.1000700 (PMC2837397; doi:10.1371/journal.pcbi.1000700)
Supplement: Figure S2 — Example overall reactions. (0.50 MB PDF) [file pcbi.1000700.s002.pdf]

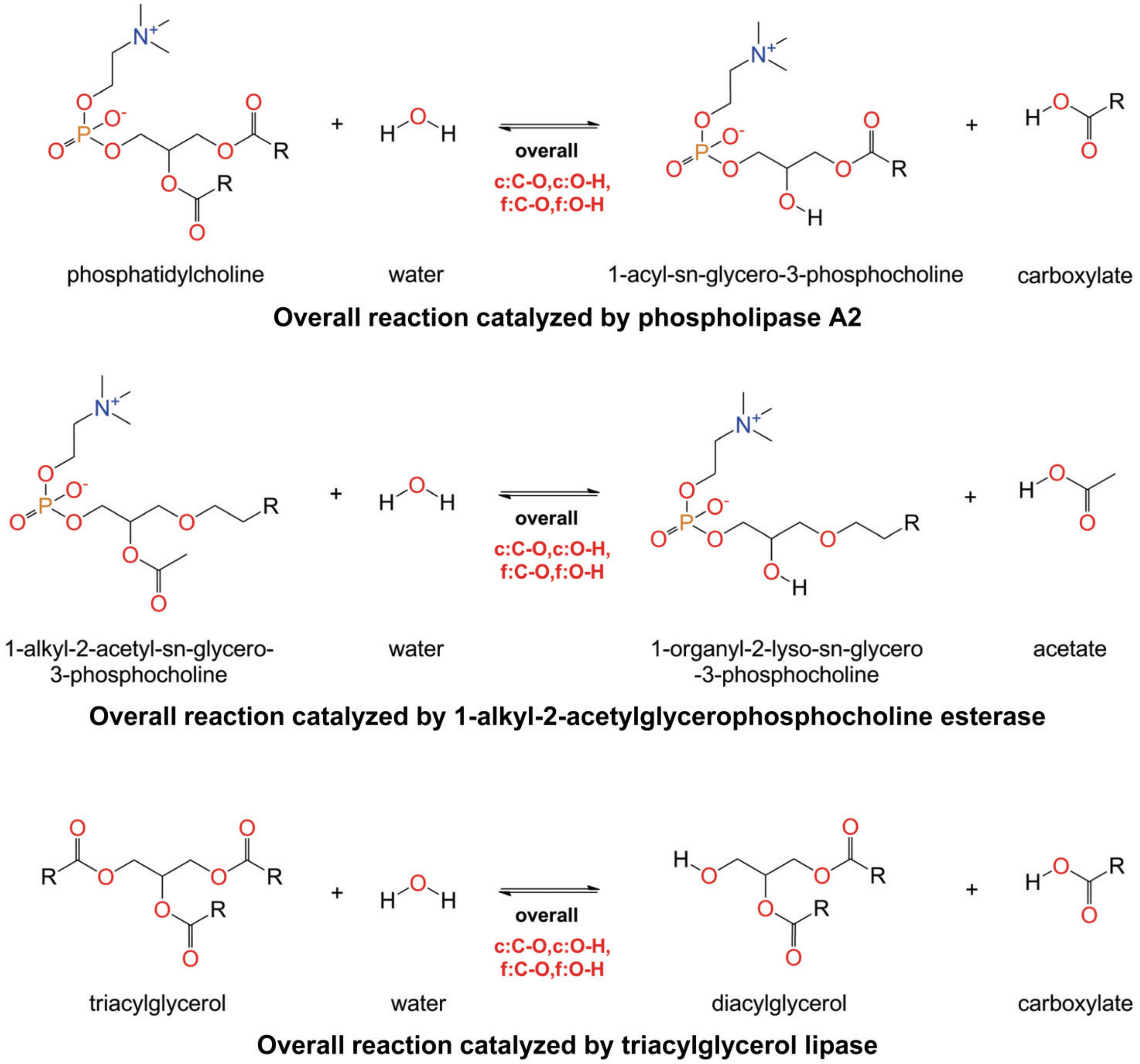

Figure S2. Example overall reactions. Overall reactions catalyzed by phospholipase A2 (MACiE M0083, EC 3.1.1.4, PDB 1l8s) [60,61], 1-alkyl-2-acetylglycerophosphocholine esterase (MACiE M0094, EC 3.1.1.47, PDB 1bwp) [62,63], and triacylglycerol lipase (MACiE M0218, EC 3.1.1.3, PDB 1hpl) [64-67]. Sets of overall bond changes are shown in red under the reaction arrows, with c: bond cleavage, d: decrease of bond order, f: bond formation, and i: increase of bond order. Note that all three overall reactions have symmetric (palindromic) bond changes consisting of the cleavage and formation of C-O bonds accompanied by the cleavage and formation of O-H bonds.
