# Supplementary material for: Quantitative Comparison of Catalytic Mechanisms and Overall Reactions in Convergently Evolved Enzymes: Implications for Classification of Enzyme Function
Source: PLoS Comput Biol. 2010 Mar 12;6(3):e1000700. doi: 10.1371/journal.pcbi.1000700 (PMC2837397; doi:10.1371/journal.pcbi.1000700)
Supplement: Figure S3 — Bond types shared in the overall reactions of functional analogs. (0.29 MB PDF) [file pcbi.1000700.s003.pdf]

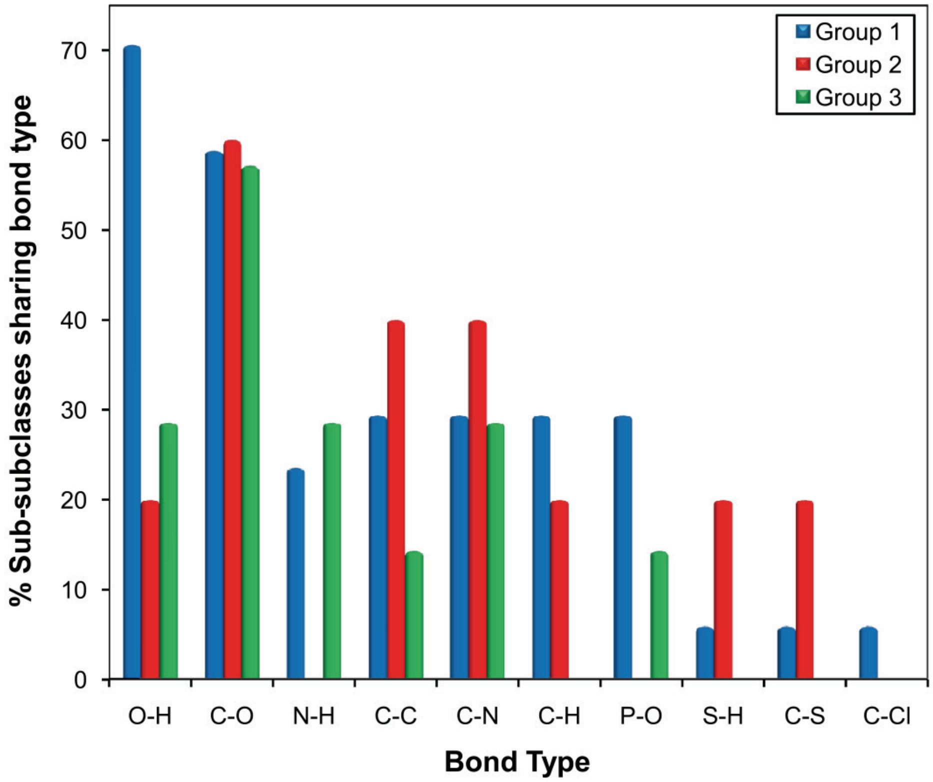

Figure S3. Bond types shared in the overall reactions of functional analogs. Enzymes were clustered according to similarities of overall reactions into three groups as defined in Table 1 (group 1 consisted only of EC sub-subclasses with similar overall reactions, group 2 consisted of EC sub-subclasses with both similar and non-similar reactions, and group 3 consisted only of EC sub-subclasses with non-similar reactions). Only bond types conserved in all members of a sub-subclass were considered, displayed from most common (left) to least common (right). These ten bond types correspond, from left to right, to the 1st, 2nd, 3rd, 4th, 5th, 6th, 7th, 8th, 10th and 37th most common bond types involved in enzyme catalyzed reactions according to Figure 10 in reference [47].
