# Supplementary material for: Quantitative Comparison of Catalytic Mechanisms and Overall Reactions in Convergently Evolved Enzymes: Implications for Classification of Enzyme Function
Source: PLoS Comput Biol. 2010 Mar 12;6(3):e1000700. doi: 10.1371/journal.pcbi.1000700 (PMC2837397; doi:10.1371/journal.pcbi.1000700)
Supplement: Figure S4 — Example catalytic mechanisms. (0.71 MB PDF) [file pcbi.1000700.s004.pdf]

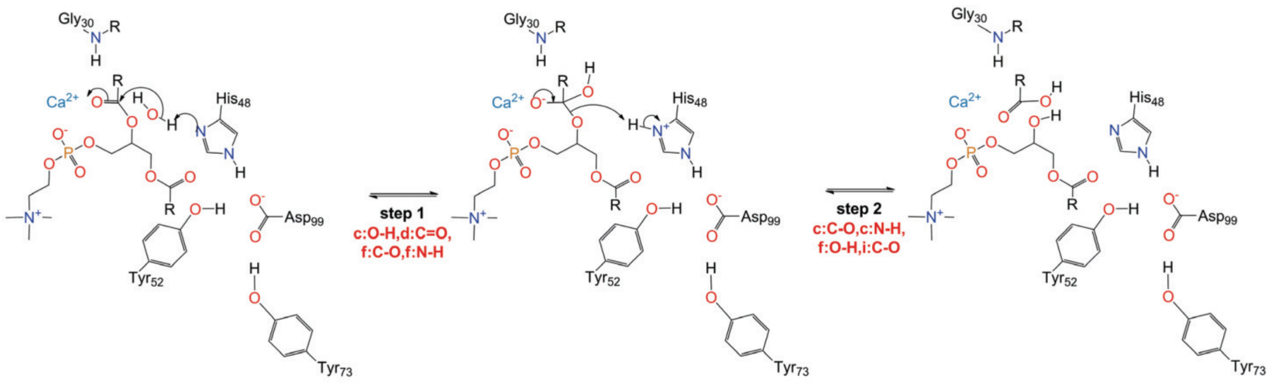

Mechanism catalyzed by phospholipase A2

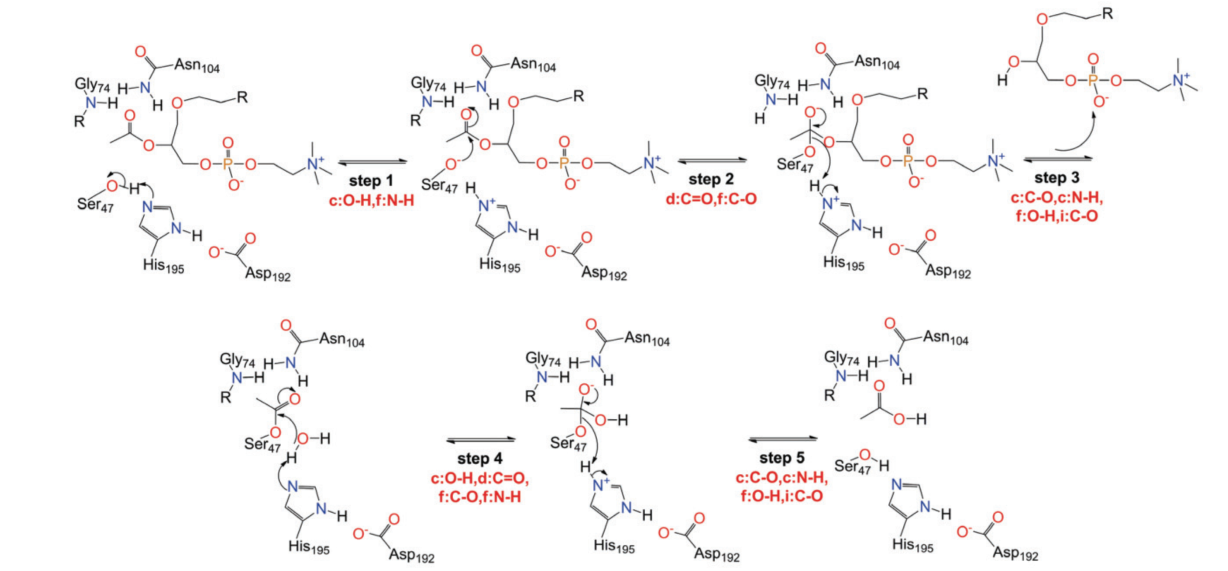

Mechanism catalyzed by 1-alkyl-2-acetylglycerophosphocholine esterase

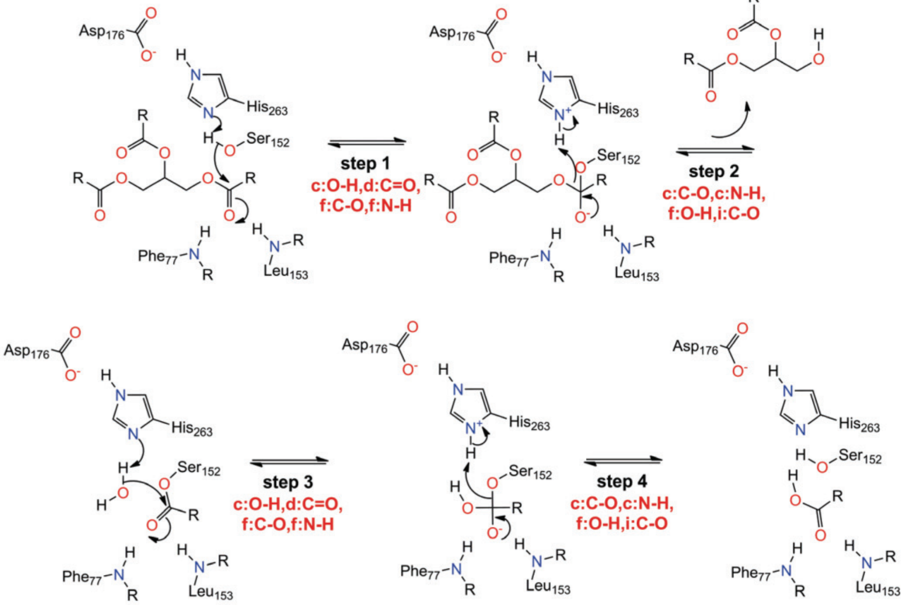

Mechanism catalyzed by triacylglycerol lipase

Figure S4. Example catalytic mechanisms. Catalytic mechanisms of phospholipase A2 (MACiE M0083, EC 3.1.1.4, PDB 1l8s) [60,61], 1-alkyl-2-acetylglycerophosphocholine esterase (MACiE M0094, EC 3.1.1.47, PDB 1bwp) [62,63], and triacylglycerol lipase (MACiE M0218, EC 3.1.1.3, PDB 1hpl) [64-67]. Sets of bond changes for each mechanistic step are shown in red under the reaction arrows, with c: bond cleavage, d: decrease of bond order, f: bond formation, and i: increase of bond order. The two mechanistic steps catalyzed by phospholipase A2 are identical to the last two steps catalyzed by triacylglycerol lipase and the last two steps catalyzed by 1-alkyl-2-acetylglycerophosphocholine esterase. These shared steps are the nucleophilic addition of a water molecule (activated by an Asp-His catalytic dyad) to an ester to form an oxyanionic species, and the subsequent collapse of the oxyanionic species to produce the products. Furthermore, the other steps of the reactions catalyzed by triacylglycerol lipase and 1-alkyl-2-acetylglycerophosphocholine esterase are identical in terms of bond changes, except that the first step of the former reaction (nucleophilic addition of a serine residue, activated by an Asp-His dyad, to an ester) is divided into two steps in the latter (activation of the serine residue by the dyad, followed in a separate step by addition of the serine to the ester). However, the mechanism for 1-alkyl-2-acetylglycerophosphocholine esterase present in MACiE was inferred from literature references that did not specify the sequence of steps [62,63] (see Limitations of the Study). A mechanism for 1-alkyl-2-acetylglycerophosphocholine esterase in which the first two steps are combined into one could thus be equally plausible. Considering this new mechanism, the four steps catalyzed by triacylglycerol lipase would be identical to the four (not five) steps catalyzed by 1-alkyl-2-acetylglycerophosphocholine esterase. Despite this difference in the number of steps found in MACiE for this pair of reactions, the method used to measure mechanistic similarity still identified these mechanisms as highly similar, indicating its robustness towards alternative positioning of specific bond changes in a reaction mechanism [37].
