# Supplementary material for: Quantitative Comparison of Catalytic Mechanisms and Overall Reactions in Convergently Evolved Enzymes: Implications for Classification of Enzyme Function
Source: PLoS Comput Biol. 2010 Mar 12;6(3):e1000700. doi: 10.1371/journal.pcbi.1000700 (PMC2837397; doi:10.1371/journal.pcbi.1000700)
Supplement: Table S1 — Dataset of functionally analogous enzymes. (0.15 MB DOC) [file pcbi.1000700.s005.doc]

Table S1. Dataset of functionally analogous enzymes.

| **EC code** | **Enzyme name** | **MACiE** | **PDB** | **CATH domain^a^** |
| --- | --- | --- | --- | --- |
| - - 1. **[1.Oxidoreductases; 1.1.Acting on the CH-OH group of donors; 1.1.1.With NAD+ or NADP+ as**   **acceptor]** | | | | |
| 1.1.1.22 | UDP-glucose 6-dehydrogenase | M0092 | 1dli | **1.10.1040.10**; **3.40.50.720**; 3.40.50.1870 |
| 1.1.1.34 | hydroxymethylglutaryl-CoA reductase (NADPH) | M0093 | 1dqa | 1.10.3270.10; **3.30.70.420**; **3.90.770.10** |
| 1.1.1.42 | isocitrate dehydrogenase (NADP+) | M0007 | 5icd | **3.40.718.10** |
| **1.3.99 [1.Oxidoreductases; 1.3.Acting on the CH-CH group of donors ; 1.3.99.With other acceptors]** | | | | |
| 1.3.99.1 | succinate dehydrogenase | M0020 | 1qjd | 1.10.1130.10; **3.50.50.60**; **3.90.700.10** |
| 1.3.99.10 | isovaleryl-CoA dehydrogenase | M0068 | 1ivh | 1.10.540.10; **1.20.140.10**; **2.40.110.10** |
| **1.5.1 [1.Oxidoreductases; 1.5.Acting on the CH-NH group of donors; 1.5.1.With NAD+ or NADP+ as**  **acceptor]** | | | | |
| 1.5.1.3 | dihydrofolate reductase | M0112 | 3dfr | **3.40.430.10** |
| 1.5.1.20 | methylenetetrahydrofolate reductase [NAD(P)H] | M0120 | 1zp3 | **3.20.20.220** |
| **2.1.1 [2.Transferases; 2.1.Transferring one-carbon groups; 2.1.1.Methyltransferases]** | | | | |
| 2.1.1.45 | thymidylate synthase | M0031 | 1lcb | **3.30.572.10** |
| 2.1.1.72 | site-specific DNA-methyltransferase (adenine-specific) | M0046 | 2adm | **3.40.50.150**; 3.90.220.10 |
| **2.2.1 [2.Transferases; 2.2.Transferring aldehyde or ketonic groups; 2.2.1.Transketolases and transaldolases]** | | | | |
| 2.2.1.1 | transketolase | M0219 | 1trk | 3.40.50.920; **3.40.50.970** |
| 2.2.1.2 | transaldolase | M0148 | 1onr | **3.20.20.70** |
| **2.3.1 [2.Transferases; 2.3.Acyltransferases; 2.3.1.Transferring groups other than aminoacyl groups]** | | | | |
| 2.3.1.16 | acetyl-CoA C-acyltransferase | M0077 | 1afw | **3.40.47.10**; **3.40.47.20** |
| 2.3.1.54 | formate C-acetyltransferase | M0030 | 2pfl | **3.20.70.20** |
| 2.3.1.87 | aralkylamine N-acetyltransferase | M0022 | 1b6b | **3.40.630.30** |
| 2.3.1.129 | acyl-[acyl-carrier-protein]-UDP-N-acetylglucosamine O-acyltransferase | M0069 | 1lxa | 1.20.1180.10; **2.160.10.10** |
| **2.3.3 [2.Transferases; 2.3.Acyltransferases; 2.3.3.Acyl groups converted into alkyl groups on transfer]** | | | | |
| 2.3.3.1 | citrate (Si)-synthase | M0078 | 1al6 | **1.10.230.10**; **1.10.580.10** |
| 2.3.3.9 | malate synthase | M0053 | 1d8c | 1.10.1860.10; **1.20.1220.12**; 2.170.170.11; **3.20.20.360** |
| **2.4.1 [2.Transferases; 2.4.Glycosyltransferases; 2.4.1.Hexosyltransferases]** | | | | |
| 2.4.1.1 | phosphorylase | M0205 | 1gpb | **3.40.50.2000** |
| 2.4.1.19 | cyclomaltodextrin glucanotransferase | M0045 | 1cdg | 2.60.40.10; 2.60.40.1180; **3.20.20.80** |
| **2.4.2 [2.Transferases; 2.4.Glycosyltransferases; 2.4.2.Pentosyltransferases]** | | | | |
| 2.4.2.1 | purine-nucleoside phosphorylase | M0017 | 1ula | **3.40.50.1580** |
| 2.4.2.2 | pyrimidine-nucleoside phosphorylase | M0091 | 1brw | 1.20.970.10; **3.40.1030.10**; 3.90.1170.30 |
| 2.4.2.14 | amidophosphoribosyltransferase | M0214 | 1ecf | 3.40.50.2020; **3.60.20.10** |
| 2.4.2.19 | nicotinate-nucleotide diphosphorylase (carboxylating) | M0008 | 1qpr | **3.20.20.70**; **3.90.1170.20** |
| 2.4.2.21 | nicotinate-nucleotide-dimethylbenzimidazole phosphoribosyltransferase | M0079 | 1d0s | 1.10.1610.10; **3.40.50.10210** |
| 2.4.2.30 | NAD+ ADP-ribosyltransferase | M0076 | 1a26 | 1.20.142.10; **3.90.228.10** |
| **2.6.1 [2.Transferases; 2.6.Transferring nitrogenous groups; 2.6.1.Transaminases]** | | | | |
| 2.6.1.16 | glutamine-fructose-6-phosphate transaminase (isomerizing) | M0082 | 1jxa | **3.40.50.10490**; **3.60.20.10** |
| 2.6.1.21 | D-amino-acid transaminase | M0066 | 1daa | **3.20.10.10**; **3.30.470.10** |
| **3.1.1 [3.Hydrolases; 3.1.Acting on ester bonds; 3.1.1.Carboxylic-ester hydrolases]** | | | | |
| 3.1.1.3 | triacylglycerol lipase | M0218 | 1hpl | 2.60.60.20; **3.40.50.1820** |
| 3.1.1.4 | phospholipase A2 | M0083 | 1l8s | **1.20.90.10** |
| 3.1.1.47 | 1-alkyl-2-acetylglycerophosphocholine esterase | M0094 | 1bwp | **3.40.50.1110** |
| **3.1.21 [3.Hydrolases; 3.1.Acting on ester bonds; 3.1.21.Endodeoxyribonucleases producing 5'-**  **phosphomonoesters]** | | | | |
| 3.1.21.1 | deoxyribonuclease I | M0041 | 1dnk | **3.60.10.10** |
| 3.1.21.2 | deoxyribonuclease IV (phage-T4-induced) | M0011 | 1qum | **3.20.20.150** |
| **3.1.3 [3.Hydrolases; 3.1.Acting on ester bonds; 3.1.3.Phosphoric-monoester hydrolases]** | | | | |
| 3.1.3.1 | alkaline phosphatase | M0044 | 1alk | **3.40.720.10** |
| 3.1.3.2 | acid phosphatase | M0043 | 4kbp | 2.60.40.380; **3.60.21.10** |
| 3.1.3.48 | protein-tyrosine-phosphatase | M0047 | 1ytw | **3.90.190.10** |
| **3.1.4 [3.Hydrolases; 3.1.Acting on ester bonds; 3.1.4.Phosphoric-diester hydrolases]** | | | | |
| 3.1.4.3 | phospholipase C | M0027 | 1ah7 | **1.10.575.10** |
| 3.1.4.11 | phosphoinositide phospholipase C | M0028 | 1djx | 1.10.238.10; 2.60.40.150; **3.20.20.190** |
| **3.2.1 [3.Hydrolases; 3.2.Glycosylases; 3.2.1.Glycosidases, i.e. hydrolysing O- and S-glycosyl compounds]** | | | | |
| 3.2.1.17 | lysozyme | M0203 | 1dpx | **1.10.530.10** |
| 3.2.1.113 | mannosyl-oligosaccharide 1,2-alpha-mannosidase | M0019 | 1dl2 | **1.50.10.50** |
| **3.2.2 [3.Hydrolases; 3.2.Glycosylases; 3.2.2.Hydrolysing N-glycosyl compounds]** | | | | |
| 3.2.2.1 | purine nucleosidase | M0039 | 2mas | **3.90.245.10** |
| 3.2.2.3 | uridine nucleosidase | M0071 | 1eug | **3.40.470.10** |
| **3.5.1 [3.Hydrolases; 3.5.Acting on carbon-nitrogen bonds, other than peptide bonds; 3.5.1.In linear amides]** | | | | |
| 3.5.1.5 | urease | M0087 | 1fwj | 2.10.150.10; 2.30.40.10; **3.20.20.140**; 3.30.280.10 |
| 3.5.1.38 | glutamin-(asparagin-)ase | M0029 | 1djo | **3.40.50.40**; **3.40.50.1170** |
| 3.5.1.59 | N-carbamoylsarcosine amidase | M0025 | 1nba | **3.40.50.850** |
| 3.5.1.88 | peptide deformylase | M0098 | 1bsz | **3.90.45.10** |
| **3.5.2 [3.Hydrolases; 3.5.Acting on carbon-nitrogen bonds, other than peptide bonds; 3.5.2.In cyclic amides]** | | | | |
| 3.5.2.6 | beta-lactamase {Class A} | M0002 | 1btl | **3.40.710.10** |
| 3.5.2.6 | beta-lactamase {Class B} | M0016 | 1bc2 | **3.60.15.10** |
| **3.5.4 [3.Hydrolases; 3.5.Acting on carbon-nitrogen bonds, other than peptide bonds; 3.5.4.In cyclic**  **amidines]** | | | | |
| 3.5.4.5 | cytidine deaminase | M0097 | 1ctt | **3.40.140.10** |
| 3.5.4.16 | GTP cyclohydrolase I | M0038 | 1fbx | 1.10.286.10; **3.30.1130.10** |
| **3.8.1 [3.Hydrolases; 3.8.Acting on halide bonds; 3.8.1.In carbon-halide compounds]** | | | | |
| 3.8.1.2 | (S)-2-haloacid dehalogenase | M0036 | 1qq5 | **1.10.164.10**; **3.40.50.1000** |
| 3.8.1.7 | 4-chlorobenzoyl-CoA dehalogenase | M0024 | 1nzy | 1.10.12.10; **3.90.226.10** |
| **4.1.1 [4.Lyases; 4.1.Carbon-carbon lyases; 4.1.1.Carboxy-lyases]** | | | | |
| 4.1.1.1 | pyruvate decarboxylase | M0215 | 1pvd | **3.40.50.970**; 3.40.50.1220 |
| 4.1.1.22 | histidine decarboxylase | M0049 | 1pya | **3.50.20.10**; **4.10.510.10** |
| 4.1.1.23 | orotidine-5'-phosphate decarboxylase | M0050 | 1dbt | **3.20.20.70** |
| 4.1.1.41 | methylmalonyl-CoA decarboxylase | M0070 | 1ef8 | 1.10.12.10; **3.90.226.10** |
| 4.1.1.49 | phosphoenolpyruvate carboxykinase (ATP) | M0051 | 1aq2 | **2.170.8.10**; 3.40.449.10; **3.90.228.20** |
| **4.1.2 [4.Lyases; 4.1.Carbon-carbon lyases; 4.1.2.Aldehyde-lyases]** | | | | |
| 4.1.2.13 | fructose-bisphosphate aldolase {Class I} | M0222 | 2qut | **3.20.20.70** |
| 4.1.2.17 | L-fuculose-phosphate aldolase | M0072 | 1fua | **3.40.225.10** |
| 4.1.2.37 | hydroxynitrilase | M0217 | 1sc9 | **3.40.50.1820** |
| **4.2.1 [4.Lyases; 4.2.Carbon-oxygen lyases; 4.2.1.Hydro-lyases]** | | | | |
| 4.2.1.1 | carbonate dehydratase | M0216 | 1ca2 | **3.10.200.10** |
| 4.2.1.10 | 3-dehydroquinate dehydratase {type I} | M0054 | 1qfe | **3.20.20.70** |
| 4.2.1.10 | 3-dehydroquinate dehydratase {type II} | M0055 | 1gu1 | **3.40.50.9100** |
| 4.2.1.60 | 3-hydroxydecanoyl-[acyl-carrier-protein] dehydratase | M0010 | 1mkb | **3.10.129.10** |
| 4.2.1.75 | uroporphyrinogen-III synthase | M0204 | 1jr2 | **3.40.50.10090** |
| 4.2.1.84 | nitrile hydratase | M0057 | 2ahj | **1.10.472.20**; 2.30.30.50; **3.90.330.10** |
| 4.2.1.96 | 4a-hydroxytetrahydrobiopterin dehydratase | M0073 | 1dco | **3.30.1360.20** |
| **4.2.3 [4.Lyases; 4.2.Carbon-oxygen lyases; 4.2.3.Acting on phosphates]** | | | | |
| 4.2.3.3 | methylglyoxal synthase | M0085 | 1b93 | **3.40.50.1380** |
| 4.2.3.4 | 3-dehydroquinate synthase | M0059 | 1dqs | **1.20.1090.10**; **3.40.50.1970** |
| 4.2.3.7 | pentalenene synthase | M0089 | 1ps1 | **1.10.600.10** |
| 4.2.3.12 | 6-pyruvoyltetrahydropterin synthase | M0084 | 1b66 | **3.30.479.10** |
| **4.6.1 [4.Lyases; 4.6.Phosphorus-oxygen lyases; 4.6.1.Phosphorus-oxygen lyases]** | | | | |
| 4.6.1.1 | adenylate cyclase | M0058 | 1cju | 1.10.400.10; **3.30.70.1230**; 3.40.50.300 |
| 4.6.1.13 | phosphatidylinositol diacylglycerol-lyase | M0026 | 1ptd | **3.20.20.190** |
| **5.1.1 [5.Isomerases; 5.1.Racemases and epimerases; 5.1.1.Acting on amino acids and derivates]** | | | | |
| 5.1.1.1 | alanine racemase | M0213 | 1xfc | **2.40.37.10**; **3.20.20.10** |
| 5.1.1.3 | glutamate racemase | M0001 | 1b73 | **3.40.50.1860** |
| **5.4.2 [5.Isomerases; 5.4.Intramolecular transferases; 5.4.2.Phosphotransferases (phosphomutases)]** | | | | |
| 5.4.2.6 | beta-phosphoglucomutase | M0206 | 1o08 | 1.10.164.10; **3.40.50.1000** |
| 5.4.2.8 | phosphomannomutase | M0194 | 1p5d | 3.30.310.50; **3.40.120.10** |
| **5.4.99 [5.Isomerases; 5.4.Intramolecular transferases; 5.4.99.Transferring other groups]** | | | | |
| 5.4.99.2 | methylmalonyl-CoA mutase | M0062 | 1req | **3.20.20.240**; **3.40.50.280** |
| 5.4.99.5 | chorismate mutase | M0081 | 3csm | **1.10.590.10** |
| **6.3.1 [6.Ligases; 6.3.Forming carbon-nitrogen bonds; 6.3.1.Acid-ammonia (or amine) ligases (amide**  **synthases)]** | | | | |
| 6.3.1.1 | aspartate-ammonia ligase | M0075 | 12as | **3.30.930.10** |
| 6.3.1.5 | NAD+ synthase | M0200 | 1kqp | **3.40.50.620** |

^a^Catalytic domains are shown in bold.
