# Supplementary material for: Quantitative Comparison of Catalytic Mechanisms and Overall Reactions in Convergently Evolved Enzymes: Implications for Classification of Enzyme Function
Source: PLoS Comput Biol. 2010 Mar 12;6(3):e1000700. doi: 10.1371/journal.pcbi.1000700 (PMC2837397; doi:10.1371/journal.pcbi.1000700)
Supplement: Table S2 — Background dataset. (0.12 MB DOC) [file pcbi.1000700.s006.doc]

**Table S2. Background dataset.**

^a^Catalytic domains are shown in bold.

| **EC code** | **Enzyme name** | **MACiE** | **PDB** | **CATH domain^a^** |
| --- | --- | --- | --- | --- |
| **1.1.5.2** | quinoprotein glucose dehydrogenase | M0104 | 1c9u | **2.120.10.30** |
| **1.1.99.8** | quinoprotein alcohol dehydrogenase | M0099 | 1g72 | **2.140.10.10**; 4.10.160.10 |
| **1.2.1.8** | betaine-aldehyde dehydrogenase | M0100 | 1a4s | **3.40.309.10**; **3.40.605.10** |
| **1.4.99.3** | amine dehydrogenase | M0013 | 2bbk | **2.60.30.10**; 2.130.10.10 |
| **1.6.5.2** | NAD(P)H dehydrogenase (quinone) | M0003 | 1d4a | **3.40.50.360** |
| **1.7.1.-** | oxygen insensitive NAD(P)H nitroreductase | M0211 | 1idt | **3.40.109.10** |
| **1.7.3.3** | urate oxidase | M0118 | 1wrr | **3.10.270.10** |
| **1.8.3.1** | sulfite oxidase | M0121 | 1sox | 2.60.40.650; 3.10.120.10; **3.90.420.10** |
| **1.8.4.6** | protein-methionine-S-oxide reductase | M0122 | 1fva | **3.30.1060.10** |
| **1.9.3.1** | cytochrome-c oxidase | M0124 | 1v54 | 1.10.10.140; 1.10.287.70; 1.10.287.90; 1.10.442.10; 1.20.120.80; **1.20.210.10**; 1.25.40.40; 2.60.11.10; 2.60.40.420; 4.10.49.10; 4.10.51.10; 4.10.81.10; 4.10.91.10; 4.10.93.10; 4.10.95.10 |
| **1.10.3.1** | catechol oxidase | M0125 | 1bt3 | **1.10.1280.10** |
| **1.11.1.10** | chloride peroxidase | M0014 | 1vnc | **1.10.606.10**; 1.20.144.10 |
| **1.12.2.1** | cytochrome-c3 hydrogenase | M0126 | 2frv | **1.10.645.10**; 3.40.50.700; 4.10.480.10 |
| **1.12.7.2** | ferredoxin hydrogenase | M0127 | 1hfe | 3.30.70.20; **3.40.50.1780**; **3.40.950.10**; 4.10.260.20 |
| **1.13.12.7** | photinus-luciferin 4-monooxygenase (ATP-hydrolysing) | M0128 | 1ba3 | 2.30.38.10; **3.30.300.30**; **3.40.50.980**; 4.10.8.10 |
| **1.14.12.12** | naphthalene 1,2-dioxygenase | M0130 | 1ndo | **2.102.10.10**; 3.10.450.50; **3.90.380.10** |
| **1.14.14.3** | alkanal monooxygenase (FMN-linked) | M0132 | 1luc | **3.20.20.30** |
| **1.14.15.1** | camphor 5-monooxygenase | M0133 | 1yrc | **1.10.630.10** |
| **1.14.16.2** | tyrosine 3-monooxygenase | M0134 | 2toh | **1.10.800.10** |
| **1.14.17.3** | peptidylglycine monooxygenase | M0135 | 1sdw | **2.60.120.230**; **2.60.120.310** |
| **1.14.19.2** | acyl-[acyl-carrier-protein] desaturase | M0136 | 1afr | **1.10.620.20** |
| **1.14.20.1** | deacetoxycephalosporin-C synthase | M0137 | 1unb | **2.60.120.330** |
| **1.14.99.1** | prostaglandin-endoperoxide synthase | M0037 | 5cox | **1.10.640.10**; 2.10.25.10 |
| **1.15.1.1** | superoxide dismutase | M0138 | 2jcw | **2.60.40.200** |
| **1.17.4.2** | ribonucleoside-triphosphate reductase | M0140 | 1l1l | **3.20.70.20**; 3.30.1620.10; 3.90.1390.10 |
| **1.17.99.1** | 4-cresol dehydrogenase (hydroxylating) | M0141 | 1dii | **1.10.45.10**; **1.10.760.10**; 3.30.43.10; **3.30.465.20**; **3.40.462.10** |
| **1.18.1.2** | ferredoxin-NADP+ reductase | M0142 | 1e6e | 3.10.20.30; **3.40.50.720**; **3.50.50.60** |
| **1.20.4.1** | arsenate reductase (glutaredoxin) | M0143 | 1ljl | **3.40.50.270** |
| **2.1.1.20** | glycine N-methyltransferase | M0023 | 1xva | 3.30.46.10; **3.40.50.150** |
| **2.1.2.1** | glycine hydroxymethyltransferase | M0147 | 1ls3 | **3.40.640.10**; 3.90.1150.10 |
| **2.1.3.3** | ornithine carbamoyltransferase | M0012 | 1oth | **3.40.50.1370** |
| **2.1.4.1** | glycine amidinotransferase | M0018 | 1jdw | **3.75.10.10** |
| **2.3.2.13** | protein-glutamine gamma-glutamyltransferase | M0149 | 1ggt | 2.60.40.10; **2.60.40.30**; **3.90.260.10** |
| **2.4.2.8** | hypoxanthine phosphoribosyltransferase | M0048 | 1bzy | **3.40.50.2020** |
| **2.5.1.6** | methionine adenosyltransferase | M0009 | 1p7l | **3.30.300.10** |
| **2.7.1.20** | adenosine kinase | M0209 | 1lij | **3.30.1110.10**; **3.40.1190.20** |
| **2.7.2.3** | phosphoglycerate kinase | M0040 | 13pk | **3.40.50.1260**; **3.40.50.1270** |
| **2.7.3.3** | arginine kinase | M0086 | 1bg0 | 1.10.135.10; **3.30.590.10** |
| **2.7.4.6** | nucleoside-diphosphate kinase | M0150 | 1kdn | **3.30.70.141** |
| **2.7.6.3** | 2-amino-4-hydroxy-6-hydroxymethyldihydropteridine diphosphokinase | M0151 | 1q0n | **3.30.70.560** |
| **2.7.7.12** | UDP-glucose-hexose-1-phosphate uridylyltransferase | M0088 | 1hxq | **3.30.428.10** |
| **2.7.8.7** | holo-[acyl-carrier-protein] synthase | M0152 | 1f7l | **3.90.470.20** |
| **2.7.9.1** | pyruvate, phosphate dikinase | M0207 | 1kc7 | 1.10.189.10; 1.20.80.30; **3.20.20.60**; **3.30.470.20**; **3.30.1490.20**; **3.50.30.10** |
| **2.7.11.19** | phosphorylase kinase | M0035 | 2phk | **1.10.510.10**; 3.30.200.20 |
| **2.8.1.1** | thiosulfate sulfurtransferase | M0153 | 1rhs | **3.40.250.10** |
| **2.8.2.4** | estrone sulfotransferase | M0154 | 1hy3 | **3.40.50.300** |
| **2.8.4.1** | coenzyme-B sulfoethylthiotransferase | M0156 | 1mro | **1.20.840.10**; **3.30.70.470**; 3.90.320.20; 3.90.390.10 |
| **3.1.2.6** | hydroxyacylglutathione hydrolase | M0157 | 1qh5 | **3.60.15.10** |
| **3.1.6.8** | cerebroside-sulfatase | M0158 | 1auk | 3.30.1120.10; **3.40.720.10** |
| **3.1.11.2** | exodeoxyribonuclease III | M0160 | 1ako | **3.60.10.10** |
| **3.1.25.1** | deoxyribonuclease (pyrimidine dimer) | M0162 | 1vas | **1.10.440.10** |
| **3.1.26.4** | calf thymus ribonuclease H | M0163 | 1rdd | **3.30.420.10** |
| **3.1.27.5** | pancreatic ribonuclease | M0164 | 1ruv | **3.10.130.10** |
| **3.1.30.2** | Serratia marcescens nuclease | M0042 | 1ql0 | **3.40.570.10** |
| **3.1.31.1** | micrococcal nuclease | M0165 | 2sns | **2.40.50.90** |
| **3.4.11.10** | bacterial leucyl aminopeptidase | M0167 | 1lok | **3.40.630.10** |
| **3.4.14.5** | dipeptidyl-peptidase IV | M0169 | 1pfq | 2.140.10.30; **3.40.50.1820** |
| **3.4.19.-** | isoaspartyl dipeptidase | M0172 | 1onw | 2.30.40.10; **3.20.20.140** |
| **3.4.21.4** | trypsin | M0173 | 1pq5 | **2.40.10.10** |
| **3.4.22.2** | papain | M0174 | 9pap | **3.90.70.10** |
| **3.4.23.16** | HIV-1 retropepsin | M0175 | 1a30 | **2.40.70.10** |
| **3.4.24.27** | thermolysin | M0176 | 1kei | **1.10.390.10**; **3.10.170.10** |
| **3.4.25.1** | proteasome endopeptidase complex | M0177 | 1ryp | **3.60.20.10** |
| **3.5.2.6** | beta-lactamase {Class D} | M0210 | 1m6k | **3.40.710.10** |
| **3.5.3.3** | creatinase | M0096 | 1chm | 3.40.350.10; **3.90.230.10** |
| **3.5.99.6** | glucosamine-6-phosphate deaminase | M0060 | 1dea | **3.40.50.1360** |
| **3.6.4.9** | chaperonin ATPase | M0179 | 1q3s | **1.10.560.10**; **3.30.260.10**; 3.50.7.10 |
| **3.7.1.2** | fumarylacetoacetase | M0180 | 1hyo | 2.30.30.230; **3.90.850.10** |
| **3.11.1.1** | phosphonoacetaldehyde hydrolase | M0181 | 1rql | **1.10.164.10**; **3.40.50.1000** |
| **4.1.1.7** | benzoylformate decarboxylase | M0220 | 1mcz | **3.40.50.970**; 3.40.50.1220 |
| **4.1.2.13** | fructose-bisphosphate aldolase (Class II) | M0052 | 1b57 | **3.20.20.70** |
| **4.1.99.3** | deoxyribodipyrimidine photo-lyase | M0183 | 1dnp | **1.10.579.10**; **1.25.40.80**; 3.40.50.620 |
| **4.2.2.2** | pectate lyase | M0184 | 1ru4 | **2.160.20.10** |
| **4.2.99.18** | DNA-(apurinic or apyrimidinic site) lyase | M0185 | 1lwy | **1.10.340.30**; **1.10.1670.10**; 3.30.310.40 |
| **4.3.1.17** | L-serine ammonia-lyase | M0186 | 1pwh | **3.40.50.1100** |
| **4.3.2.2** | adenylosuccinate lyase | M0080 | 1c3c | 1.10.40.30; **1.10.275.10**; **1.20.200.10** |
| **4.4.1.5** | lactoylglutathione lyase | M0032 | 1qin | **3.10.180.10** |
| **5.1.2.2** | mandelate racemase | M0187 | 1mns | **3.20.20.120**; 3.30.390.10 |
| **5.3.1.3** | arabinose isomerase | M0095 | 1fui | **3.20.14.10**; 3.40.50.1070; **3.40.275.10** |
| **5.3.3.2** | isopentenyl-diphosphate Delta-isomerase | M0190 | 1nfs | **3.90.79.10** |
| **5.3.4.1** | protein disulfide-isomerase | M0191 | 1mek | **3.40.30.10** |
| **5.4.99.1** | methylaspartate mutase | M0063 | 1cb7 | **3.20.20.240**; **3.40.50.280**; 3.90.970.10 |
| **5.5.1.6** | chalcone isomerase | M0196 | 1eyq | 1.10.890.20; **3.50.70.10** |
| **6.3.4.4** | adenylosuccinate synthase | M0065 | 1gim | 1.10.300.10; **3.40.440.10**; 3.90.170.10 |
| **6.5.1.1** | DNA ligase (ATP) | M0202 | 1a0i | 2.40.50.140; 3.30.470.30; **3.30.1490.70** |
