# Supplementary material for: Quantitative Comparison of Catalytic Mechanisms and Overall Reactions in Convergently Evolved Enzymes: Implications for Classification of Enzyme Function
Source: PLoS Comput Biol. 2010 Mar 12;6(3):e1000700. doi: 10.1371/journal.pcbi.1000700 (PMC2837397; doi:10.1371/journal.pcbi.1000700)
Supplement: Table S3 — Overall reaction and mechanistic similarity, and step alignments for the 95 pairs of enzyme reactions in the dataset. (0.18 MB DOC) [file pcbi.1000700.s007.doc]

**Table S3. Overall reaction and mechanistic similarity, and step alignments for the 95 pairs of enzyme reactions in the dataset**.

| **EC sub-subclass** | **EC sub-subclass definition** | **Pair** | **Overall Reaction Similarity^a^** | **Mechanistic Similarity^a^** | **Step alignments^b^** | |
| --- | --- | --- | --- | --- | --- | --- |
| **Group 1: Sub-subclasses containing only highly similar and/or distantly similar overall reactions** | | | | | | |
| 1.3.99 | Oxidoreductases; Acting on the CH-CH group of donors; With other acceptors | M0020-M0068 | 1.0000  (1.0000) | 0.1678  (0.2238) | stg03REV  stg02REV  stg01REV  - | stg01  stg02  stg03  stg04 |
| 1.5.1 | Oxidoreductases; Acting on the CH-NH group of donors; With NAD+ or NADP+ as acceptor | M0112-M0120 | 0.7500  (0.7500) | 0.1538  (0.2308) | stg01  stg02  -  - | -  stg01  stg02  stg03 |
| 2.2.1 | Transferases; Transferring aldehyde or ketonic groups; Transketolases and transaldolases | M0148-M0219 | 1.0000  (1.0000) | 0.1708  (0.2562) | stg09REV  stg08REV  stg07REV  stg06REV  stg05REV  stg04REV  stg03REV  stg02REV  stg01REV | stg01  -  stg02  stg03  -  stg04  -  stg05  stg06 |
| 2.3.3 | Transferases; Acyltransferases; Acyl groups converted into alkyl groups on transfer | M0053-M0078 | 0.8750  (1.0000) | 0.6579  (0.6579) | **stg01**  stg02  stg03 | **stg01**  stg02  stg03 |
| 2.6.1 | Transferases; Transferring nitrogenous groups; Transaminases | M0066-M0082 | 0.7500  (1.0000) | 0.2440  (0.2847) | stg12REV  stg11REV  stg10REV  stg09REV  stg08REV  -  stg07REV  -  **stg06REV**  **stg05REV**  stg04REV  stg03REV  **stg02REV**  stg01REV  -  -  -  - | stg01  stg02  -  -  stg03  stg04  stg05  stg06  **stg07**  **stg08**  -  -  **stg09**  stg10  stg11  stg12  stg13  stg14 |
| 3.1.1 | Hydrolases; Acting on ester bonds; Carboxylic-ester hydrolases | M0083-M0094 | 1.0000  (1.0000) | 0.4000  (1.0000) | -  -  -  **stg01**  **stg02** | stg01  stg02  stg03  **stg04**  **stg05** |
|  |  | M0083-M0218 | 1.0000  (1.0000) | 0.5000  (1.0000) | -  -  **stg01**  **stg02** | stg01  stg02  **stg03**  **stg04** |
|  |  | M0094-M0218 | 1.0000  (1.0000) | 0.6364  (0.7955) | stg01  stg02  **stg03**  **stg04**  **stg05** | -  stg01  **stg02**  **stg03**  **stg04** |
| 3.1.3 | Hydrolases; Acting on ester bonds; Phosphoric-monoester hydrolases | M0043-M0044 | 1.0000  (1.0000) | 0.1351  (0.1802) | -  stg01  stg02  stg03 | stg01  stg02  stg03  stg04 |
|  |  | M0043-M0047 | 1.0000  (1.0000) | 0.1077  (0.1077) | stg01  stg02  -  stg03 | -  stg01  stg02  stg03 |
|  |  | M0044-M0047 | 1.0000  (1.0000) | 0.4286  (0.5714) | stg01  stg02  stg03  **stg04** | stg01  -  stg02  **stg03** |
| 3.1.4 | Hydrolases; Acting on ester bonds; Phosphoric-diester hydrolases | M0027-M0028 | 1.0000  (1.0000) | 0.3333  (0.3333) | stg01  stg02 | stg01  stg02 |
| 3.1.21 | Hydrolases; Acting on ester bonds; Endodeoxyribonucleases producing 5'-phosphomonoesters | M0011-M0041 | 1.0000  (1.0000) | 0.1250  (0.2500) | stg01  - | stg01  stg02 |
| 3.2.1 | Hydrolases; Glycosylases; Glycosidases, i.e. hydrolysing O- and S-glycosyl compounds | M0019-M0203 | 1.0000  (1.0000) | 0.3333  (0.3333) | stg01  stg02 | stg01  stg02 |
| 3.5.1 | Hydrolases; Acting on carbon-nitrogen bonds, other than peptide bonds; In linear amides | M0025-M0029 | 1.0000  (1.0000) | 0.5000  (0.5000) | stg01  **stg02**  **stg03**  stg04 | stg01  **stg02**  **stg03**  stg04 |
|  |  | M0025-M0087 | 1.0000  (1.0000) | 0.0909  (0.1818) | stg01  stg02  stg03  stg04 | stg01  stg02  -  - |
|  |  | M0025-M0098 | 1.0000  (1.0000) | 0.2426  (0.3235) | stg01  stg02  stg03  stg04 | stg02  stg03  stg01  - |
|  |  | M0029-M0087 | 1.0000  (1.0000) | 0.1613  (0.3226) | stg01  stg02  stg03  stg04 | stg01  stg02  -  - |
|  |  | M0029-M0098 | 1.0000  (1.0000) | 0.3125  (0.4167) | stg01  stg02  stg03  stg04 | stg02  stg03  -  stg01 |
|  |  | M0087-M0098 | 1.0000  (1.0000) | 0.1299  (0.1949) | stg01  stg02  - | stg02  stg03  stg01 |
| 3.5.2 | Hydrolases; Acting on carbon-nitrogen bonds, other than peptide bonds; In cyclic amides | M0002-M0016 | 1.0000  (1.0000) | 0.2857  (0.3571) | stg01  -  **stg02**  stg03  stg04  stg05 | stg01  stg02  **stg03**  -  -  stg04 |
| 3.8.1 | Hydrolases; Acting on halide bonds; In carbon-halide compounds | M0024-M0036 | 1.0000  (1.0000) | 0.1014  (0.1352) | stg01  stg02  stg03  stg04 | -  stg01  stg02  stg03 |
| 4.1.2 | Lyases; Carbon-carbon lyases; Aldehyde-lyases | M0072-M0217 | 1.0000  (1.0000) | 0.3953  (0.3953) | stg02  stg03 | stg01  stg02 |
|  |  | M0072-M0222 | 0.6667  (1.0000) | 0.1077  (0.5385) | -  -  -  -  stg03REV  -  stg02REV  -  -  - | stg01  stg02  stg03  stg04  stg05  stg06  stg07  stg08  stg09  stg10 |
|  |  | M0217-M0222 | 0.6667  (1.0000) | 0.0687  (0.3435) | -  -  -  -  stg02REV  -  stg01REV  -  -  - | stg01  stg02  stg03  stg04  stg05  stg06  stg07  stg08  stg09  stg10 |
| 4.6.1 | Lyases; Phosphorus-oxygen lyases; Phosphorus-oxygen lyases | M0026-M0058 | 1.0000  (1.0000) | 0.1200  (0.1200) | stg01  stg02 | stg01  stg02 |
| 5.1.1 | Isomerases; Racemases and epimerases; Acting on amino acids and derivates | M0001-M0213 | 1.0000  (1.0000) | 0.0417  (0.0625) | -  -  stg01  stg02  stg03  stg04  -  - | stg01  stg02  stg03  -  -  stg04  stg05  stg06 |
| 6.3.1 | Ligases; Forming carbon-nitrogen bonds; Acid-ammonia (or amine) ligases (amide synthases) | M0075-M0200 | 0.7143  (0.7143) | 0.6311  (0.6311) | **stg01**  stg02  stg03 | **stg01**  stg02  stg03 |
| **Group 2: Sub-subclasses containing highly similar and/or distantly similar plus non-similar overall reactions** | | | | | | |
| 1.1.1 | Oxidoreductases; Acting on the CH-OH group of donors; With NAD+ or NADP+ as acceptor | M0007-M0092 | 0.5000  (0.7000) | 0.2238  (0.3357) | **stg01**  stg02  stg03  -  -  stg04 | **stg01**  stg02  stg03  stg04  stg05  stg06 |
|  |  | M0007-M0093 | 0.5000  (0.7000) | 0.2844  (0.2844) | stg04REV  stg03REV  stg02REV  **stg01REV** | stg04  stg01  stg02  **stg03** |
|  |  | M0092-M0093 | 0.7500  (0.7500) | 0.4493  (0.6739) | stg06REV  stg05REV  stg04REV  **stg03REV**  stg02REV  **stg01REV** | stg04  -  -  **stg01**  stg02  **stg03** |
| 2.3.1 | Transferases; Acyltransferases; Transferring groups other than aminoacyl groups | M0022-M0030 | 0.3333  (0.3333) | 0.0680  (0.1189) | stg04REV  stg03REV  stg02REV  stg01REV  -  -  -  - | -  stg01  stg02  stg03  stg04  stg05  stg06  stg07 |
|  |  | M0022-M0069 | 0.3333  (0.3333) | 0.1842  (0.3684) | stg01  stg02  stg03  stg04 | stg01  stg02  -  - |
|  |  | M0022-M0077 | 0.3333  (0.3333) | 0.1321  (0.1981) | stg04REV  stg03REV  stg02REV  -  -  -  stg01REV | -  stg01  stg02  stg03  stg04  stg05  stg06 |
|  |  | M0030-M0069 | 0.3333  (0.3333) | 0.0843  (0.2952) | stg07REV  stg06REV  stg05REV  stg04REV  stg03REV  stg02REV  stg01REV | -  -  -  -  stg01  stg02  - |
|  |  | M0030-M0077 | 1.0000  (1.0000) | 0.2420  (0.2824) | stg07REV  stg06REV  stg05REV  stg04REV  stg03REV  stg02REV  stg01REV | stg01  stg02  stg03  stg04  stg05  stg06  - |
|  |  | M0069-M0077 | 0.3333  (0.3333) | 0.1765  (0.5294) | -  stg01  -  -  -  **stg02** | stg01  stg02  stg03  stg04  stg05  **stg06** |
| 2.4.2 | Transferases; Glycosyltransferases; Pentosyltransferases | M0008-M0017 | 0.4286  (0.6429) | 0.1739  (0.2174) | stg05REV  stg04REV  stg03REV  stg02REV  stg01REV | stg01  -  stg02  stg03  stg04 |
|  |  | M0008-M0076 | 0.4286  (0.6429) | 0.2353  (0.5882) | **stg05REV**  stg04REV  stg03REV  stg02REV  stg01REV | **stg01**  -  -  stg02  - |
|  |  | M0008-M0079 | 0.1428  (0.4286) | 0.0588  (0.2941) | stg05REV  stg04REV  stg03REV  stg02REV  stg01REV | -  -  stg01  -  - |
|  |  | M0008-M0091 | 0.4286  (0.6429) | 0.2558  (0.3198) | stg05REV  stg04REV  **stg03REV**  stg02REV  stg01REV | stg01  -  **stg02**  stg03  stg04 |
|  |  | M0008-M0214 | 0.3333  (0.3333) | 0.1667  (0.1667) | stg05REV  stg04REV  stg03REV  stg02REV  stg01REV  - | stg01  -  stg02  stg03  stg04  stg05 |
|  |  | M0017-M0076 | 1.0000  (1.0000) | 0.0976  (0.1951) | stg01  stg02  stg03  stg04 | stg01  -  -  stg02 |
|  |  | M0017-M0079 | 0.2000  (0.4000) | 0.0417  (0.1667) | stg01  stg02  stg03  stg04 | -  stg01  -  - |
|  |  | M0017-M0091 | 1.0000  (1.0000) | 0.7778  (0.7778) | **stg01**  **stg02**  stg03  **stg04** | **stg01**  **stg02**  stg03  **stg04** |
|  |  | M0017-M0214 | 0.4286  (0.6429) | 0.1416  (0.1771) | stg01  stg02  stg03  -  stg04 | stg01  stg02  stg03  stg04  stg05 |
|  |  | M0076-M0079 | 0.2000  (0.4000) | 0.0714  (0.1429) | stg01  stg02 | -  stg01 |
|  |  | M0076-M0091 | 1.0000  (1.0000) | 0.0976  (0.1951) | stg01  -  -  stg02 | stg01  stg02  stg03  stg04 |
|  |  | M0076-M0214 | 0.4286  (0.6429) | 0.1667  (0.4167) | -  -  -  -  **stg02REV**  stg01REV | stg01  stg02  stg03  stg04  **stg05**  - |
|  |  | M0079-M0091 | 0.2000  (0.4000) | 0.0714  (0.2857) | -  stg01  -  - | stg01  stg02  stg03  stg04 |
|  |  | M0079-M0214 | 0.3333  (1.0000) | 0.0345  (0.1724) | -  -  -  -  stg01REV | stg01  stg02  stg03  stg04  stg05 |
|  |  | M0091-M0214 | 0.4286  (0.6429) | 0.1538  (0.1923) | stg01  stg02  stg03  -  stg04 | stg01  stg02  stg03  stg04  stg05 |
| 4.1.1 | Lyases; Carbon-carbon lyases; Carboxy-lyases | M0049-M0050 | 1.0000  (1.0000) | 0.0693  (0.2426) | stg07  stg01  stg02  stg03  stg04  stg05  stg06 | -  -  -  -  stg01  -  stg02 |
|  |  | M0049-M0051 | 0.2857  (0.5714) | 0.0800  (0.2800) | stg07  stg01  stg02  stg03  stg04  stg05  stg06 | -  -  -  stg01  -  -  stg02 |
|  |  | M0049-M0070 | 1.0000  (1.0000) | 0.1385  (0.4849) | stg07  stg01  stg02  stg03  stg04  stg05  stg06 | -  -  -  stg01  stg02  -  - |
|  |  | M0049-M0215 | 0.7500  (1.0000) | 0.2165  (0.2526) | stg07  stg01  stg02  stg03  stg04  stg05  stg06 | stg01  -  stg02  stg03  stg04  stg05  stg06 |
|  |  | M0050-M0051 | 0.2857  (0.5714) | 0.0909  (0.0909) | stg01  stg02 | stg01  stg02 |
|  |  | M0050-M0070 | 1.0000  (1.0000) | 0.0909  (0.0909) | stg01  stg02 | stg01  stg02 |
|  |  | M0050-M0215 | 0.7500  (1.0000) | 0.0714  (0.2143) | -  -  stg01  -  -  stg02 | stg01  stg02  stg03  stg04  stg05  stg06 |
|  |  | M0051-M0070 | 0.2857  (0.5714) | 0.3333  (0.3333) | **stg01**  stg02 | **stg01**  stg02 |
|  |  | M0051-M0215 | 0.2500  (0.3750) | 0.0811  (0.2432) | -  -  stg01  -  -  stg02 | stg01  stg02  stg03  stg04  stg05  stg06 |
|  |  | M0070-M0215 | 0.7500  (1.0000) | 0.1189  (0.3566) | -  -  stg01  stg02  -  - | stg01  stg02  stg03  stg04  stg05  stg06 |
| 4.2.1 | Lyases; Carbon-oxygen lyases; Hydro-lyases | M0010-M0054 | 1.0000  (1.0000) | 0.1751  (0.5252) | -  -  **stg03**  -  stg01  stg02  -  -  - | stg01  stg02  **stg03**  stg04  stg05  stg06  stg07  stg08  stg09 |
|  |  | M0010-M0055 | 1.0000  (1.0000) | 0.2101  (0.2101) | stg03  stg01  stg02 | stg03  stg01  stg02 |
|  |  | M0010-M0057 | 0.2500  (0.3750) | 0.1628  (0.2442) | stg02REV  stg01REV  stg03REV | stg01  -  stg02 |
|  |  | M0010-M0073 | 0.3333  (0.3333) | 0.1260  (0.1680) | stg01  stg02  stg03  - | stg01  stg02  stg03  stg04 |
|  |  | M0010-M0204 | 0.2857  (0.3571) | 0.1177  (0.1569) | stg03  stg01  -  stg02 | stg01  stg02  stg03  stg04 |
|  |  | M0010-M0216 | 0.3333  (0.3333) | 0.2883  (0.2883) | stg02REV  stg01REV  **stg03REV**  - | stg01  -  **stg02**  stg03 |
|  |  | M0054-M0055 | 1.0000  (1.0000) | 0.1689  (0.5067) | stg01  stg02  stg03  stg04  stg05  stg06  stg07  stg08  stg09 | -  -  -  stg03  stg01  stg02  -  -  - |
|  |  | M0054-M0057 | 0.2500  (0.3750) | 0.0837  (0.3768) | stg09REV  stg08REV  stg07REV  stg06REV  stg05REV  stg04REV  stg03REV  stg02REV  stg01REV | -  -  -  -  -  stg01  stg02  -  - |
|  |  | M0054-M0073 | 0.3333  (0.3333) | 0.1538  (0.3462) | stg09REV  stg08REV  stg07REV  stg06REV  stg05REV  stg04REV  stg03REV  stg02REV  stg01REV | stg01  -  stg02  -  -  -  stg03  -  stg04 |
|  |  | M0054-M0204 | 0.2857  (0.3571) | 0.0924  (0.2080) | stg01  stg02  stg03  stg04  stg05  stg06  stg07  stg08  stg09 | -  -  -  stg01  stg02  stg03  stg04  -  - |
|  |  | M0054-M0216 | 0.3333  (0.3333) | 0.1260  (0.3780) | stg09REV  stg08REV  stg07REV  stg06REV  stg05REV  stg04REV  **stg03REV**  stg02REV  stg01REV | -  stg01  -  -  -  -  **stg02**  -  stg03 |
|  |  | M0055-M0057 | 0.2500  (0.3750) | 0.1628  (0.2442) | stg03REV  stg02REV  stg01REV | stg01  -  stg02 |
|  |  | M0055-M0073 | 0.3333  (0.3333) | 0.1798  (0.2397) | stg03  stg01  -  stg02 | stg01  stg02  stg03  stg04 |
|  |  | M0055-M0204 | 0.2857  (0.3571) | 0.1325  (0.1767) | stg01  stg02  -  stg03 | stg01  stg02  stg03  stg04 |
|  |  | M0055-M0216 | 0.3333  (0.3333) | 0.1338  (0.1339) | stg02REV  stg01REV  stg03REV | stg01  stg02  stg03 |
|  |  | M0057-M0073 | 0.4286  (0.6429) | 0.1454  (0.2909) | stg02REV  stg01REV  -  - | stg01  stg02  stg03  stg04 |
|  |  | M0057-M0204 | 0.1000  (0.1200) | 0.0761  (0.1522) | stg02REV  -  -  stg01REV | stg01  stg02  stg03  stg04 |
|  |  | M0057-M0216 | 0.2500  (0.3750) | 0.1475  (0.2213) | stg01  stg02  - | stg01  stg02  stg03 |
|  |  | M0073-M0204 | 0.1250  (0.1562) | 0.0971  (0.0971) | stg01  stg02  stg03  stg04  - | -  stg01  stg02  stg03  stg04 |
|  |  | M0073-M0216 | 0.3333  (0.3333) | 0.1150  (0.1534) | stg04REV  stg03REV  stg02REV  stg01REV | stg01  -  stg02  stg03 |
|  |  | M0204-M0216 | 0.1250  (0.1562) | 0.0664  (0.0885) | stg04REV  stg03REV  stg02REV  stg01REV | stg02  -  stg03  stg01 |
| **Group 3: Sub-subclasses containing only non-similar overall reactions** | | | | | | |
| 2.1.1 | Transferases; Transferring one-carbon groups; Methyltransferases | M0031-M0046 | 0.2222  (0.5926) | 0.0874  (0.2621) | stg06REV  stg05REV  stg04REV  stg03REV  stg02REV  stg01REV | -  -  -  -  stg01  stg02 |
| 2.4.1 | Transferases; Glycosyltransferases; Hexosyltransferases | M0045-M0205 | 0.5000  (1.0000) | 0.2353  (0.2353) | stg01  stg02 | stg01  stg02 |
| 3.2.2 | Hydrolases; Glycosylases; Hydrolysing N-glycosyl compounds | M0039-M0071 | 0.4286  (0.6429) | 0.3793  (0.3793) | stg01  stg02 | stg01  stg02 |
| 3.5.4 | Hydrolases; Acting on carbon-nitrogen bonds, other than peptide bonds; In cyclic amidines | M0038-M0097 | 0.3636  (0.4156) | 0.2098  (0.2623) | stg01  stg02  stg03  stg04  stg05 | stg02  stg03  -  stg04  stg01 |
| 4.2.3 | Lyases; Carbon-oxygen lyases; Acting on phosphates | M0059-M0084 | 0.1538  (0.4231) | 0.1474  (0.1769) | -  -  stg01  stg02  stg03  stg04  stg05 | stg01  stg02  stg03  stg04  stg05  -  stg06 |
|  |  | M0059-M0085 | 0.2000  (0.4000) | 0.2021  (0.3369) | stg01  stg02  stg03  stg04  stg05 | -  stg01  -  stg02  stg03 |
|  |  | M0059-M0089 | 0.1176  (0.4412) | 0.1119  (0.1567) | -  stg01  stg02  -  stg03  stg04  stg05 | stg01  stg02  stg03  stg04  stg05  stg06  stg07 |
|  |  | M0084-M0085 | 0.3571  (0.4911) | 0.1297  (0.2594) | stg06REV  stg05REV  stg04REV  stg03REV  stg02REV  stg01REV | -  stg01  -  stg02  -  stg03 |
|  |  | M0084-M0089 | 0.2381  (0.3247) | 0.1211  (0.1413) | -  stg01  stg02  -  stg03  stg04  stg05  stg06 | stg01  stg02  stg03  stg04  stg05  stg06  -  stg07 |
|  |  | M0085-M0089 | 0.1500  (0.2812) | 0.1200  (0.2800) | -  -  stg01  -  -  stg02  stg03 | stg01  stg02  stg03  stg04  stg05  stg06  stg07 |
| 5.4.2 | Isomerases; Intramolecular transferases; Phosphotransferases (phosphomutases) | M0194-M0206 | 0.5000  (1.0000) | 0.1250  (0.2500) | -  stg02REV  stg01REV  - | stg01  stg02  stg03  stg04 |
| 5.4.99 | Isomerases; Intramolecular transferases; Transferring other groups | M0062-M0081 | 0.1111  (0.1667) | 0.0161  (0.0968) | stg06REV  stg05REV  stg04REV  stg03REV  stg02REV  stg01REV | -  -  -  stg01  -  - |

^a^Similarity values after normalization are shown in parenthesis. Scores significant at the 5% level are shown in red.

^b^Alignment of steps that maximizes mechanistic similarity, including reverse reactions and circular permutation of mechanistic steps. If the difference between the Tanimoto coefficients for the mechanistic similarity for forward and reverse alignment of steps was less than 0.001, then the direction of reactions suggested by the EC sub-subclass to which they belong was used. stgXX refers to step XX in the reaction mechanism of an enzyme. REV indicates that the reverse reaction steps were used for the alignment. Identical steps are shown in bold.
