# Supplementary material for: Quantitative Comparison of Catalytic Mechanisms and Overall Reactions in Convergently Evolved Enzymes: Implications for Classification of Enzyme Function
Source: PLoS Comput Biol. 2010 Mar 12;6(3):e1000700. doi: 10.1371/journal.pcbi.1000700 (PMC2837397; doi:10.1371/journal.pcbi.1000700)
Supplement: Table S4 — Summary of overall reaction similarity. (0.03 MB DOC) [file pcbi.1000700.s008.doc]

**Table S4. Summary of overall reaction similarity.**

|  | **Number of sub-subclasses** | **Number of pairs of reactions** | **Average sum of the number of bond changes for pairs of overall reactions** | **Average difference in the number of bond changes for pairs of overall reactions** | **Pairs with significant mechanistic similarity^a^** | **Pairs with identical mechanistic steps^a^** |
| --- | --- | --- | --- | --- | --- | --- |
| **Sub-subclasses containing only similar overall reactions (group 1 in Table 1)** | 17 | 28 | 8.82 | 0.25 | 12  (42.9%) | 9  (32.1%) |
| **Sub-subclasses containing both similar and non-similar overall reactions (group 2 in Table 1)** | 5 | 55 | 9.38 | 1.16 | 6  (10.9%) | 12  (21.8%) |
| **Sub-subclasses containing only non-similar overall reactions (group 3 in Table 1)** | 7 | 12 | 14.33 | 4.17 | 1  (8.3%) | 0  (0%) |

^a^Percentage of pairs as a function of the total number of pairs of reactions for each group is shown in parenthesis.
