# Supplementary material for: Quantitative Comparison of Catalytic Mechanisms and Overall Reactions in Convergently Evolved Enzymes: Implications for Classification of Enzyme Function
Source: PLoS Comput Biol. 2010 Mar 12;6(3):e1000700. doi: 10.1371/journal.pcbi.1000700 (PMC2837397; doi:10.1371/journal.pcbi.1000700)
Supplement: Table S5 — Summary of mechanistic similarity. (0.03 MB DOC) [file pcbi.1000700.s009.doc]

**Table S5. Summary of mechanistic similarity.**

|  | **Number of sub-subclasses** | **Number of pairs of reactions** | **Average sum of the number of mechanistic steps for pairs of reactions** | **Average difference in the number of mechanistic steps for pairs of reactions** | **Pairs with identical mechanistic steps^a^** |
| --- | --- | --- | --- | --- | --- |
| **Sub-subclasses containing only similar mechanisms** | 7 | 9 | 6.11 | 0.78 | 6  (66.7%) |
| **Sub-subclasses containing both similar and non-similar mechanisms** | 7 | 61 | 7.61 | 2.20 | 13  (21.3%) |
| **Sub-subclasses containing only non-similar mechanisms** | 15 | 25 | 9.24 | 2.12 | 2  (8.0%) |

^a^Percentage of pairs as a function of the total number of pairs of reactions for each group is shown in parenthesis.
