# Supplementary material for: Quantitative Comparison of Catalytic Mechanisms and Overall Reactions in Convergently Evolved Enzymes: Implications for Classification of Enzyme Function
Source: PLoS Comput Biol. 2010 Mar 12;6(3):e1000700. doi: 10.1371/journal.pcbi.1000700 (PMC2837397; doi:10.1371/journal.pcbi.1000700)
Supplement: Table S6 — Cases of convergence of active sites in the dataset of functionally analogous enzymes, as identified by Gherardini and colleagues [11]. (0.04 MB DOC) [file pcbi.1000700.s010.doc]

**Table S6. Cases of convergence of active sites in the dataset of functionally analogous enzymes, as identified by Gherardini and colleagues [11]**.

| **EC code** | **Enzyme name** | **MACiE** | **PDB** | **CATH domain^a^** | **Matching catalytic residues** | **Homolog in Gherardini et al.’s work** |
| --- | --- | --- | --- | --- | --- | --- |
| **2.3.1 [2.Transferases; 2.3.Acyltransferases; 2.3.1.Transferring groups other than aminoacyl groups]** | | | | | | |
| 2.3.1.16 | acetyl-CoA C-acyltransferase | M0077 | 1afw | **3.40.47.10**; **3.40.47.20** | Cys125 Cys403 | 1m1t |
| 2.3.1.54 | formate C-acetyltransferase | M0030 | 2pfl | **3.20.70.20** | Cys418 Cys419 | 1mzo |
| 2.3.1.87 | aralkylamine N-acetyltransferase | M0022 | 1b6b | **3.40.630.30** | His122 | 1l0c |
| 2.3.1.129 | acyl-[acyl-carrier-protein]-UDP-N-acetylglucosamine O-acyltransferase | M0069 | 1lxa | 1.20.1180.10; **2.160.10.10** | His125 | 1lxa |
| **3.1.1 [3.Hydrolases; 3.1.Acting on ester bonds; 3.1.1.Carboxylic-ester hydrolases]** | | | | | | |
| 3.1.1.4 | phospholipase A2 | M0083 | 1l8s | **1.20.90.10** | Asp99 His48 water | 1pir |
| 3.1.1.47 | 1-alkyl-2-acetylglycerophosphocholine esterase | M0094 | 1bwp | **3.40.50.1110** | Asp192 His195 Ser47 | 1bwq |
| **3.5.1 [3.Hydrolases; 3.5.Acting on carbon-nitrogen bonds, other than peptide bonds; 3.5.1.In linear amides]** | | | | | | |
| 3.5.1.38 | glutamin-(asparagin-)ase | M0029 | 1djo | **3.40.50.40**; **3.40.50.1170** | Asp1101A Lys1173A Thr1100A | 1hfj |
| 3.5.1.59 | N-carbamoylsarcosine amidase | M0025 | 1nba | **3.40.50.850** | Asp51A Lys144A Cys177A | 1nba |

^a^Catalytic domains are shown in bold.
