# Supplementary material for: Quantitative Comparison of Catalytic Mechanisms and Overall Reactions in Convergently Evolved Enzymes: Implications for Classification of Enzyme Function
Source: PLoS Comput Biol. 2010 Mar 12;6(3):e1000700. doi: 10.1371/journal.pcbi.1000700 (PMC2837397; doi:10.1371/journal.pcbi.1000700)
Supplement: Table S7 — Set of bond changes for every overall reaction and mechanistic step used in this work. (0.09 MB DOC) [file pcbi.1000700.s011.doc]

**Table S7. Set of bond changes for every overall reaction and mechanistic step used in this work.**

Overall bond changes (MXXXX.ov) and step bond changes (MXXXX.stgXX) start after the semicolon and are separated by commas. c: bond cleaved, d: bond decreased in order, f: bond formed, i: bond increased in order, - single bond, = double bond, # triple bond.

**Dataset Overall Bond Changes**

M0001.ov;c:C-H,f:C-H

M0002.ov;c:C-N,c:O-H,f:C-O,f:N-H

M0007.ov;c:C-C,c:C-H,c:O-H,d:C=C,d:C=N,f:C-H,f:C-H,i:C-C,i:C-O,i:C-O

M0008.ov;c:C-C,c:C-O,f:C-H,f:C-N,f:O-H,i:C-O

M0010.ov;c:C-H,c:C-O,f:O-H,i:C-C

M0011.ov;c:O-H,c:P-O,f:O-H,f:P-O

M0016.ov;c:C-N,c:O-H,f:C-O,f:N-H

M0017.ov;c:C-N,c:O-H,f:C-O,f:N-H

M0019.ov;c:C-O,c:O-H,f:C-O,f:O-H

M0020.ov;d:C=C,f:C-H,f:C-H

M0022.ov;c:C-S,c:N-H,f:C-N,f:S-H

M0024.ov;c:C-Cl,c:O-H,f:C-O

M0025.ov;c:C-N,c:O-H,f:C-O,f:N-H

M0026.ov;c:O-H,c:P-O,f:O-H,f:P-O

M0027.ov;c:O-H,c:P-O,f:O-H,f:P-O

M0028.ov;c:O-H,c:P-O,f:O-H,f:P-O

M0029.ov;c:C-N,c:O-H,f:C-O,f:N-H

M0030.ov;c:C-C,c:S-H,f:C-H,f:C-S

M0031.ov;c:C-C,c:C-H,c:C-H,c:C-N,f:C-C,f:C-H,f:N-H,i:C-N

M0036.ov;c:C-Cl,c:O-H,f:C-O

M0038.ov;c:C-C,c:C-O,c:O-H,f:C-C,f:C-O,f:N-H,i:C-O

M0039.ov;c:C-N,c:O-H,f:C-O,f:N-H

M0041.ov;c:O-H,c:P-O,f:O-H,f:P-O

M0043.ov;c:O-H,c:P-O,f:O-H,f:P-O

M0044.ov;c:O-H,c:P-O,f:O-H,f:P-O

M0045.ov;c:C-O,f:C-O

M0046.ov;c:C-S,c:N-H,f:C-N

M0047.ov;c:O-H,c:P-O,f:O-H,f:P-O

M0049.ov;c:C-C,f:C-H,i:C-O

M0050.ov;c:C-C,f:C-H,i:C-O

M0051.ov;c:C-C,c:P-O,d:C=O,f:P-O,i:C-C,i:C-O

M0053.ov;c:C-S,c:O-H,d:C=O,f:C-C,f:C-O,f:O-H,f:S-H

M0054.ov;c:C-H,c:C-O,f:O-H,i:C-C

M0055.ov;c:C-H,c:C-O,f:O-H,i:C-C

M0057.ov;c:O-H,c:O-H,d:C#N,f:C-O,f:N-H,f:N-H

M0058.ov;c:O-H,c:P-O,f:O-H,f:P-O

M0059.ov;c:C-O,c:C-O,f:C-C,i:C-O

M0062.ov;c:C-C,c:C-H,f:C-C,f:C-H

M0066.ov;c:C-H,c:C-N,c:C-O,d:C=O,f:C-H,f:C-N,f:C-O,i:C-O

M0068.ov;c:C-H,c:C-H,i:C-C

M0069.ov;c:C-S,c:O-H,f:C-O,f:S-H

M0070.ov;c:C-C,f:C-H,i:C-O

M0071.ov;c:C-N,c:O-H,d:C=O,f:C-O,f:O-H,i:C-N

M0072.ov;c:C-C,c:O-H,f:C-H,i:C-O

M0073.ov;c:C-O,c:N-H,f:O-H,i:C-N

M0075.ov;c:C-O,c:N-H,c:P-O,f:C-N,f:O-H,f:P-O

M0076.ov;c:C-N,c:O-H,f:C-O,f:N-H

M0077.ov;c:C-H,c:C-S,f:C-C,f:S-H

M0078.ov;c:C-H,c:C-S,c:O-H,d:C=O,f:C-C,f:C-O,f:O-H,f:S-H

M0079.ov;c:C-N,f:C-N

M0081.ov;c:C-O,d:C=C,d:C=C,f:C-C,i:C-C,i:C-O

M0082.ov;c:C-H,c:C-N,c:C-O,f:C-H,f:C-N,f:C-O

M0083.ov;c:C-O,c:O-H,f:C-O,f:O-H

M0084.ov;c:C-H,c:C-H,c:C-O,c:O-H,c:O-H,f:C-H,f:C-H,f:N-H,f:O-H,i:C-O,i:C-O

M0085.ov;c:C-H,c:C-O,c:O-H,d:C=O,f:O-H,f:O-H,i:C-C,i:C-O

M0087.ov;c:C-N,c:O-H,f:C-O,f:N-H

M0089.ov;c:C-H,c:C-H,c:C-H,c:C-O,d:C=C,d:C=C,d:C=C,d:C=C,f:C-C,f:C-C,f:C-C,f:C-H,f:C-H,i:C-C,i:C-C

M0091.ov;c:C-N,c:O-H,f:C-O,f:N-H

M0092.ov;c:C-H,c:C-H,c:O-H,c:O-H,d:C=C,d:C=C,d:C=N,d:C=N,f:C-H,f:C-H,f:C-O,i:C-C,i:C-C,i:C-O

M0093.ov;c:C-H,c:C-H,c:C-S,d:C=C,d:C=C,d:C=O,f:C-H,f:C-H,f:O-H,f:S-H,i:C-C,i:C-C,i:C-N,i:C-N

M0094.ov;c:C-O,c:O-H,f:C-O,f:O-H

M0097.ov;c:C-N,c:O-H,c:O-H,d:C=N,f:C-O,f:N-H,f:N-H,i:C-O

M0098.ov;c:C-N,c:O-H,f:C-O,f:N-H

M0112.ov;c:C-H,d:C=C,d:C=N,f:C-H,f:N-H,i:C-C,i:C-N

M0120.ov;c:C-H,c:C-N,d:C=C,f:C-H,f:N-H,i:C-C,i:C-N

M0148.ov;c:C-C,c:O-H,d:C=O,f:C-C,f:O-H,i:C-O

M0194.ov;c:P-O,f:P-O

M0200.ov;c:C-O,c:N-H,c:N-H,c:P-O,f:C-N,f:P-O

M0203.ov;c:C-O,c:O-H,f:C-O,f:O-H

M0204.ov;c:C-C,c:C-H,c:C-O,f:C-C,f:C-C

M0205.ov;c:C-O,c:O-H,f:C-O,f:O-H

M0206.ov;c:O-H,c:P-O,f:O-H,f:P-O

M0213.ov;c:C-H,f:C-H

M0214.ov;c:C-N,c:C-O,c:O-H,f:C-N,f:C-O,f:O-H

M0215.ov;c:C-C,c:O-H,f:C-H,i:C-O

M0216.ov;c:O-H,d:C=O,f:C-O,f:O-H

M0217.ov;c:C-C,c:O-H,f:C-H,i:C-O

M0218.ov;c:C-O,c:O-H,f:C-O,f:O-H

M0219.ov;c:C-C,c:O-H,d:C=O,f:C-C,f:O-H,i:C-O

M0222.ov;c:C-H,d:C=O,d:C=O,f:C-C,f:C-O,f:O-H

**Background Overall Bond Changes**

M0003.ov;c:C-H,d:C=C,d:C=C,d:C=O,d:C=O,f:O-H,f:O-H,i:C-C,i:C-C,i:C-C,i:C-N

M0009.ov;c:C-O,c:O-H,c:P-O,f:C-S,f:O-H,f:P-O

M0012.ov;c:C-O,c:N-H,c:N-H,f:C-N,f:O-H

M0013.ov;c:C-H,c:C-N,c:O-H,c:O-H,f:C-O,f:N-H,f:O-H,f:O-H,i:C-O

M0014.ov;c:O-O,f:O-Cl,f:O-H

M0018.ov;c:C-N,c:N-H,f:C-N,f:N-H

M0023.ov;c:C-S,c:N-H,f:C-N

M0032.ov;c:C-H,c:O-H,d:C=O,f:C-H,f:O-H,i:C-O

M0035.ov;c:O-H,c:P-O,f:O-H,f:P-O

M0037.ov;c:C-H,c:O-O,d:C=C,d:C=C,d:C=C,d:O=O,d:O=O,f:C-C,f:C-O,f:C-O,f:C-O,f:O-H,f:O-H,f:O-H,i:C-C

M0040.ov;c:P-O,f:P-O

M0042.ov;c:O-H,c:P-O,f:O-H,f:P-O

M0048.ov;c:C-O,c:N-H,f:C-N

M0052.ov;c:C-H,d:C=O,f:C-C,f:O-H

M0060.ov;c:C-H,c:C-N,c:C-O,c:O-H,c:O-H,f:C-H,f:C-O,f:N-H,f:O-H,i:C-O

M0063.ov;c:C-C,c:C-H,f:C-C,f:C-H

M0065.ov;c:C-O,c:N-H,c:P-O,d:C=O,f:C-N,f:O-H,f:P-O

M0080.ov;c:C-H,c:C-N,f:N-H,i:C-C

M0086.ov;c:N-H,c:P-O,f:O-H,f:P-N

M0088.ov;c:P-O,f:P-O

M0095.ov;c:C-H,c:C-O,c:O-H,f:C-H,f:O-H,i:C-O

M0096.ov;c:C-N,c:O-H,c:O-H,d:C=N,f:C-O,f:N-H,i:C-O

M0099.ov;c:C-H,c:O-H,d:C=O,d:C=O,f:O-H,f:O-H,i:C-C,i:C-O

M0100.ov;c:C-H,c:O-H,d:C=C,d:C=N,f:C-H,f:C-O,i:C-C

M0104.ov;c:C-H,c:O-H,d:C=C,d:C=O,d:C=O,f:O-H,f:O-H,i:C-C,i:C-C,i:C-O

M0118.ov;c:N-H,c:O-H,d:C=C,d:C=N,d:C=O,d:O=O,f:C-O,f:O-H,f:O-H,i:C-N,i:C-N,i:C-O

M0121.ov;c:O-H,c:O-H,f:S-O,i:S-O

M0122.ov;c:S-H,c:S-H,c:S-O,d:S=O,f:O-H,f:O-H,f:S-S

M0124.ov;c:O-O,d:O=O,f:O-H,f:O-H,f:O-H,f:O-H

M0125.ov;c:O-H,c:O-H,c:O-H,c:O-H,c:O-O,d:C=C,d:C=C,d:O=O,f:O-H,f:O-H,f:O-H,f:O-H,i:C-O,i:C-O,i:C-O,i:C-O

M0126.ov;c:H-H,c:H-H

M0127.ov;f:H-H

M0128.ov;c:C-C,c:C-H,c:O-O,c:P-O,d:O=O,f:C-O,f:O-H,f:P-O,i:C-O

M0130.ov;c:C-H,c:O-O,d:C=C,d:C=C,d:O=O,f:C-O,f:C-O,f:O-H,f:O-H,i:C-C,i:C-N

M0132.ov;c:C-H,c:N-H,c:O-O,d:C=C,d:O=O,f:C-O,f:O-H,f:O-H,f:O-H,i:C-N,i:C-N

M0133.ov;c:C-H,c:O-O,d:O=O,f:C-O,f:O-H,f:O-H,f:O-H

M0134.ov;c:C-H,c:O-O,d:C=C,d:C=N,d:O=O,f:C-O,f:C-O,f:O-H,f:O-H,i:C-N,i:C-N

M0135.ov;c:C-H,c:O-O,d:O=O,f:C-O,f:O-H,f:O-H

M0136.ov;c:C-H,c:C-H,c:O-O,d:O=O,f:O-H,f:O-H,f:O-H,f:O-H,i:C-C

M0137.ov;c:C-C,c:C-H,c:C-H,c:C-S,c:O-O,d:O=O,f:C-O,f:C-S,f:O-H,f:O-H,i:C-C,i:C-O

M0138.ov;d:O=O,f:O-H,f:O-H

M0140.ov;c:S-H,c:S-H,f:C-H,f:O-H,f:S-S

M0141.ov;c:C-H,c:C-H,c:O-H,c:O-H,f:C-O,i:C-O

M0142.ov;c:C-H,d:C=C,i:C-C,i:C-N

M0143.ov;c:As-O,d:As=O,f:O-H,f:S-S

M0147.ov;c:C-C,c:C-O,c:N-H,f:C-H,f:C-N,f:C-N,f:O-H

M0149.ov;c:C-N,c:N-H,f:C-N,f:N-H

M0150.ov;c:P-O,f:P-O

M0151.ov;c:O-H,c:P-O,f:P-O

M0152.ov;c:O-H,c:P-O,f:O-H,f:P-O

M0153.ov;c:S-S,f:C-S

M0154.ov;c:O-H,c:S-O,f:O-H,f:S-O

M0156.ov;c:C-S,c:S-H,f:C-H,f:S-S

M0157.ov;c:C-S,c:O-H,f:C-O,f:S-H

M0158.ov;c:O-H,c:S-O,f:O-H,f:S-O

M0160.ov;c:O-H,c:P-O,f:O-H,f:P-O

M0162.ov;c:C-H,c:C-N,c:C-O,c:C-O,c:O-H,c:O-H,f:C-O,f:N-H,f:O-H,i:C-C,i:C-O

M0163.ov;c:O-H,c:P-O,f:O-H,f:P-O

M0164.ov;c:O-H,c:P-O,f:O-H,f:P-O

M0165.ov;c:O-H,c:P-O,f:O-H,f:P-O

M0167.ov;c:C-N,c:O-H,f:C-O,f:N-H

M0169.ov;c:C-N,c:O-H,f:C-O,f:N-H

M0172.ov;c:C-N,c:O-H,f:C-O,f:N-H

M0173.ov;c:C-N,c:O-H,f:C-O,f:N-H

M0174.ov;c:C-N,c:O-H,f:C-O,f:N-H

M0175.ov;c:C-N,c:O-H,f:C-O,f:N-H

M0176.ov;c:C-N,c:O-H,f:C-O,f:N-H

M0177.ov;c:C-N,c:O-H,f:C-O,f:N-H

M0179.ov;c:O-H,c:P-O,f:O-H,f:P-O

M0180.ov;c:C-C,c:O-H,f:C-H,f:C-O

M0181.ov;c:C-P,c:O-H,f:C-H,f:P-O

M0183.ov;c:C-C,c:C-C,i:C-C,i:C-C

M0184.ov;c:C-H,c:C-O,f:O-H,i:C-C

M0185.ov;c:C-H,c:C-N,c:C-O,c:C-O,c:O-H,f:C-O,f:N-H,f:O-H,i:C-C,i:C-O

M0186.ov;c:C-H,c:C-O,f:O-H,i:C-C

M0187.ov;c:C-H,f:C-H

M0190.ov;c:C-H,d:C=C,f:C-H,i:C-C

M0191.ov;c:S-S,c:S-S,f:S-S,f:S-S

M0196.ov;d:C=C,f:C-H,f:C-O

M0202.ov;c:O-H,c:P-O,c:P-O,f:O-H,f:P-O,f:P-O

M0207.ov;c:C-H,c:P-O,c:P-O,d:C=O,f:P-O,f:P-O,i:C-C

M0209.ov;c:O-H,c:P-O,f:P-O

M0210.ov;c:C-N,c:O-H,f:C-O,f:N-H

M0211.ov;c:C-H,c:C-H,c:N-O,d:C=C,d:C=C,d:N=O,f:N-H,f:O-H,f:O-H,f:O-H,i:C-C,i:C-C,i:C-N,i:C-N

M0220.ov;c:C-C,c:O-H,f:C-H,i:C-O

**Dataset Step Bond Changes**

M0001.stg01;c:S-H,f:O-H

M0001.stg02;c:C-H,d:C=O,f:S-H,i:C-C

M0001.stg03;c:S-H,d:C=C,f:C-H,i:C-O

M0001.stg04;c:O-H,f:S-H

M0002.stg01;c:O-H,d:C=O,f:C-O,f:N-H

M0002.stg02;c:C-N,c:O-H,f:N-H,i:C-O

M0002.stg03;c:N-H,f:O-H

M0002.stg04;c:O-H,d:C=O,f:C-O,f:O-H

M0002.stg05;c:C-O,c:O-H,f:O-H,i:C-O

M0007.stg01;c:C-H,c:O-H,d:C=C,d:C=N,f:C-H,f:O-H,i:C-C,i:C-O

M0007.stg02;c:C-C,d:C=O,i:C-C,i:C-O

M0007.stg03;c:N-H,d:C=C,f:C-H,i:C-O

M0007.stg04;c:O-H,f:N-H

M0008.stg01;c:O-H,f:O-H

M0008.stg02;c:C-O,c:R-H,f:O-H,i:C-O

M0008.stg03;d:C=O,f:C-N

M0008.stg04;c:C-C,c:R-H,f:C-H,i:C-O

M0008.stg05;c:O-H,f:O-H

M0010.stg01;c:C-H,d:C=O,f:N-H,i:C-C

M0010.stg02;c:C-O,c:O-H,d:C=C,f:O-H,i:C-C,i:C-O

M0010.stg03;c:N-H,f:O-H

M0011.stg01;c:P-O,f:P-O

M0016.stg01;d:C=O,f:C-O

M0016.stg02;c:O-H,f:O-H

M0016.stg03;c:C-N,c:O-H,f:N-H,i:C-O

M0016.stg04;c:O-H,f:O-H

M0017.stg01;c:O-H,f:N-H

M0017.stg02;c:C-N,d:C=N,i:C-N,i:C-O

M0017.stg03;d:C=O,f:C-O

M0017.stg04;c:N-H,f:N-H

M0019.stg01;c:C-O,c:O-H,c:O-H,c:O-H,f:C-O,f:O-H,f:O-H,f:O-H

M0019.stg02;c:O-H,f:O-H

M0020.stg01;c:R-H,d:C=N,d:C=N,f:N-H,i:C-C

M0020.stg02;c:N-H,c:N-H,d:C=C,d:C=C,d:C=O,f:C-H,f:O-H,i:C-C,i:C-N,i:C-N

M0020.stg03;c:O-H,c:O-H,d:C=C,f:C-H,f:N-H,i:C-O

M0022.stg01;c:N-H,d:C=O,f:C-N,f:N-H

M0022.stg02;c:C-S,c:O-H,f:S-H,i:C-O

M0022.stg03;c:N-H,f:O-H

M0022.stg04;c:N-H,f:O-H

M0024.stg01;c:O-H,d:C=C,d:C=C,d:C=O,f:C-O,f:N-H,i:C-C,i:C-C

M0024.stg02;c:C-Cl,c:N-H,d:C=C,d:C=C,f:H-Cl,i:C-C,i:C-C,i:C-O

M0024.stg03;c:O-H,d:C=O,f:C-O,f:N-H

M0024.stg04;c:C-O,c:N-H,f:O-H,i:C-O

M0025.stg01;c:S-H,d:C=O,f:C-S,f:O-H

M0025.stg02;c:C-N,c:O-H,f:N-H,i:C-O

M0025.stg03;c:O-H,d:C=O,f:C-O,f:O-H

M0025.stg04;c:C-S,c:O-H,f:S-H,i:C-O

M0026.stg01;c:N-H,c:O-H,c:P-O,f:N-H,f:O-H,f:P-O

M0026.stg02;c:N-H,f:N-H

M0027.stg01;c:O-H,c:P-O,f:O-H,f:P-O

M0027.stg02;c:O-H,f:O-H

M0028.stg01;c:N-H,c:O-H,c:P-O,f:O-H,f:O-H,f:P-O

M0028.stg02;c:O-H,c:O-H,c:P-O,f:N-H,f:O-H,f:P-O

M0029.stg01;c:O-H,d:C=O,f:C-O,f:O-H

M0029.stg02;c:C-N,c:O-H,f:N-H,i:C-O

M0029.stg03;c:O-H,d:C=O,f:C-O,f:O-H

M0029.stg04;c:C-O,c:O-H,f:O-H,i:C-O

M0030.stg01;c:S-H,f:C-H

M0030.stg02;d:C=O,f:C-S

M0030.stg03;c:C-C,i:C-O

M0030.stg04;c:S-H,f:C-H

M0030.stg05;c:S-H,f:S-H

M0030.stg06;c:C-S,c:S-H,f:C-S,f:S-H

M0030.stg07;c:C-H,f:S-H

M0031.stg01;c:C-N,c:O-H,f:N-H,i:C-N

M0031.stg02;c:O-H,d:C=C,d:C=O,f:C-S,f:O-H,i:C-C

M0031.stg03;c:O-H,d:C=C,d:C=N,f:C-C,f:O-H,i:C-O

M0031.stg04;c:C-H,c:O-H,d:C=O,f:O-H,f:O-H,i:C-C

M0031.stg05;c:C-N,c:O-H,d:C=C,f:O-H,i:C-C,i:C-O

M0031.stg06;c:C-H,c:C-S,d:C=C,f:C-H,i:C-C,i:C-N

M0036.stg01;c:C-Cl,f:C-O

M0036.stg02;c:C-O,c:O-H,f:C-O,f:O-H

M0036.stg03;c:O-H,f:O-H

M0038.stg01;c:N-H,d:C=N,f:C-O,f:N-H

M0038.stg02;c:C-N,c:C-O,c:N-H,c:O-H,f:N-H,f:O-H,i:C-N,i:C-O

M0038.stg03;c:N-H,c:O-H,d:C=N,f:C-O,f:N-H,f:N-H

M0038.stg04;c:C-N,c:O-H,f:C-O,f:N-H

M0038.stg05;c:C-H,c:C-O,c:C-O,c:N-H,f:C-H,f:C-N,f:O-H,i:C-N

M0039.stg01;c:C-N,c:N-H,c:O-H,d:C=N,f:C-O,f:N-H,f:O-H,i:C-N

M0039.stg02;c:O-H,c:O-H,f:N-H,f:O-H

M0041.stg01;c:N-H,c:O-H,c:P-O,f:N-H,f:O-H,f:P-O

M0041.stg02;c:N-H,f:N-H

M0043.stg01;c:Fe-O,d:P=O,f:P-O

M0043.stg02;c:N-H,c:P-O,f:O-H,i:P-O

M0043.stg03;c:O-H,f:Fe-O,f:N-H

M0044.stg01;c:O-H,f:O-H

M0044.stg02;c:P-O,f:P-O

M0044.stg03;c:O-H,c:P-O,f:O-H,f:P-O

M0044.stg04;c:O-H,f:O-H

M0045.stg01;c:C-O,c:O-H,f:C-O,f:O-H

M0045.stg02;c:C-O,c:O-H,f:C-O,f:O-H

M0046.stg01;c:C-S,f:C-N

M0046.stg02;c:N-H,f:O-H

M0047.stg01;c:O-H,c:P-O,f:O-H,f:P-S

M0047.stg02;c:O-H,c:P-S,f:O-H,f:P-O

M0047.stg03;c:O-H,f:O-H

M0049.stg01;c:N-H,c:O-H,d:C=O,f:C-N,f:O-H,f:O-H

M0049.stg02;c:C-O,c:N-H,c:O-H,f:O-H,f:O-H,i:C-N

M0049.stg03;c:C-C,d:C=N,d:C=O,i:C-C,i:C-N,i:C-O

M0049.stg04;c:O-H,d:C=C,d:C=N,f:C-H,i:C-N,i:C-O

M0049.stg05;c:O-H,c:O-H,d:C=N,f:C-O,f:N-H,f:O-H

M0049.stg06;c:C-N,c:O-H,c:O-H,f:N-H,f:O-H,i:C-O

M0049.stg07;c:O-H,f:O-H

M0050.stg01;c:C-C,c:N-H,f:C-H,i:C-O

M0050.stg02;c:O-H,f:N-H

M0051.stg01;c:C-C,d:C=O,i:C-C,i:C-O

M0051.stg02;c:P-O,f:P-O

M0053.stg01;c:C-H,d:C=O,f:O-H,i:C-C

M0053.stg02;d:C=C,d:C=O,f:C-C,i:C-O

M0053.stg03;c:C-S,c:O-H,c:O-H,f:C-O,f:O-H,f:S-H

M0054.stg01;c:N-H,c:N-H,d:C=N,f:N-H,f:O-H,i:C-N

M0054.stg02;d:C=O,f:C-N

M0054.stg03;c:N-H,f:O-H

M0054.stg04;c:C-O,c:N-H,c:O-H,d:C=N,f:N-H,f:O-H,i:C-N,i:C-N

M0054.stg05;c:C-H,c:N-H,d:C=N,d:C=N,f:N-H,f:O-H,i:C-C,i:C-N

M0054.stg06;c:C-O,c:N-H,c:O-H,d:C=C,d:C=N,f:N-H,f:O-H,i:C-C,i:C-N,i:C-N

M0054.stg07;c:O-H,d:C=N,f:C-O,f:O-H

M0054.stg08;c:C-N,c:O-H,f:N-H,i:C-O

M0054.stg09;c:N-H,c:O-H,d:C=N,f:N-H,f:N-H,i:C-N

M0055.stg01;c:C-H,c:N-H,d:C=O,d:C=O,f:O-H,f:O-H,i:C-C,i:C-N

M0055.stg02;c:C-O,c:N-H,c:O-H,c:O-H,c:O-H,d:C=C,d:C=N,d:C=N,f:N-H,f:N-H,f:O-H,f:O-H,i:C-C,i:C-N,i:C-O,i:C-O

M0055.stg03;c:N-H,c:O-H,d:C=N,f:N-H,f:O-H,i:C-N

M0057.stg01;c:Fe-O,c:O-H,d:C#N,f:C-O,f:Fe-O,f:N-H

M0057.stg02;c:O-H,d:C=N,f:N-H,i:C-O

M0058.stg01;c:O-H,d:P=O,f:O-H,f:P-O

M0058.stg02;c:P-O,i:P-O

M0059.stg01;c:C-H,c:N-H,c:O-H,d:C=C,d:C=N,d:C=N,f:C-H,f:N-H,f:O-H,i:C-C,i:C-N,i:C-O

M0059.stg02;c:C-H,c:C-O,f:O-H,i:C-C

M0059.stg03;c:C-H,c:N-H,c:O-H,d:C=C,d:C=N,d:C=O,f:C-H,f:N-H,f:O-H,i:C-C,i:C-N,i:C-N

M0059.stg04;c:C-O,c:O-H,f:N-H,i:C-O

M0059.stg05;c:N-H,d:C=C,d:C=O,f:C-C,f:O-H,i:C-O

M0062.stg01;c:Co-C,f:Co-N

M0062.stg02;c:C-H,f:C-H

M0062.stg03;c:N-H,d:C=O,f:C-C,f:O-H

M0062.stg04;c:C-C,c:O-H,f:N-H,i:C-O

M0062.stg05;c:C-H,f:C-H

M0062.stg06;c:Co-N,f:Co-C

M0066.stg01;d:C=N,f:C-N

M0066.stg02;c:C-N,c:N-H,f:N-H,i:C-N

M0066.stg03;c:C-H,d:C=C,d:C=N,d:C=N,f:N-H,i:C-C,i:C-C,i:C-N

M0066.stg04;c:N-H,d:C=C,d:C=C,f:C-H,i:C-C,i:C-N

M0066.stg05;c:O-H,d:C=N,f:C-O,f:N-H

M0066.stg06;c:C-N,c:O-H,f:N-H,i:C-O

M0066.stg07;c:N-H,d:C=O,f:C-N,f:O-H

M0066.stg08;c:C-O,c:N-H,f:O-H,i:C-N

M0066.stg09;c:C-H,d:C=C,d:C=N,f:N-H,i:C-C,i:C-C

M0066.stg10;c:N-H,d:C=C,d:C=C,d:C=N,f:C-H,i:C-C,i:C-N,i:C-N

M0066.stg11;c:N-H,d:C=N,f:C-N

M0066.stg12;c:C-N,i:C-N

M0068.stg01;c:C-H,c:C-H,d:C=N,d:C=N,d:C=O,f:N-H,f:O-H,i:C-C,i:C-C,i:C-N

M0068.stg02;d:C=C,d:C=N,i:C-N,i:C-O

M0068.stg03;c:N-H,i:C-N

M0068.stg04;c:O-H,f:O-H

M0069.stg01;c:O-H,d:C=O,f:C-O,f:N-H

M0069.stg02;c:C-S,c:N-H,f:S-H,i:C-O

M0070.stg01;c:C-C,d:C=O,i:C-C,i:C-O

M0070.stg02;c:R-H,d:C=C,f:C-H,i:C-O

M0071.stg01;c:C-N,c:N-H,c:O-H,d:C=O,f:C-O,f:O-H,f:O-H,i:C-N

M0071.stg02;c:O-H,f:N-H

M0072.stg02;c:C-C,c:O-H,f:O-H,i:C-O

M0072.stg03;c:O-H,f:C-H

M0073.stg01;c:N-H,d:C=N,d:C=N,d:C=O,f:N-H,i:C-N,i:C-N,i:C-N

M0073.stg02;c:C-O,c:N-H,f:O-H,i:C-N

M0073.stg03;c:N-H,f:N-H

M0073.stg04;c:N-H,d:C=N,d:C=N,f:N-H,i:C-N,i:C-N,i:C-N,i:C-O

M0075.stg01;c:P-O,f:P-O

M0075.stg02;c:N-H,d:C=O,f:C-N,f:O-H

M0075.stg03;c:C-O,c:O-H,f:O-H,i:C-O

M0076.stg01;c:O-H,f:O-H

M0076.stg02;c:C-N,c:O-H,f:C-O,f:N-H

M0077.stg01;c:S-H,f:N-H

M0077.stg02;d:C=O,f:C-S

M0077.stg03;c:C-S,c:S-H,f:S-H,i:C-O

M0077.stg04;c:C-H,d:C=O,f:S-H,i:C-C

M0077.stg05;d:C=C,d:C=O,f:C-C,i:C-O

M0077.stg06;c:C-S,c:N-H,f:S-H,i:C-O

M0078.stg01;c:C-H,d:C=O,f:O-H,i:C-C

M0078.stg02;c:N-H,d:C=C,d:C=O,f:C-C,f:O-H,i:C-O

M0078.stg03;c:C-S,c:O-H,c:O-H,f:C-O,f:N-H,f:S-H

M0079.stg01;c:C-N,f:C-N

M0081.stg01;c:C-O,d:C=C,d:C=C,f:C-C,i:C-C,i:C-O

M0082.stg01;c:S-H,d:C=O,f:C-S,f:N-H

M0082.stg02;c:C-N,c:N-H,f:N-H,i:C-O

M0082.stg03;c:O-H,d:C=O,f:C-O,f:N-H

M0082.stg04;c:C-S,c:N-H,f:S-H,i:C-O

M0082.stg05;c:C-O,c:O-H,f:N-H,i:C-O

M0082.stg06;c:N-H,f:O-H

M0082.stg07;c:N-H,d:C=O,f:C-N,f:O-H

M0082.stg08;c:C-O,c:N-H,f:O-H,i:C-N

M0082.stg09;c:N-H,d:C=N,f:C-N,f:N-H

M0082.stg10;c:C-N,c:N-H,f:N-H,i:C-N

M0082.stg11;c:C-H,c:N-H,d:C=N,f:N-H,f:O-H,i:C-C

M0082.stg12;c:O-H,c:O-H,d:C=C,f:C-H,f:N-H,i:C-O

M0082.stg13;c:O-H,f:N-H

M0082.stg14;c:N-H,d:C=O,f:C-O,f:O-H

M0083.stg01;c:O-H,d:C=O,f:C-O,f:N-H

M0083.stg02;c:C-O,c:N-H,f:O-H,i:C-O

M0084.stg01;c:O-H,c:S-H,f:N-H,f:N-H

M0084.stg02;c:C-H,d:C=N,f:S-H,i:C-C

M0084.stg03;c:O-H,c:S-H,d:C=C,f:C-H,f:O-H,i:C-O

M0084.stg04;c:C-H,c:C-O,f:S-H,i:C-C

M0084.stg05;c:O-H,c:S-H,d:C=C,f:C-H,f:O-H,i:C-O

M0084.stg06;c:N-H,f:S-H

M0085.stg01;c:C-H,c:N-H,d:C=O,f:O-H,f:O-H,i:C-C

M0085.stg02;c:C-O,c:O-H,d:C=C,f:N-H,i:C-C,i:C-O

M0085.stg03;c:N-H,c:O-H,f:N-H,f:O-H

M0087.stg01;c:O-H,d:C=N,f:C-O,f:N-H

M0087.stg02;c:C-N,c:N-H,c:Ni-O,f:N-H,i:C-O

M0089.stg01;c:C-O

M0089.stg02;d:C=C,f:C-C

M0089.stg03;c:C-H,f:N-H,i:C-C

M0089.stg04;c:N-H,d:C=C,d:C=C,f:C-C,f:C-H

M0089.stg05;c:C-H,d:C=C,f:C-C,f:C-H

M0089.stg06;c:C-H,f:N-H,i:C-C

M0089.stg07;c:N-H,f:O-H

M0091.stg01;c:O-H,f:N-H

M0091.stg02;c:C-N,i:C-O

M0091.stg03;d:C=O,f:C-O

M0091.stg04;c:N-H,f:N-H

M0092.stg01;c:C-H,c:O-H,d:C=C,d:C=N,f:C-H,f:O-H,i:C-C,i:C-O

M0092.stg02;d:C=O,f:C-S

M0092.stg03;c:C-H,d:C=C,d:C=N,f:C-H,i:C-C,i:C-O

M0092.stg04;c:O-H,d:C=O,f:C-O,f:O-H

M0092.stg05;c:C-S,i:C-O

M0092.stg06;c:O-H,c:O-H,f:O-H,f:O-H

M0093.stg01;c:C-H,d:C=C,d:C=O,f:C-H,i:C-C,i:C-N

M0093.stg02;c:C-S,c:N-H,f:S-H,i:C-O

M0093.stg03;c:C-H,c:O-H,d:C=C,d:C=O,f:C-H,f:O-H,i:C-C,i:C-N

M0093.stg04;c:O-H,c:O-H,f:N-H,f:O-H

M0094.stg01;c:O-H,f:N-H

M0094.stg02;d:C=O,f:C-O

M0094.stg03;c:C-O,c:N-H,f:O-H,i:C-O

M0094.stg04;c:O-H,d:C=O,f:C-O,f:N-H

M0094.stg05;c:C-O,c:N-H,f:O-H,i:C-O

M0097.stg01;c:O-H,f:O-H

M0097.stg02;c:O-H,d:C=N,f:C-O,f:N-H

M0097.stg03;c:O-H,f:O-H

M0097.stg04;c:C-N,c:O-H,f:N-H,i:C-O

M0098.stg01;c:O-H,f:O-H

M0098.stg02;d:C=O,f:C-O

M0098.stg03;c:C-N,c:Fe-O,c:O-H,f:Fe-O,f:N-H,i:C-O

M0112.stg01;c:O-H,d:C=O,f:O-H,i:C-N

M0112.stg02;c:C-H,c:O-H,d:C=C,d:C=N,d:C=N,f:C-H,f:N-H,i:C-C,i:C-N,i:C-O

M0120.stg01;c:C-H,c:O-H,d:C=C,d:C=N,d:C=N,f:N-H,f:N-H,i:C-C,i:C-C,i:C-N

M0120.stg02;c:C-N,c:N-H,c:O-H,c:O-H,c:O-H,d:C=N,d:C=O,f:N-H,f:N-H,f:O-H,f:O-H,f:O-H,i:C-N,i:C-N,i:C-O

M0120.stg03;c:N-H,c:N-H,d:C=C,d:C=N,f:C-H,f:O-H,i:C-N,i:C-N

M0148.stg01;c:N-H,f:O-H

M0148.stg02;c:N-H,d:C=O,f:C-N,f:O-H

M0148.stg03;c:C-O,c:O-H,f:O-H,i:C-N

M0148.stg04;c:C-C,c:O-H,f:O-H,i:C-O

M0148.stg05;d:C=N,i:C-C

M0148.stg06;c:O-H,d:C=C,d:C=O,f:C-C,f:O-H,i:C-N

M0148.stg07;c:O-H,d:C=N,f:C-O,f:O-H

M0148.stg08;c:C-N,c:O-H,f:N-H,i:C-O

M0148.stg09;c:O-H,f:N-H

M0194.stg01;c:P-O,f:P-O

M0194.stg02;c:P-O,f:P-O

M0200.stg01;c:P-O,f:P-O

M0200.stg02;c:N-H,d:C=O,f:O-H

M0200.stg03;c:C-O,c:N-H,c:O-H,f:C-N,f:O-H,f:O-H,i:C-O

M0203.stg01;c:C-O,c:O-H,f:C-O,f:O-H

M0203.stg02;c:C-O,c:O-H,f:C-O,f:O-H

M0204.stg01;c:C-O,c:N-H,d:C=C,d:C=C,f:C-C,f:O-H,i:C-C,i:C-N

M0204.stg02;c:C-C,c:O-H,d:C=C,d:C=C,d:C=C,d:C=N,f:N-H,i:C-C,i:C-C,i:C-C,i:C-N

M0204.stg03;d:C=C,d:C=C,d:C=C,d:C=C,d:C=N,f:C-C,i:C-C,i:C-C,i:C-C,i:C-N

M0204.stg04;c:C-H,d:C=C,d:C=N,f:O-H,i:C-C,i:C-C

M0205.stg01;c:C-O,c:O-H,c:O-H,d:P=O,f:O-H,f:O-H,i:C-O,i:P-O

M0205.stg02;c:O-H,d:C=O,d:P=O,f:C-O,f:O-H,i:P-O

M0206.stg01;c:O-H,d:P=O,f:O-H,f:P-O

M0206.stg02;c:P-O,i:P-O

M0206.stg03;d:P=O,f:P-O

M0206.stg04;c:O-H,c:P-O,f:O-H,i:P-O

M0213.stg01;d:C=N,f:C-N

M0213.stg02;c:C-N,c:N-H,f:N-H,i:C-N

M0213.stg03;c:C-H,c:O-H,f:N-H,f:O-H

M0213.stg04;c:C-H,c:O-H,f:N-H,f:O-H

M0213.stg05;d:C=N,f:C-N

M0213.stg06;c:C-N,c:N-H,f:N-H,i:C-N

M0214.stg01;c:O-H,c:S-H,d:C=O,f:C-S,f:N-H,f:O-H

M0214.stg02;c:C-N,c:N-H,c:O-H,f:N-H,f:O-H,i:C-O

M0214.stg03;c:O-H,c:O-H,d:C=O,f:C-O,f:N-H,f:O-H

M0214.stg04;c:C-S,c:N-H,c:O-H,f:O-H,f:S-H,i:C-O

M0214.stg05;c:C-O,c:N-H,f:C-N,f:O-H

M0215.stg01;c:C-H,c:O-H,d:C=C,d:C=N,f:N-H,f:O-H,i:C-C,i:C-N

M0215.stg02;c:N-H,c:O-H,d:C=C,d:C=N,d:C=O,f:C-C,f:N-H,f:O-H,i:C-C,i:C-N

M0215.stg03;c:C-C,d:C=N,i:C-C,i:C-O

M0215.stg04;c:N-H,c:O-H,d:C=C,f:C-H,f:O-H,i:C-N

M0215.stg05;c:C-C,c:N-H,c:O-H,d:C=C,d:C=N,f:N-H,f:O-H,i:C-C,i:C-N,i:C-O

M0215.stg06;c:N-H,c:O-H,c:O-H,d:C=C,d:C=N,f:C-H,f:N-H,f:N-H,i:C-C,i:C-N

M0216.stg01;d:C=O,f:C-O

M0216.stg02;c:O-H,f:N-H

M0216.stg03;c:N-H,f:O-H

M0217.stg01;c:C-C,c:O-H,f:N-H,f:O-H,i:C-O

M0217.stg02;c:N-H,f:C-H

M0218.stg01;c:O-H,d:C=O,f:C-O,f:N-H

M0218.stg02;c:C-O,c:N-H,f:O-H,i:C-O

M0218.stg03;c:O-H,d:C=O,f:C-O,f:N-H

M0218.stg04;c:C-O,c:N-H,f:O-H,i:C-O

M0219.stg01;c:C-H,c:N-H,d:C=C,d:C=N,f:N-H,f:O-H,i:C-C,i:C-N

M0219.stg02;c:N-H,c:O-H,d:C=C,d:C=N,d:C=O,f:C-C,f:N-H,f:O-H,i:C-C,i:C-N

M0219.stg03;c:C-C,c:O-H,d:C=N,f:N-H,i:C-C,i:C-O

M0219.stg04;c:N-H,d:C=C,d:C=O,f:C-C,f:O-H,i:C-N

M0219.stg05;c:C-C,c:N-H,c:O-H,d:C=C,d:C=N,f:N-H,f:O-H,i:C-C,i:C-N,i:C-O

M0219.stg06;c:N-H,c:O-H,d:C=C,d:C=N,f:C-H,f:N-H,i:C-C,i:C-N

M0222.stg01;d:C=O,f:C-N

M0222.stg02;c:N-H,c:O-H,f:O-H,f:O-H

M0222.stg03;c:C-O,c:O-H,f:O-H,i:C-N

M0222.stg04;c:O-H,c:O-H,f:O-H,f:O-H

M0222.stg05;c:C-H,d:C=N,f:O-H,i:C-C

M0222.stg06;c:O-H,c:O-H,c:O-H,f:O-H,f:O-H,f:O-H

M0222.stg07;c:O-H,d:C=C,d:C=O,f:C-C,f:O-H,i:C-N

M0222.stg08;c:O-H,d:C=N,f:C-O,f:O-H

M0222.stg09;c:C-N,c:O-H,c:O-H,f:N-H,f:O-H,i:C-O

M0222.stg10;c:N-H,c:O-H,c:O-H,d:C=O,f:C-O,f:N-H,f:O-H,f:O-H

**Background Step Bond Changes**

M0003.stg01;c:C-H,d:C=C,d:C=N,d:C=N,d:C=O,f:N-H,i:C-C,i:C-C,i:C-N,i:C-N

M0003.stg02;c:N-H,f:O-H

M0003.stg03;c:N-H,c:O-H,c:O-H,d:C=C,d:C=C,d:C=N,d:C=O,d:C=O,f:N-H,f:O-H,f:O-H,i:C-C,i:C-C,i:C-N,i:C-N,i:C-O

M0003.stg04;c:N-H,c:O-H,f:N-H,f:O-H

M0009.stg01;c:C-O,f:C-S

M0009.stg02;c:O-H,c:P-O,f:O-H,f:P-O

M0012.stg01;c:N-H,f:S-H

M0012.stg02;d:C=O,f:C-N

M0012.stg03;c:C-O,c:N-H,f:O-H,i:C-O

M0012.stg04;c:S-H,f:N-H

M0013.stg01;c:N-H,f:O-H

M0013.stg02;c:N-H,d:C=O,f:C-N,f:O-H

M0013.stg03;c:C-O,c:N-H,f:O-H,i:C-N

M0013.stg04;c:O-H,f:O-H

M0013.stg05;c:C-H,d:C=N,d:C=O,f:O-H,i:C-C,i:C-N

M0013.stg06;c:O-H,d:C=N,f:C-O,f:N-H

M0013.stg07;c:C-N,c:O-H,c:O-H,f:N-H,f:O-H,i:C-O

M0013.stg08;c:N-H,d:C=C,f:O-H,i:C-N,i:C-O

M0013.stg09;c:A-A

M0013.stg10;c:O-H,d:C=N,f:C-O,f:N-H

M0013.stg11;c:C-N,c:O-H,f:N-H,i:C-O

M0013.stg12;c:O-H,c:O-H,f:N-H,f:O-H

M0014.stg01;c:O-H,f:O-H

M0014.stg02;c:V-O,f:V-O

M0014.stg03;c:O-H,f:O-H

M0014.stg04;c:V-O,f:V-O

M0014.stg05;f:O-Cl

M0014.stg06;c:O-H,c:O-O,c:V-O,f:O-H,f:V-O,i:V-O

M0018.stg01;c:C-N,c:S-H,f:C-S,f:N-H

M0018.stg02;c:C-S,c:N-H,c:N-H,f:C-N,f:N-H,f:S-H

M0018.stg03;c:N-H,f:O-H

M0023.stg01;c:N-H,f:O-H

M0023.stg02;c:C-S,f:C-N

M0023.stg03;c:O-H,f:O-H

M0032.stg02;c:C-H,d:C=O,f:O-H,i:C-C

M0032.stg03;c:O-H,f:O-H

M0032.stg04;c:O-H,d:C=C,f:C-H,i:C-O

M0035.stg01;c:O-H,c:P-O,f:O-H,f:P-O

M0035.stg02;c:O-H,f:O-H

M0037.stg01;c:O-H,f:Fe-O,f:N-H

M0037.stg02;c:N-H,c:O-O,f:O-H,i:Fe-O

M0037.stg03;c:O-H,f:R-H

M0037.stg04;c:C-H,d:C=C,f:O-H,i:C-C

M0037.stg05;d:O=O,f:C-O

M0037.stg06;d:C=C,d:C=C,d:C=C,d:O=O,f:C-C,f:C-O,f:C-O,i:C-C

M0037.stg07;c:O-H,f:O-H

M0040.stg01;c:P-O,f:P-O

M0042.stg01;c:O-H,d:P=O,f:N-H,f:P-O

M0042.stg02;c:N-H,c:P-O,f:O-H,i:P-O

M0048.stg01;c:C-O,c:N-H,f:C-N,f:O-H

M0048.stg02;c:O-H,f:O-H

M0052.stg01;c:C-H,d:C=O,f:O-H,i:C-C

M0052.stg02;c:O-H,d:C=C,d:C=O,f:C-C,f:O-H,i:C-O

M0052.stg03;c:O-H,f:O-H

M0060.stg01;c:C-O,c:O-H,f:O-H,i:C-O

M0060.stg02;c:C-H,d:C=O,f:O-H,i:C-C

M0060.stg03;c:O-H,f:O-H

M0060.stg04;c:O-H,d:C=C,f:C-H,f:C-O

M0060.stg05;c:C-N,c:O-H,f:O-H,i:C-O

M0060.stg06;c:O-H,f:N-H

M0063.stg01;c:Co-C,f:Co-N

M0063.stg02;c:C-H,f:C-H

M0063.stg03;c:C-C,c:N-H,f:O-H,i:C-C

M0063.stg04;c:O-H,d:C=C,f:C-C,f:N-H

M0063.stg05;c:C-H,f:C-H

M0063.stg06;c:Co-N,f:Co-C

M0065.stg01;c:N-H,d:C=O,f:O-H,i:C-N

M0065.stg02;c:P-O,f:P-O

M0065.stg03;c:C-O,c:N-H,c:N-H,f:C-N,f:O-H,f:O-H

M0065.stg04;c:O-H,f:N-H

M0080.stg01;c:C-H,c:C-N,c:N-H,c:N-H,d:C=N,f:N-H,f:N-H,f:O-H,i:C-C,i:C-N

M0080.stg02;c:N-H,c:O-H,c:O-H,d:C=N,f:N-H,f:N-H,f:O-H,i:C-N

M0086.stg01;c:N-H,c:P-O,f:O-H,f:P-N

M0086.stg02;c:O-H,f:O-H

M0088.stg01;c:P-O,f:P-N

M0088.stg02;c:P-N,f:P-O

M0095.stg01;c:C-O,c:O-H,c:O-H,f:O-H,f:O-H,i:C-O

M0095.stg02;c:C-H,c:O-H,d:C=O,f:O-H,f:O-H,i:C-C

M0095.stg03;c:O-H,c:O-H,d:C=C,f:C-H,f:O-H,i:C-O

M0095.stg04;c:O-H,f:O-H

M0096.stg01;c:O-H,d:C=N,f:C-O,f:N-H

M0096.stg02;c:N-H,f:N-H

M0096.stg03;c:C-N,c:O-H,f:N-H,i:C-O

M0096.stg04;c:N-H,f:O-H

M0099.stg01;c:O-H,d:C=O,f:C-O,f:O-H

M0099.stg02;c:O-H,f:O-H

M0099.stg03;c:C-H,c:C-O,d:C=O,f:O-H,i:C-C,i:C-O

M0100.stg01;d:C=O,f:C-S

M0100.stg02;c:C-H,d:C=C,d:C=N,f:C-H,i:C-C,i:C-O

M0100.stg03;c:O-H,d:C=O,f:C-O,f:O-H

M0100.stg04;c:C-S,i:C-O

M0100.stg05;c:O-H,f:O-H

M0104.stg01;c:C-H,c:O-H,d:C=O,f:C-H,f:N-H,i:C-O

M0104.stg02;c:N-H,f:O-H

M0104.stg03;c:C-H,d:C=O,f:N-H,i:C-C

M0104.stg04;c:N-H,f:O-H

M0104.stg05;c:O-H,c:O-H,d:C=C,d:C=C,d:C=O,d:C=O,f:O-H,f:O-H,i:C-C,i:C-C,i:C-O,i:C-O

M0118.stg01;c:N-H,c:N-H,c:O-H,d:C=N,d:C=O,f:N-H,f:N-H,f:O-H,f:O-H,i:C-N,i:C-N

M0118.stg02;c:O-H,d:C=C,d:C=N,d:O=O,f:O-H,i:C-N,i:C-O

M0118.stg03;f:C-O

M0118.stg04;c:C-O,c:N-H,f:O-H,i:C-N

M0118.stg05;c:O-H,d:C=N,f:C-O,f:N-H

M0121.stg01;d:Mo=O,f:S-O,i:S-O

M0121.stg02;c:Mo-O,f:Mo-O

M0121.stg03;c:O-H,f:O-H

M0121.stg04;c:O-H,f:O-H,i:Mo-O

M0122.stg01;c:O-H,d:S=O,f:O-H,f:S-S

M0122.stg02;c:S-O,c:S-S,f:S-O

M0122.stg03;c:S-H,c:S-O,f:O-H,f:S-S

M0122.stg04;c:S-S,f:S-S

M0122.stg05;c:S-H,c:S-S,f:S-H,f:S-S

M0122.stg06;c:S-H,c:S-S,f:S-H,f:S-S

M0122.stg07;c:O-H,c:S-H,f:O-H,f:O-H

M0124.stg01;c:O-H,c:O-O,d:O=O,f:Cu-O,f:O-H,i:Fe-O

M0124.stg02;c:N-H,c:O-H,f:O-H,f:O-H

M0124.stg03;c:O-H,f:N-H

M0124.stg04;c:O-H,f:O-H

M0124.stg05;c:N-H,c:O-H,f:O-H,f:O-H

M0124.stg06;c:O-H,f:N-H

M0124.stg07;c:O-H,d:Fe=O,f:O-H

M0124.stg08;c:N-H,c:O-H,f:O-H,f:O-H

M0124.stg09;c:Fe-O,c:O-H,f:N-H

M0124.stg10;c:N-H,c:O-H,f:O-H,f:O-H

M0124.stg11;c:O-H,f:N-H

M0125.stg01;c:Cu-O,c:Cu-O,c:O-H,d:O=O,f:Cu-O,f:Cu-O,f:Cu-O,f:O-H

M0125.stg02;c:O-H,f:O-H

M0125.stg03;c:Cu-O,d:C=C,i:C-O,i:C-O

M0125.stg04;c:O-H,c:O-H,c:O-O,f:O-H,f:O-H

M0125.stg05;d:C=C,i:C-O,i:C-O

M0126.stg01;f:Ni-H

M0126.stg02;c:Ni-O,f:Ni-H

M0126.stg03;c:H-H,c:Ni-H,f:Fe-H,f:S-H

M0126.stg04;c:Fe-O,c:S-H,f:O-H

M0126.stg05;c:O-H,f:Ni-H

M0126.stg06;c:Fe-H,c:Ni-H,c:Ni-H,f:Fe-O,f:Ni-O,f:O-H,f:O-H

M0127.stg01;c:Fe-C,c:O-H,f:Fe-H,f:Fe-H

M0127.stg02;c:O-H,f:S-H

M0127.stg03;c:Fe-H,c:S-H,f:Fe-H,f:H-H

M0127.stg04;c:Fe-H,f:Fe-H

M0127.stg05;c:Fe-H,f:Fe-C,f:Fe-H

M0127.stg06;c:Fe-H,c:Fe-H

M0128.stg01;c:P-O,f:P-O

M0128.stg02;c:C-H,f:O-H

M0128.stg03;d:O=O,f:C-O

M0128.stg04;c:C-O,f:C-O

M0128.stg05;c:C-C,c:O-O,i:C-O,i:C-O

M0130.stg01;c:C-H,d:C=C,d:C=N,d:C=N,f:N-H,i:C-C,i:C-C,i:C-N

M0130.stg02;d:C=C,i:C-N

M0130.stg03;c:O-H,d:O=O,f:Fe-O,f:Fe-O,f:O-H

M0130.stg04;c:O-O,d:C=C,f:C-O,f:C-O

M0130.stg05;c:C-O

M0130.stg06;f:C-O

M0130.stg07;c:N-H,f:R-H,i:C-N

M0130.stg08;c:A-B

M0130.stg09;c:Fe-O,c:Fe-O,c:O-H,f:O-H

M0132.stg01;c:R-H,d:C=C,d:O=O,f:O-H,i:C-N

M0132.stg02;f:C-O

M0132.stg03;c:O-H,d:C=O,f:C-O,f:O-H

M0132.stg04;c:O-O

M0132.stg05;c:C-H,f:N-H

M0132.stg06;c:N-H,f:O-H,i:C-O

M0132.stg07;c:C-O,c:N-H,f:O-H,i:C-N

M0133.stg01;c:Fe-O

M0133.stg02;d:O=O,f:Fe-O

M0133.stg03;c:O-H,f:O-H

M0133.stg04;c:O-H,c:O-O,f:O-H,i:Fe-O

M0133.stg05;c:C-H,d:Fe=O,f:O-H

M0133.stg06;c:Fe-O,f:C-O,f:Fe-O

M0134.stg01;d:O=O,f:Fe-O

M0134.stg02;d:C=C,d:C=N,f:C-O,i:C-N,i:C-N

M0134.stg03;c:N-H,c:O-O,f:O-H,i:Fe-O

M0134.stg04;d:C=C,d:Fe=O,f:C-O

M0134.stg05;c:C-H,c:Fe-O,f:C-H,i:C-O

M0134.stg06;c:C-H,d:C=O,f:O-H,i:C-C

M0135.stg01;c:Cu-O

M0135.stg02;c:Cu-O

M0135.stg03;c:Cu-O,d:O=O,f:Cu-O,f:Cu-O

M0135.stg04;c:C-H,c:Cu-O,f:Cu-O,f:O-H

M0135.stg05;c:O-O,f:C-O

M0135.stg06;c:O-H,f:Cu-O,f:O-H

M0136.stg01;c:A-C

M0136.stg02;c:Fe-O,c:Fe-O

M0136.stg03;d:O=O,f:Fe-O,f:Fe-O,f:Fe-O

M0136.stg04;c:O-H,c:O-H,c:O-O,f:Fe-O,f:O-H,f:O-H

M0136.stg05;c:C-H,c:C-H,c:Fe-O,c:Fe-O,f:O-H,f:O-H,i:C-C

M0137.stg01;d:C=O,d:O=O,f:C-O,f:Fe-O

M0137.stg02;c:C-C,i:C-O,i:C-O

M0137.stg03;c:O-O,i:Fe-O

M0137.stg04;c:C-H,d:Fe=O,f:O-H

M0137.stg05;f:C-S

M0137.stg06;c:C-S

M0137.stg07;c:C-H,c:Fe-O,c:Fe-S,f:O-H,i:C-C

M0138.stg01;c:Cu-N,c:Cu-O,c:O-H,f:N-H

M0138.stg02;c:N-H,c:O-H,d:O=O,f:Cu-N,f:O-H,f:O-H

M0140.stg01;c:Co-C

M0140.stg02;c:S-H,f:C-H

M0140.stg03;c:C-H,f:S-H

M0140.stg04;c:C-O,c:O-H,c:S-H,f:O-H,f:O-H,i:C-O

M0140.stg05;c:S-H,f:C-H,f:S-S

M0140.stg06;c:O-H,d:C=O,f:O-H

M0140.stg07;c:S-H,f:C-H

M0140.stg08;c:C-H,f:S-H

M0140.stg09;f:Co-C

M0140.stg10;c:S-H,c:S-S,f:S-H,f:S-S

M0140.stg11;c:S-H,c:S-S,f:S-H,f:S-S

M0140.stg12;c:S-H,c:S-S,f:S-H,f:S-S

M0140.stg13;c:S-H,c:S-S,f:S-H,f:S-S

M0141.stg01;c:C-H,c:O-H,d:C=C,d:C=C,d:C=N,d:C=N,d:C=O,f:N-H,f:O-H,i:C-C,i:C-C,i:C-C,i:C-N,i:C-O

M0141.stg02;c:N-H,c:O-H,c:O-H,c:O-H,c:O-H,d:C=C,d:C=C,d:C=N,d:C=O,d:C=O,f:C-O,f:N-H,f:O-H,f:O-H,f:O-H,f:O-H,i:C-C,i:C-C,i:C-N,i:C-O

M0141.stg03;c:O-H,f:O-H

M0141.stg04;d:C=C,d:C=N,i:C-N,i:C-O

M0141.stg05;c:N-H,f:O-H,i:C-N

M0141.stg06;c:O-H,f:O-H

M0141.stg07;c:C-H,c:O-H,d:C=N,d:C=N,d:C=O,f:N-H,f:O-H,i:C-C,i:C-N,i:C-O

M0141.stg08;c:O-H,f:O-H

M0141.stg09;d:C=C,d:C=N,i:C-N,i:C-O

M0141.stg10;c:N-H,f:O-H,i:C-N

M0141.stg11;c:O-H,f:O-H

M0142.stg01;c:C-H,d:C=C,d:C=N,d:C=N,f:N-H,i:C-C,i:C-C,i:C-N

M0142.stg02;d:C=C,i:C-N

M0142.stg03;c:N-H,f:O-H,i:C-N

M0143.stg01;c:As-O,c:O-H,f:As-S,f:O-H

M0143.stg02;c:As-S,f:S-S

M0143.stg03;c:S-S,f:S-S

M0143.stg04;c:S-S,f:S-S

M0143.stg05;c:S-S,f:S-S

M0147.stg01;c:N-H,d:C=N,f:C-N,f:N-H

M0147.stg02;c:C-N,i:C-N

M0147.stg03;c:C-C,c:N-H,d:C=C,d:C=N,d:C=N,f:C-N,f:O-H,i:C-C,i:C-C,i:C-N

M0147.stg04;c:C-O,c:O-H,f:O-H,i:C-N

M0147.stg05;c:N-H,c:O-H,d:C=C,d:C=C,d:C=N,d:C=N,f:C-H,f:C-N,f:O-H,i:C-C,i:C-N,i:C-N

M0147.stg06;c:N-H,d:C=N,f:C-N,f:N-H

M0147.stg07;c:C-N,i:C-N

M0149.stg01;d:C=O,f:C-S

M0149.stg02;c:C-N,c:N-H,f:N-H,i:C-O

M0149.stg03;c:N-H,d:C=O,f:C-N,f:N-H

M0149.stg04;c:C-S,i:C-O

M0150.stg01;c:N-H,c:P-O,f:O-H,f:P-N

M0150.stg02;c:O-H,c:P-N,f:N-H,f:P-O

M0151.stg01;c:O-H,c:P-O,f:O-H,f:P-O

M0152.stg01;c:O-H,f:O-H

M0152.stg02;d:P=O,f:P-O

M0152.stg03;c:O-H,c:P-O,d:P=O,f:O-H,i:P-O,i:P-O

M0153.stg01;c:S-S,d:S=O,f:S-S

M0153.stg02;c:S-S,f:C-S

M0154.stg01;c:O-H,c:S-O,f:O-H,f:S-O

M0156.stg01;c:C-S,c:O-H,f:Ni-C,f:S-H

M0156.stg02;c:O-H,f:O-H

M0156.stg03;c:Ni-C,c:S-H,f:C-H

M0156.stg04;f:S-S

M0156.stg05;c:A-D

M0157.stg01;c:C-S,f:C-O

M0157.stg02;c:O-H,f:S-H

M0158.stg01;c:N-H,c:O-H,c:S-O,f:O-H,f:O-H,f:S-O

M0158.stg02;c:C-O,c:O-H,c:O-H,f:N-H,f:O-H,i:C-O

M0158.stg03;c:N-H,c:O-H,d:C=O,f:C-O,f:N-H,f:O-H

M0160.stg01;c:O-H,c:O-H,c:P-O,f:N-H,f:O-H,f:P-O

M0160.stg02;c:N-H,f:O-H

M0162.stg01;c:C-N,d:C=O,f:C-N,i:C-N

M0162.stg02;c:N-H,d:C=N,f:N-H,i:C-O

M0162.stg03;c:C-O,c:O-H,f:O-H,i:C-N

M0162.stg04;c:C-H,d:C=N,f:O-H,i:C-C

M0162.stg05;c:C-O,c:O-H,d:C=C,f:O-H,i:C-C,i:C-N

M0162.stg06;c:O-H,d:C=N,f:C-O,f:O-H

M0162.stg07;c:O-H,f:N-H

M0162.stg08;c:C-N,i:C-O

M0163.stg01;c:O-H,c:O-H,c:P-O,f:N-H,f:O-H,f:P-O

M0163.stg02;c:N-H,f:O-H

M0164.stg01;c:N-H,c:O-H,c:P-O,f:N-H,f:O-H,f:P-O

M0164.stg02;c:N-H,c:O-H,c:P-O,f:N-H,f:O-H,f:P-O

M0165.stg01;c:N-H,c:O-H,c:P-O,f:O-H,f:O-H,f:P-O

M0165.stg02;c:O-H,c:O-H,f:N-H,f:O-H

M0167.stg01;c:O-H,f:O-H

M0167.stg02;d:C=O,f:C-O

M0167.stg03;c:C-N,c:O-H,f:N-H,i:C-O

M0169.stg01;c:O-H,f:N-H

M0169.stg02;d:C=O,f:C-O

M0169.stg03;c:C-N,c:N-H,f:N-H,i:C-O

M0169.stg04;c:O-H,d:C=O,f:C-O,f:N-H

M0169.stg05;c:C-O,c:N-H,f:O-H,i:C-O

M0172.stg01;c:O-H,d:C=O,d:C=O,f:C-O,f:O-H,i:C-O

M0172.stg02;c:C-N,c:O-H,d:C=O,f:N-H,i:C-O,i:C-O

M0173.stg01;c:O-H,d:C=O,f:C-O,f:N-H

M0173.stg02;c:C-N,c:N-H,f:N-H,i:C-O

M0173.stg03;c:O-H,d:C=O,f:C-O,f:N-H

M0173.stg04;c:C-O,c:N-H,f:O-H,i:C-O

M0174.stg01;d:C=O,f:C-S

M0174.stg02;c:C-N,c:N-H,f:N-H,i:C-O

M0174.stg03;c:C-S,c:O-H,f:C-O,f:N-H

M0175.stg01;c:O-H,c:O-H,d:C=O,f:C-O,f:O-H,f:O-H

M0175.stg02;c:C-N,c:O-H,c:O-H,f:N-H,f:O-H,i:C-O

M0176.stg01;c:O-H,d:C=O,f:C-O,f:N-H,f:Zn-O

M0176.stg02;c:C-N,c:N-H,c:Zn-O,f:N-H,i:C-O

M0176.stg03;c:O-H,f:N-H

M0177.stg01;c:O-H,d:C=O,f:C-O,f:N-H

M0177.stg02;c:C-N,c:N-H,f:N-H,i:C-O

M0177.stg03;c:O-H,d:C=O,f:C-O,f:N-H

M0177.stg04;c:C-O,c:N-H,f:O-H,i:C-O

M0179.stg01;c:O-H,d:P=O,f:O-H,f:P-O

M0179.stg02;c:O-H,c:P-O,f:O-H,i:P-O

M0180.stg01;c:O-H,d:C=O,f:C-O,f:N-H

M0180.stg02;c:C-C,i:C-O

M0180.stg03;c:N-H,f:C-H

M0180.stg04;c:N-H,c:O-H,f:N-H,f:O-H

M0181.stg01;d:C=O,f:C-N

M0181.stg02;c:N-H,f:O-H

M0181.stg03;c:C-O,c:N-H,f:O-H,i:C-N

M0181.stg04;c:C-P,d:C=N,f:P-O,i:C-C

M0181.stg05;c:O-H,c:P-O,d:C=C,f:C-H,f:P-O,i:C-N

M0181.stg06;c:O-H,d:C=N,f:C-O,f:N-H

M0181.stg07;c:O-H,f:N-H

M0181.stg08;c:C-N,i:C-O

M0183.stg01;c:A-E

M0183.stg02;c:A-F

M0183.stg03;c:O-H,d:C=C,d:C=O,f:O-H,i:C-N

M0183.stg04;c:C-C,i:C-C

M0183.stg05;c:C-C,d:C=C,i:C-C,i:C-C

M0183.stg06;c:O-H,d:C=N,f:O-H,i:C-C,i:C-O

M0184.stg01;c:C-H,d:C=O,f:N-H,i:C-C

M0184.stg02;c:C-O,c:O-H,d:C=C,f:O-H,i:C-C,i:C-O

M0184.stg03;c:N-H,f:O-H

M0185.stg01;c:C-N,f:C-N

M0185.stg02;c:N-H,f:N-H

M0185.stg03;c:C-O,c:N-H,f:O-H,i:C-N

M0185.stg04;c:C-H,d:C=N,f:N-H,i:C-C

M0185.stg05;c:C-O,d:C=C,i:C-C,i:C-N

M0185.stg06;c:O-H,d:C=N,f:C-O,f:N-H

M0185.stg07;c:C-N,c:O-H,f:O-H,i:C-O

M0186.stg01;c:N-H,c:N-H,d:C=N,f:C-N,f:N-H,f:O-H

M0186.stg02;c:C-N,i:C-N

M0186.stg03;c:C-H,c:C-O,c:O-H,f:N-H,f:O-H,i:C-C

M0186.stg04;c:N-H,c:N-H,d:C=N,f:C-N,f:N-H,f:O-H

M0186.stg05;c:C-N,c:O-H,f:N-H,i:C-N

M0187.stg01;c:C-H,d:C=O,f:N-H,i:C-C

M0187.stg02;c:N-H,d:C=C,f:C-H,i:C-O

M0190.stg01;c:O-H,d:C=C,f:C-H

M0190.stg02;c:C-H,f:S-H,i:C-C

M0190.stg03;c:S-H,f:O-H

M0191.stg01;c:S-S,f:S-S

M0191.stg02;c:S-S,f:S-S

M0191.stg03;c:S-S,f:S-S

M0196.stg01;c:O-H,d:C=C,f:C-H,f:C-O

M0202.stg01;c:P-O,f:P-N

M0202.stg02;c:P-N,f:P-O

M0202.stg03;c:O-H,c:P-O,f:O-H,f:P-O

M0207.stg01;c:P-O,f:P-N

M0207.stg02;c:P-O,f:P-O

M0207.stg03;c:C-H,c:O-H,c:O-H,c:O-H,c:P-N,c:S-H,d:C=O,f:O-H,f:O-H,f:O-H,f:O-H,f:P-O,f:S-H,i:C-C

M0209.stg01;c:O-H,c:O-H,c:P-O,f:O-H,f:O-H,f:P-O

M0209.stg02;c:O-H,f:O-H

M0210.stg01;c:O-H,d:C=O,f:C-O,f:O-H

M0210.stg02;c:C-N,c:N-H,c:O-H,c:O-H,f:N-H,f:N-H,f:O-H,i:C-O

M0210.stg03;c:O-H,d:C=O,f:C-O,f:O-H

M0210.stg04;c:C-O,c:O-H,f:O-H,i:C-O

M0211.stg01;c:C-H,d:C=C,d:C=N,d:C=N,d:C=O,f:N-H,i:C-C,i:C-C,i:C-N,i:C-N

M0211.stg02;c:N-H,c:O-H,d:C=C,d:C=N,d:N=O,f:O-H,f:O-H,i:C-N,i:C-N,i:C-O

M0211.stg03;c:N-O,c:O-H,f:O-H,i:N-O

M0211.stg04;c:C-H,d:C=C,d:C=N,d:C=N,d:C=O,f:N-H,i:C-C,i:C-C,i:C-N,i:C-N

M0211.stg05;c:N-H,c:O-H,d:C=C,d:C=N,d:N=O,f:N-H,f:O-H,i:C-N,i:C-N,i:C-O

M0220.stg01;c:C-H,c:N-H,d:C=C,d:C=N,f:N-H,f:O-H,i:C-C,i:C-N

M0220.stg02;c:N-H,d:C=O,f:C-C,f:O-H

M0220.stg03;c:C-C,d:C=N,i:C-C,i:C-O

M0220.stg04;c:N-H,d:C=C,f:C-H,i:C-N

M0220.stg05;c:C-C,c:O-H,f:N-H,i:C-O

M0220.stg06;c:N-H,c:O-H,c:O-H,d:C=C,d:C=N,f:C-H,f:N-H,f:N-H,i:C-C,i:C-N
