# Supplementary material for: Quantitative Comparison of Catalytic Mechanisms and Overall Reactions in Convergently Evolved Enzymes: Implications for Classification of Enzyme Function
Source: PLoS Comput Biol. 2010 Mar 12;6(3):e1000700. doi: 10.1371/journal.pcbi.1000700 (PMC2837397; doi:10.1371/journal.pcbi.1000700)
Supplement: Text S1 — Mapping between catalytic activities within the molecular function ontology of the Gene Ontology and the codes in the Enzyme Commission classification. (0.06 MB PDF) [file pcbi.1000700.s012.pdf]

Text S1. Mapping between catalytic activities within the molecular function ontology of the Gene Ontology and the codes in the Enzyme Commission classification.

To the best of our knowledge, no formal comparison between the Enzyme Commission (EC) classification and the Gene Ontology's (GO) molecular function classification has been published in the literature. The Gene Ontology website [124], however, offers various datasets and tools that allow us to compare the two resources. As of November 11 2009 the Gene Ontology contained 8659 terms in its molecular function ontology. Using the GO Online SQL Environment (GOOSE) [125], we determined that 5802 of these terms are descendants of the node "catalytic activity." Of these, 3916 (67.5%) are derived from the EC classification, as mapped in EC2GO revision 1.123 [126] (a monthly-updated mapping between EC and GO terms maintained by the GO Consortium). Thus, the catalytic activities defined in the molecular function ontology of the GO are in the most part derived from the EC classification.

124. http://www.geneontology.org/
125. http://www.berkeleybop.org/goose
126. http://www.geneontology.org/external2go/ec2go
